# Transcriptomic Signature of Human Embryonic Thyroid Reveals Transition From Differentiation to Functional Maturation

**DOI:** 10.3389/fcell.2021.669354

**Published:** 2021-06-11

**Authors:** Geneviève Dom, Petr Dmitriev, Marie-Alexandra Lambot, Guy Van Vliet, Daniel Glinoer, Frédérick Libert, Anne Lefort, Jacques E. Dumont, Carine Maenhaut

**Affiliations:** ^1^School of Medicine, IRIBHM, Université libre de Bruxelles, Brussels, Belgium; ^2^Institute of Interdisciplinary Research in Human and Molecular Biology, Brussels, Belgium; ^3^Hôpital Saint-Pierre, Université libre de Bruxelles, Brussels, Belgium; ^4^Département de Pédiatrie, Université de Montréal, Montreal, QC, Canada; ^5^CHU Sainte-Justine, Montreal, QC, Canada; ^6^IRIDIA, Université libre de Bruxelles, Brussels, Belgium

**Keywords:** fetal thyroid, thyroid function maturation, TSH, FGF, IGF1

## Abstract

The human thyroid gland acquires a differentiation program as early as weeks 3–4 of embryonic development. The onset of functional differentiation, which manifests by the appearance of colloid in thyroid follicles, takes place during gestation weeks 10–11. By 12–13 weeks functional differentiation is accomplished and the thyroid is capable of producing thyroid hormones although at a low level. During maturation, thyroid hormones yield increases and physiological mechanisms of thyroid hormone synthesis regulation are established. In the present work we traced the process of thyroid functional differentiation and maturation in the course of human development by performing transcriptomic analysis of human thyroids covering the period of gestation weeks 7–11 and comparing it to adult human thyroid. We obtained specific transcriptomic signatures of embryonic and adult human thyroids by comparing them to non-thyroid tissues from human embryos and adults. We defined a non-TSH (thyroid stimulating hormone) dependent transition from differentiation to maturation of thyroid. The study also sought to shed light on possible factors that could replace TSH, which is absent in this window of gestational age, to trigger transition to the emergence of thyroid function. We propose a list of possible genes that may also be involved in abnormalities in thyroid differentiation and/or maturation, hence leading to congenital hypothyroidism. To our knowledge, this study represent the first transcriptomic analysis of human embryonic thyroid and its comparison to adult thyroid.

## Introduction

### Thyroid Morphogenesis

**FIGURE 1:**
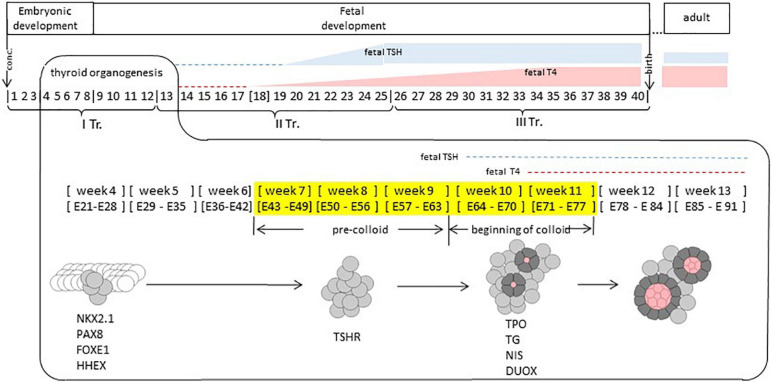
Human thyroid development. Timeline of thyroid development. Thyroid organogenesis covers the embryonic and fetal periods. Thyroid fate determination happens prior to weeks 4. Specific transcription factors (NKX2.1, PAX8, FOXE1, and HHEX) are expressed at week 4. Thyroid progenitor cells migration occurs at weeks 4–7; TSHR (TSH receptor) is expressed at week 8. Thyroid functional differentiation: weeks 7–11, colloid appearing at weeks 10–11, when genes such as TPO, TG, NIS, and DUOX, coding for proteins involved in thyroid function are expressed. Thyroid maturation (GW 12 – 1 months after birth or later). TSH is present at week 10 at low concentration in fetal plasma, at week 20 it increases to reach a maximum level between weeks 20 and 30. Fetal TSH receptors become responsive to TSH around week 20. Week, gestational week. E, embryonic day. TR, gestational trimester. In yellow: gestational weeks covered by our study. Conc, conception.

The thyroid is an endocrine gland of endodermal origin; key events in its development take place early in human development (Figure 1). Thyroid fate is determined prior to week 4 of gestation (^∗^we refer to weeks elapsed from the moment of conception). The thyroid primordium is first observable in the human embryo between embryonic days 20–22 as a thickening in the floor of the pharynx. The proliferation of thyrocyte precursors in the course of week 4 results in the formation of the thyroid bud which adopts a bilobed shape by the end of week 4. In the course of weeks 5–6, the thyroid continues its growth while migrating from the pharyngeal floor and reaches its definitive location in front of the trachea by week 7 (Van Vliet, 2003; De Felice and Di Lauro, 2004; Polak et al., 2004b; Nilsson and Fagman, 2013).

During weeks 7–9 (“pre-colloid” stage) thyroid is composed of unpolarized thyrocyte precursors. The appearance of small colloid-filled follicles formed by polarized thyrocytes is observed during weeks 10–11 (“beginning of colloid” stage) (Szinnai et al., 2007). By weeks 12–13, human thyroid organogenesis is complete, although the thyroid gland continues its growth beyond this time point (De Felice et al., 2004).

### Functional Differentiation of the Thyroid

The appearance of thyroid follicles during the “beginning of colloid” stage coincides with the onset of thyroxine (T4) production by the fetal thyroid, which is observed at 11 weeks and marks the stage of functional (terminal) differentiation (Szinnai et al., 2007). Detection of thyroid hormones is even documented as early as week 10 in the serum (Glinoer and Delange, 2000). Yet, to acquire the full functionality, the thyroid has to transit through the maturation stage. Thyroxine production by the fetal thyroid remains low until mid-gestation, and before this time, the human fetus is dependent on the cross-placental supply of maternal thyroid hormones (Vulsma et al., 1989). Starting from week 18, a steady increase of T4 production by the fetal thyroid is observed with a maximum at weeks 34–36, although the ratio of TSH (thyrotropin) to T4 remains higher than in adults, indicating relatively low responsiveness of the fetal thyroid to TSH, the main regulator of thyroid function. The maturation of the thyroid is achieved only 1 month after birth when the levels of T4 and TSH become similar to those observed in adults (Fisher and Polk, 1989; Fisher et al., 2000).

### Thyroid Development at the Molecular Level

Our current understanding of human thyroid development mechanism at the molecular level is based on the study of model organisms systems such as mouse (Fagman and Nilsson, 2011), zebrafish (Haerlingen et al., 2019), and *in vitro* stem cell systems of mouse and human (Antonica et al., 2012; Kurmann et al., 2015).

A key role in thyroid fate induction has been demonstrated for fibroblast growth factors (FGF) and bone morphogenic proteins (BMP) while retinoic acid (RA) was shown to play an inhibitory role (Fagman and Nilsson, 2011; Kurmann et al., 2015; Nilsson and Fagman, 2017; Haerlingen et al., 2019).

Transcriptional identity of the thyroid progenitor cell is defined by Nkx2-1, Pax8, Hhex, and FoxE1 transcription factors which have been shown to control budding, migration, survival, and terminal differentiation of thyrocyte progenitors in mice model systems (Nilsson and Fagman, 2017). Other genes that are known to contribute to thyroid organogenesis in model systems include Eya, Hoxa3, Hoxb3, Hoxd3, Isl, Shh, and Hes1. In human, mutations in NKX2-1, FOXE1, and IYD were associated with congenital hypothyroidism (CH) (Szinnai, 2014; Mio et al., 2020). In human embryos thyroid, these factors are expressed from the earliest stage studied so far, documented at E32–33 (Trueba et al., 2005; Szinnai et al., 2007) and up to adults (Lacroix et al., 2006).

Thyroid follicle formation in mice has been demonstrated to occur with apico-basal polarization of thyrocyte precursors and was shown to require VEGF-dependent endothelial cell recruitment (Hick et al., 2013) and BMP-Smad signaling (Villacorte et al., 2016).

Genes directly responsible for thyroid hormone production include TG, TPO, NIS, IYD, DUOX2, DUOXA2, PDS/SLC26A4 (Carvalho and Dupuy, 2017). Mutations of these genes are known to be associated with CH (Szinnai, 2014). In the human thyroid, the onset of expression of TG protein was documented at week 7 (pre-colloid stage), while TPO and NIS protein expression appeared later, at week 11 (beginning of colloid stage) (Szinnai et al., 2007).

Thyroid function is predominantly controlled by thyrotropin receptor (TSHR). Biological effects of TSHR activation include stimulation of thyroid hormone production and thyrocyte proliferation and are mediated mainly by the cAMP cascade (Dumont et al., 1992, 2015). Mutations in TSHR are among the most frequent genetic defects associated with CH (Szinnai, 2014; Mio et al., 2020).

In model organisms, TSHR signaling is required for the terminal differentiation of thyrocytes (Postiglione et al., 2002; De Felice et al., 2004; Opitz et al., 2011). While in the adult thyroid TSHR is activated mainly by TSH, its role in thyroid differentiation is limited. TSHR KO mice are normal at birth (Postiglione et al., 2002) and human fetuses harboring mutations in the TSHR are of normal size at birth but develop hypothyroidism and have a smaller thyroid.

In human, the onset of TSHR expression at the protein level was documented in human thyroid at week 7 (Szinnai et al., 2007). The human fetal pituitary starts secreting TSH by week 10 (Fisher and Polk, 1989); its concentration in fetal plasma remains low until week 20 after which it increases to reach a maximum level between weeks 20 and 30 (Figure 1). Fetal TSH receptors become responsive to TSH around week 20 (Polak et al., 2004a). Functional differentiation of the thyroid (weeks 10–11) takes place in the presence of low amounts of TSH and is completed before the establishment of TSHR signaling. This process is thus likely independent (or weakly dependent) from TSHR signaling (Fisher and Polk, 1989).

The mechanisms governing functional differentiation of the human thyroid remain poorly understood although other signaling cascades such as those activated by FGF and IGF are likely to play a role. This question was addressed by the current study, which analyzes fetal thyroid samples covering both the period of functional differentiation and the period immediately preceding it.

The aim of our study was to identify new genes and pathways that may be involved in this process and also to expand our general understanding of thyroid development in the human. To this end, we conducted a transcriptomic analysis of the human thyroid between GW 7–11, covering the period of transition of thyroid from undifferentiated to functionally differentiated state. We also compared the transcriptomes of fetal and adult thyroids to each other and to non-thyroid tissues. This allowed us to establish the transcriptional identity of fetal and adult thyroids but also the evolution of gene expression profiles in the course of thyroid maturation. We performed a functional analysis of the transcriptomic profiles in order to better understand the thyroid maturation process.

Finally, we established the lists of genes following specific expression patterns adopted by known thyroid transcription factors and of genes required for thyroid hormone production. These lists may serve as a source of gene-candidates for the search of mutations potentially linked to CH.

## Materials and Methods

### Thyroid Tissues

Embryonic/fetal thyroid were collected from human embryos after elective termination of pregnancy at 9–13 weeks post amenorrhea (corresponding to 7–11 weeks of gestation) according to a protocol approved by the three relevant Ethics Committees (Erasme Hospital, Université Libre de Bruxelles, and Belgian National Fund for Scientific Research FRS/FNRS) on research involving human subjects. Written informed consent was given by the woman in each case; fetal thyroid material was collected from fetuses used in another study concerning brain development (Lambert et al., 2011). Donors did not have any known thyroid pathology nor other known health anomaly. Thyroid was dissected according to Anupriya and Kalpana (2016) and conserved in liquid nitrogen for subsequent RNA isolation (Supplementary Table 1).

Development staging (determination of the Carnegie stage of the embryo/fetus) was performed using feet, limbs and skull dimensions of the embryo/fetus according to the Carnegie staging data of O’Rahilly and Müller (2010).

Adult thyroid tissues were either morphologically normal thyroid tissues from thyroidectomized papillary thyroid cancer (PTC) patients obtained from the J. Bordet Institute (Brussels, Belgium), or post-mortem thyroid tissues from healthy donors (Clontech Takara #636536). Protocols and consent have been approved by the Ethics Committee of the J. Bordet Institute (protocol number: 1978), in accordance with the Declaration of Helsinki.

#### RNA Preparation

Total thyroid RNA was either purchased from Clontech (RNA from post-mortem normal thyroids pooled from 64 male/female Caucasians ages: 15–61, sudden death), or isolated from nitrogen-frozen samples of embryonic, fetal or adult thyroid tissues.

Frozen thyroid tissues were ground in liquid nitrogen and extracted with Trizol (Invitrogen). RNA quality and purity was measured using spectrophotometry, RNA integrity was verified using an automated gel electrophoresis system (Experion, Bio-Rad).

#### Microarray Hybridization

RNA amplification, cDNA synthesis and labeling were performed following Affymetrix (Santa Clara, CA, United States) protocol: 100 ng of RNA were hybridized on Affymetrix Human Genome U133 Plus 2.0 Array.

All gene expression data are released on GEO under the accession number GSE165706.

#### Transcriptomes From Other Studies

Embryonic tissue mix transcriptomes were from Yi et al. (2010) GSE15744.

Human embryos were obtained after elective termination of pregnancy. Morphologically normal embryos were selected and staged according to the Carnegie stages of development; *n* = 3 for each week of weeks 4–9 of embryonic development. Total RNA was isolated from each whole embryo individually.

Two collections of adult human tissue transcriptomes were used: the first dataset (Tateno et al., 2013) is a combination from 22 tissues of human donors of undisclosed age, health status or sex.

In the second study (Roth et al., 2006), tissue samples were from 10 postmortem donors (five females and five males) all Caucasian and aged from 23 to 53 years, free of chronic disease, death resulting from sudden event. Hybridizations for the three studies were carried out with Affymetrix Human Genome U133plus2 Arrays.

### Transcriptome Analysis

#### Quality Control

Intensities of the hybridization control probes were extracted using spikeInProbes() function from simpleaffy package for R (Wilson and Miller, 2005). Microarrays were retained for the analysis if signals from all control probes were detectable, and their intensity varied in the following order: bioB < bioC < bioD < cre (Affymetrix. GeneChip Expression Analysis. Data Analysis Fundamentals booklet P/N 701190 rev.4).

Quality of individual arrays was evaluated using various metrics that included maximal background intensity values (maxbg),% of probes called present (pp), scaling factors (sf), actin 3′/5′ ratio (actin) and GAPDH 3′/5′ ratio (GAPDH) and RNA degradation products slopes (RNAdeg). These metrics were extracted using maxbg(), percent.present(), sfs(), and ratios() functions from simpleaffy package for R (Wilson and Miller, 2005). Microarrays were retained for the analysis if, within each group their metrics were within the ranges specified in (Jones et al., 2006): maxbg < 190, pp > 37.5%, sf < 4, actin < 5, GAPDH < 4.

To assess the overall quality and compatibility of all datasets used in our study, we used RLE (relative log expression) and NUSE (normalized unsealed standard error) metrics computed using using NUSE() and RLE() functions from affyPLM package for R (Brettschneider et al., 2007). Microarrays were retained for the analysis if their median NUSE and RLE values were within ranges specified in Yue et al. (2017) –0.25 < med RLE < 0.25 0.8 < NUSE MED < 1.2 (Supplementary Figure 1).

#### Principal Component Analysis

Principal component analysis (PCA) has been performed using prcomp() function for R (Becker et al., 1988) with centering and scaling on a matrix of normalized and background-corrected log2-transformed probe intensities. Sample scores were extracted from the resulting pca.object using pca.object$x, variable (probe) loadings were extracted using pca.object$rotation.

#### Heatmaps

To generate a heatmap, a matrix of normalized and background corrected, log2-transformed probe intensities were supplied to heatmap.2 function (Warnes et al., 2016) with scale option set to “row” (values centered and scaled in the row direction) and default methods for clustering (complete agglomerative) and distance calculation (euclidean).

#### Differential Expression

Microarray probe intensity data were obtained in this study (adult and embryonic thyroids) or retrieved from publicly available datasets of embryonic human tissue mix (GSE15744) (Yi et al., 2010) and a collection of human adult tissues (GSE18674) (Tateno et al., 2013). Normalization and background correction were performed using RMA (Irizarry, 2003). Differential expression was calculated using limma (Ritchie et al., 2015).

**FIGURE 2:**
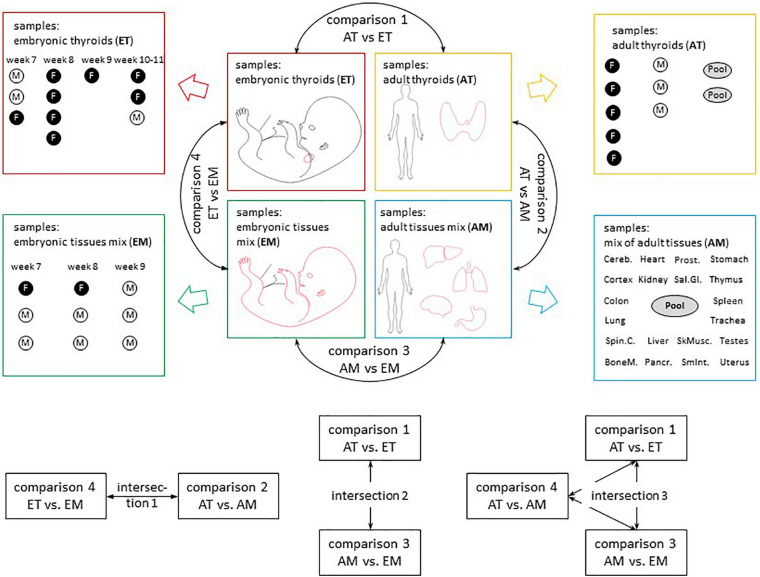
Overview of transcriptomic analyses. Sample composition of the four transcriptomic datasets used in the study (AT, ET, AM, and EM) and comparisons between them. Comparison 1 (AT vs. ET) addressed changes in gene expression observed in the course of thyroid maturation. Comparison 2 (AT vs. AM) and comparison 4 (ET vs. EM) allowed to reveal transcriptional signatures of adult and embryonic thyroids respectively. Comparison 3 (AM vs. EM) addressed changes in gene expression observed in the course of non-thyroid development. M, male. F, female. Week, gestational week. M, male. F, female. Embryonic thyroids (ET) and Adult thyroids (AT): this study. Embryonic tissues mix (EM): Yi et al. (2010), GSE15744. Adult tissues mix (AM): Tateno et al. (2013), GSE 18674.

The comparisons that were carried out are described in Figure 2.

Genes with adjusted *p*-value < 0.05 were classified as “significant”; significant genes with at least twofold change were classified as “differentially expressed.” The status “unchanged” or “not differentially expressed” was assigned to genes with a local FDR value > 0.95. Calculation of local FDR values was performed using OCplus and twilight packages for Bioconductor (Ploner et al., 2006). Genes not fitting into any of these three categories were classified as genes with the uncertain status of differential expression or unmodulated genes (gray area, Supplementary Figure 2). An example of attribution of gene status as a function of parameters of gene expression measured with microarray is depicted on Supplementary Figure 3. Power analysis was performed using size package for Bioconductor (Warnes, 2006). The annotation of microarray probes was done using Carlson M.: hgu133plus2.db: Affymetrix Human Genome U133 Plus 2.0 Array annotation data (chip hgu133plus2*)* R package version 3.2. The status of all genes analyzed for each of the comparisons performed in the study can be found in Supplementary Table 8. The annotation of differentially expressed probes was done using Carlson M.: hgu133plus2.db: Affymetrix Human Genome U133 Plus 2.0 Array annotation data (chip hgu133plus2*)* R package version 3.2.

The strategy and design of the analyses used to define genes commonly regulated in the embryonic and adult thyroid or specifically upregulated in the adult or in the embryonic thyroid (ET) are depicted in Supplementary Figure 7, and in Supplementary Figure 8 to define genes developmentally regulated in the thyroid and in other tissues in general.

### Gene Ontology Analysis

Enrichment analysis of GO terms associated with the lists of differentially expressed genes originating from comparisons 1, 2, and 4 was performed with Database for Annotation, Visualization and Integrated Discovery (DAVID) online tool (version 6.8) (Huang et al., 2009) using Gene Ontology database (version April 2016) (Ashburner et al., 2000). Biological process (BP) – related GO terms with Benjamini-corrected *p*-value < 0.05 were retained for further analysis. To facilitate the interpretation of the resulting list of GO terms, we manually assembled GO terms related to a common biological process into a limited number of groups such as “development,” “proliferation,” which we called “superclusters”. In most cases, each GO term was attributed to a single supercluster, although several GO terms were attributed to several (maximally two) superclusters. Within each supercluster, GO terms describing a more specific function were grouped in a number of subclusters, for example, “epithelium development,” “lung development,” other GO terms were grouped into “general” subcluster. For each subcluster, we created a reference list of genes composed of all gene symbols associated with GO terms included into the given subcluster. The analysis of the lists of differentially expressed genes originating from comparisons 1, 2, and 4 together resulted in the retrieval of 2023 unique significant GO terms which were regrouped into a total of 57 superclusters and 249 subclusters (Supplementary Tables 8, 13). The significance of overlap of the list of differentially expressed genes with the reference list of genes of each subcluster was calculated using Fisher exact test [R function fisher.test()] package statmod v1.4.32 (Giner and Smyth, 2016).

### Real-Time qRT-PCR

Validation of the microarray results were performed by real-time qRT-PCR (SYBR green method) (KAPA SYBR^®^ FAST). The primers were designed with the Primer-3 software^1^. Each reaction was performed in duplicate for each gene. NEDD8 and TTC1 expressions were used to normalize the data (Delys et al., 2007).

### RNA Seq Analyses

Sequencing was performed at the Brussels Interuniversity Genomics High Throughput core^2^. Indexed cDNA libraries were obtained using the TruSeq RNA sample preparation kit (Illumina, CA) following the manufacturer’s recommendations. The multiplexed libraries were loaded on a Novaseq 6000 (Illumina) using a S2 flow cell and sequences were produced using a 200 Cycle Kit. Approximately 20 million paired-end reads per sample were mapped against the human reference genome (GRCh38) using STAR software (version STAR_2.5.3a) to generate read alignments for each sample. Annotations Homo_sapiens GRCH38.90.gtf were obtained from ftp.Ensembl.org. After transcripts assembling, gene level counts were obtained using HTSeq tool (version HTSeq_0.9.1).

Degust^3^ (Powel, 2019), Gorilla^4^ (Eden et al., 2009), and iDEP^5^ tools were used to analyze the data (Ge et al., 2018).

## Results

**FIGURE 3:**
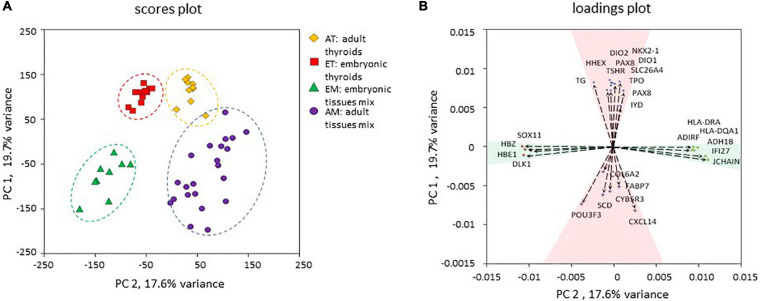
Principal component analysis. **(A)** Principal component analysis of the four transcriptomic datasets used in the study. The position of each data point corresponding to an individual transcriptome is determined by the first principal component (PC 1) and second principal component (PC 2) scores. PC 1 and PC 2 resolved samples according to their thyroid identity and developmental stage respectively. **(B)** Contribution of individual genes to PC 1 and PC 2. Genes with the strongest upregulation in thyroid compared to non-thyroid samples (TPO, TG, TSHR, NKX2-1, PAX8, HHEX, FOXE1, DIO1, DIO2, IYD) and genes with the strongest upregulation in other tissues compared to thyroid (SCD, COL6A2, FABP7, POU3F3, CYB5R3, CXCL14) were among the strongest contributors to the PC 1. Non-tissue-specific genes that demonstrated a higher expression in adult tissues (JCHAIN, HLA-DQA1, ADH1B, ADIRF, HLA-DRA, IFI27) or in embryonic tissues (HBZ, DLK1, HBE, SOX11) contributed more strongly to the PC 2. Genes are depicted as vectors with coordinates corresponding to PC1 and PC2 rotations.

To gain new insights into thyroid development in human, we have analyzed transcriptomes of adult thyroids (AT) and embryonic/fetal thyroids (ET) covering weeks 7–11 of human embryonic development. To extend our analyzes, we included previously published transcriptomes of 20 representative human adult tissues (“adult tissue mix,” AM) and transcriptomes of entire human embryos (“embryonic tissue mix,” EM) covering gestational weeks 7–9 of human embryonic development (Figure 2 and Supplementary Table 1). In total, our analysis included 50 transcriptomes, which were of good quality and could directly be compared to each other for the analysis of differential gene expression, variability, and median intensities being comparable (Supplementary Figure 1). Principal component analysis (PCA) of the transcriptomes demonstrated that the four groups of transcriptomes could efficiently be resolved using only the first two principal components to which a clear biological meaning could be attributed: “thyroid identity” to the first principal component (PC 1) and “developmental stage” to the second principal component (PC 2) (Figure 3).

Differential expression analysis was conducted for four pairwise comparisons of groups of transcriptomes AT vs. ET (comparison 1); AT vs. AM (comparison 2); AM vs. EM (comparison 3); ET vs. EM (comparison 4) (Figure 2). Lists of differentially expressed genes for each of these comparisons including protein-coding, non-coding RNA genes and pseudogenes is presented in Supplementary Table 9. Comparison of the expression level of top differentially expressed genes across all samples analyzed revealed the existence of biologically meaningful groups of genes relevant to various aspects of thyroid development such as genes specifically upregulated both in adult and embryonic thyroids compared to non-thyroid tissues, or genes upregulated in the course of development of the thyroid but not of other organs (Supplementary Figure 4). To obtain full lists of genes that belong to these groups, we performed intersections of lists of differentially expressed genes obtained from comparisons 1–4 (Figure 2). Specifically, intersection 1 was used to characterize similarities and differences of transcriptional identities of adult and embryonic thyroids. Intersection 2 allowed to obtain the list of thyroid-specific developmentally regulated genes. Finally, intersection 3 was used for the identification of genes following patterns of expression specific to genes with known function in the thyroid.

### Thyroid Transcriptome Evolution in the Course of the Human Development

**FIGURE 4:**
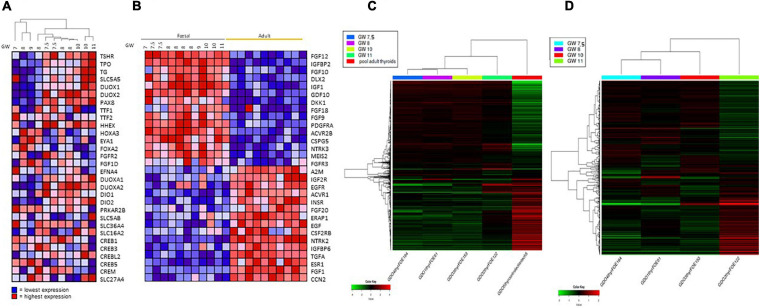
**(A)** Hierarchical clustering of mRNA expression levels of thyroid function proteins in fetus samples. Hierarchical clustering and heat map of expression ratios of genes involved in thyroid function in fetal thyroids versus the pool of normal adult thyroid samples (CLONTECH). For each gene (row), the expression ratio ranges between lowest in dark blue to highest in dark red. **(B)** Heat Map: expression of growth factors and receptor signaling proteins in fetal and adults thyroids. For each gene (row), the expression ratio ranges between lowest in dark blue to highest in dark red. GW, gestational week. **(C)** Hierarchical clustering of the RNAseq expression data of the 2000 most variable genes in four fetus thyroid samples ranging from GW 7.5 to GW 11, and in one adult thyroid sample. Hierarchical clustering and heat map of expression (in CPM -Counts Per Million-) of the 2000 most variable genes in fetal thyroids (GW: 7.5, 8, 10, 11) versus the pool of normal adult thyroid samples (CLONTECH). For each gene (row), the expression ratio ranges between lowest in dark green to highest in dark red. **(D)** Hierarchical clustering of the RNAseq expression data of the 2000 most variable genes in four fetus thyroid samples ranging from GW 7.5 to GW 11. Hierarchical clustering and heat map of expression (in CPM -Counts Per Million-) of the 2000 most variable genes in fetal thyroids (GW: 7.5, 8, 10, 11). For each gene the expression ratio ranges between lowest in dark green to highest in dark red.

Embryonic thyroid samples used in our study covered the period of the beginning of the functional differentiation of the thyroid including “pre-colloid” stage (weeks 7–9) and “onset of colloid” stage (weeks 10–11). Hierarchical clustering performed with thyroid function genes expression from the microarray data organized the fetal thyroid samples according to the gestational age almost perfectly (Figure 4A). When performed with the 2000 most regulated genes derived from the RNA seq data, hierarchical clustering gave similar results (Figure 4C).

Comparing the pre-colloid and beginning colloid stages did not allow us to identify any differentially expressed genes, due to insufficient statistical power. Nevertheless, some genes demonstrated a tendency for upregulation at weeks 10–11 compared to weeks 7–9 (Supplementary Table 2). Figure 4D shows a clustering of the 2000 most regulated genes in the fetal thyroid samples derived from the RNAseq data, excluding the ATs. Fetal thyroid samples remain remarkably ranked according to age, and show a clear shift in global gene expression between GW 10 and GW 11. Also, the pre-colloid (GW 7.5 and 8) and colloid (GW 10 and 11) stages tend to show distinct expression profiles. When analyzing the RNA seq data with the Gorilla software, considering the genes modulated between the pre-colloid (GW 7.5 and 8) and colloid (GW 10 and 11) stages, an enrichment in GO categories such as “G protein-coupled receptor signaling” and “adenylate cyclase-modulating G protein-coupled receptor signaling pathway” was identified. Both are related to the onset of thyroid function (Supplementary Table 4).

More dramatic changes in gene expression levels were observed when ET 7–9 weeks of age was compared to AT (comparison 1 on Figure 2). This analysis allowed to characterize genes expression changed in the course of maturation stage of thyroid development. 2908 differentially expressed genes were identified, of which 1338 were up- and 1570 downregulated (Supplementary Table 9). Strikingly, the expression levels of genes encoding many growth factors and receptors were clearly very different between adult and fetal thyroids: for instance, the high expression of IGF1 and FGFs in fetal thyroids is of particular interest (Figure 4B). For some of these genes, we also observed a gradation of their expression level according to the age of the fetus, as for IGF1 and FGF 10 whose expression is higher in the “onset of colloid stage than in “pre-colloid” one, and for IGFBP and EGF that are less expressed in fetal thyroids.”

**FIGURE 5:**
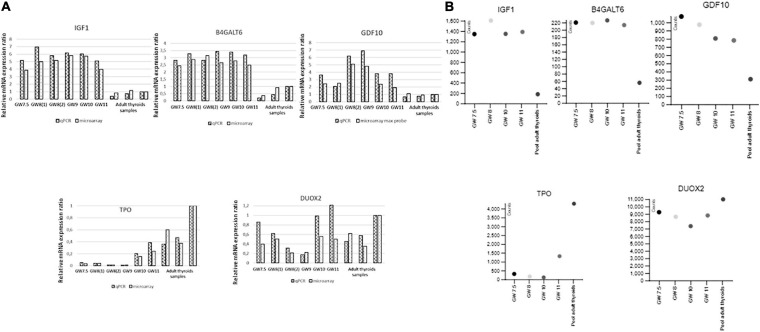
**(A)** Validation by qRT-PCR of the microarray data for IGF1, GDF10, B4GALT6, TPO, and DUOX 2. Ratios of gene expression in qRT-PCR and microarray experiments for different embryonic thyroid samples compared to a pool of adult thyroids (Clontech). Samples are labeled as in Supplementary Table 1: GW 7.5: ET2, GW 8(1): ET4, GW 8(2): ET6, GW 9: ET8, GW 10: ET9, GW 11: ET11 adult thyroid samples: BO148N BO147N and CLONT (GW: gestational week). **(B)** Validation by RNAseq of the microarray data for IGSF1, FGF 12, TSHR, DUOX, and SLCA5A. Expression of genes in counts (counts per 20 million of reads) from RNAseq experiments performed on four fetal thyroid samples and on the pool of adult thyroids (Clontech), labeled as in Supplementary Table 1: GW 7.5: ET 3, GW 8: ET 5, GW 10: ET 10, GW 11: ET 11 (GW: gestational week).

Similar modulation patterns were found by qRT-PCR for the genes that we selected for such confirmations, among which thyroid specific genes and growth factors that are upregulated in fetal thyroids (Figure 5A and Supplementary Figure 5A). Expressions values from the RNA seq data also perfectly fitted with the microarray and qRT-PCR data (Figure 5B and Supplementary Figure 5B).

**FIGURE 6:**
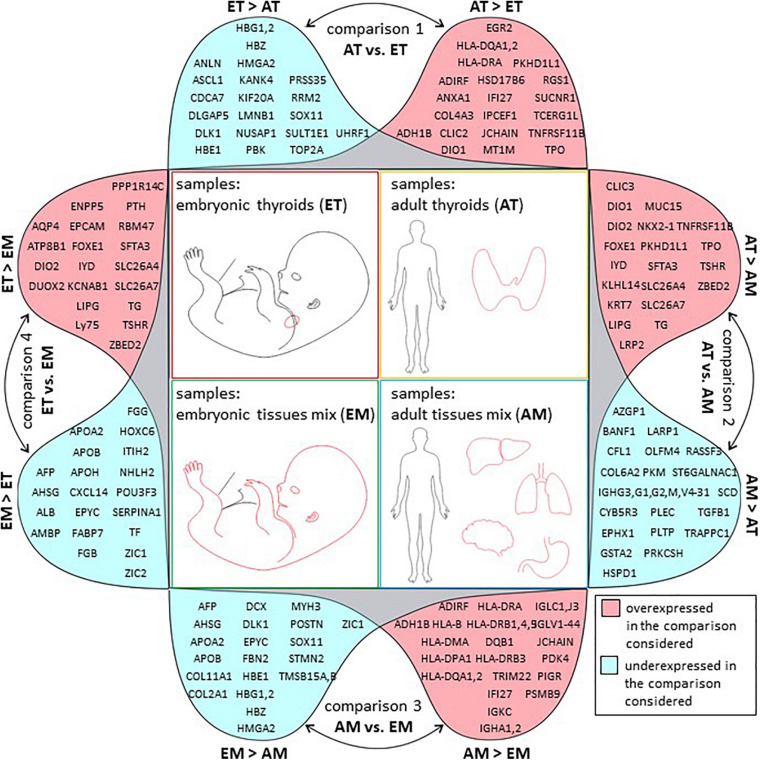
Top 20 regulated genes in all comparisons. Graphical representation of tables of top 20 protein-coding genes with strongest up (in pink) or downregulation (in blue) in each of the four comparisons of transcriptome datasets described in this study. Comparison 1: transcriptomes of adult thyroids (AT) and embryonic thyroids (ET), comparison 2: transcriptomes of adult thyroids (AT) and adult tissues mix (AM), comparison 3: transcriptomes of adult tissues mix (AM) and embryonic tissues mix (EM), comparison 4: transcriptomes of embryonic thyroids (ET) and embryonic tissues mix (EM).

GO analysis of full lists of differentially expressed genes is presented in Supplementary Table 15. The list of top 20 protein-coding genes (Figure 6) is also available in Supplementary Table 5.

### Transcriptional Signatures of Adult and Embryonic Thyroids

#### Adult Thyroids

To obtain the transcriptional profiles of ATs, we carried out a differential gene expression analysis between AT and adult non-thyroid tissues (AM) transcriptomes (comparison 2 on Figure 2). This comparison resulted in a list of 5320 differentially expressed genes of which 3344 genes were up- and 1976 downregulated in AT (Supplementary Table 9). Among the top 20 protein coding genes (Figure 6 and Supplementary Table 5) a number of well-known thyroid-related factors were found: transcription factors: FOXE1, NKX2-1; genes involved in various stages of production of thyroid hormones: TG, TPO, IYD, DIO1, DIO2; thyrotropin receptor*:* TSHR*;* genes involved in ion transport*:* SLC26A4 (pendrin), a multifunctional anion exchanger, suggested to be involved in mediating iodide efflux (Silveira and Kopp, 2015), and SLC26A7 a paralog of pendrin. Some mutations of these genes have recently been correlated with CH (Cangul et al., 2019). Other genes such as HHEX, PAX8 were also found among differentially expressed genes but were not present in the list of the top 20 most regulated genes.

Overall, our differential gene expression analysis included most of the genes known to be involved in thyroid function. Our top 20 list includes genes not so well-known in a thyroid context or emerging thyroid-related factors such as LRP2, SLC26A7, SFTA3, as well as genes not previously associated with thyroid function or development such as CLIC3, PKHD1L1, TNFRSF11B, LIPG, KLHL14, KRT7, MUC15, whose function was extensively studied in other tissues. Among known functions of the top 20 genes were signaling, transport, cytoskeleton and ECM. Genes with still unknown function are also present, such as ZBED2. GO analysis of full lists of differentially expressed genes is presented in Supplementary Table 14.

#### Embryonic Thyroids

To obtain the transcriptional profiles of ETs, we carried out a differential gene expression analysis between ET and embryonic non-thyroid tissues (EM) corresponding to weeks 7–9 of development (comparison 4 on Figure 2). This analysis revealed 4832 differentially expressed genes of which 3200 were up and 1632 downregulated (Supplementary Table 9).

As for the AT transcriptomes, among the top 20 protein coding genes (Figure 6 and Supplementary Table 5) we also found a large number of thyroid-related genes (FOXE1, TG, IYD, DIO2, DUOX2, TSHR, SLC26A7, SLC26A4). Among the top 20 protein coding genes with the strongest level of upregulation in the ETs compared to other embryonic tissues, some were also found enriched in the ATs compared to other adult tissues such as DIO2, FOXE1, IYD, LIPG, SFTA3, SLC26A4, SLC26A7, TG, TSHR, ZBED2. Besides five genes with well-known function in the thyroid (TG, DIO2, DUOX2, TSHR, and SLC26A4), we found a number of genes whose function is less known in the thyroid including SFTA3, SLC26A7, or without any known direct functional association with the thyroid function development, physiology, or production of thyroid hormones including AQP4, ATP8B1, ENPP5, EPCAM, KCNAB1, LIPG, LY75, PPP1R14C, PTH, RBM47, and genes without any known function such as ZBED2. Previously, in an integrative transcriptomic atlas of organogenesis in human embryos, FOXE1, TG, PAX8, DUOX2, DUOXA1, DUOXA2, DUOX1, TPO, DIO2, IYD were identified among the most significant genes upregulated in ETs (Gerrard et al., 2016). GO analysis of full lists of differentially expressed genes is presented in Supplementary Table 14.

### Comparison of Adult and Embryonic Thyroid Expression Profiles

#### Coregulated Genes

Global analysis of gene expression changes resulting from comparison 2 and 4 demonstrated that they are positively correlated with each other (Supplementary Figure 6A). Intersection of lists of differentially expressed genes resulting from comparison 2 and 4 (intersection 1 on Figure 2) indicated that a large proportion of genes were regulated in the same direction in adult and embryonic thyroids while the number of oppositely regulated genes was low (Supplementary Table 10). Top 20 protein coding genes coregulated in adult and embryonic thyroid are presented in Supplementary Table 6.

#### Genes Specifically Enriched in Adult Thyroids

To gain insights into the differences between embryonic and ATs, we then explored how the gene expression profiles may explain the known differences in the function of the adult and embryonic thyroids. To this end, our main focus of attention was the genes with opposite-regulation patterns in the adult and embryonic thyroids because they could shed light on the difference between adult and embryonic thyroid function (Supplementary Figure 6).

Firstly, we looked for the genes specifically upregulated in the ATs that were downregulated or not differentially expressed in the ETs compared to the embryonic tissues mix. Overall, there were 512 unique genes differentially expressed and upregulated in the ATs compared to the adult tissues mix but not in the in ETs compared to the embryonic tissues mix (Supplementary Table 10). Examination of the top 20 list of the most highly upregulated genes in the ATs did not revealed any known thyroid specific gene. The genes present in this list are known for their association with transport, solute channels, signaling, cytoskeleton and ECM (Table 1A).

**TABLE 1A:** Genes specifically enriched in the adult thyroids.

Gene symbol	Gene description	Function
ART4	ADP-ribosyltransferase 4 (Dombrock blood group)	Transport
BHLHE41	Basic helix-loop-helix family member e41	Gene expression
CLIC2	Chloride intracellular channel 2	Channel
CRABP1	Cellular retinoic acid binding protein 1	Signaling
CRYBG3	Crystallin beta-gamma domain containing 3	Cytoskeleton/ECM
DGKI	Diacylglycerol kinase iota	Signaling
DMRT3	Doublesex and mab-3 related transcription factor 3	Gene expression
EPHA3	EPH receptor A3	Signaling
IGFBPL1	Insulin like growth factor binding protein like 1	Signaling
LRP8	LDL receptor related protein 8	Transport
MPPED2	Metallophosphoesterase domain containing 2	Signaling
MT1F	Metallothionein 1F	Transport
NEBL	Nebulette	Cytoskeleton/ECM
OMD	Osteomodulin	Cytoskeleton/ECM
PDE10A	Phosphodiesterase 10A – > inhibition of cAMP signaling?	Signaling
RBMS3	RNA binding motif single stranded interacting protein 3	Gene expression.
RNF128	Ring finger protein 128, E3 ubiquitin protein ligase	Transport
SCUBE3	Signal peptide, CUB domain and EGF like domain containing 3	
SERTM1	Serine rich and transmembrane domain containing 1	
SUCNR1	Succinate receptor 1	

*Top 20 genes regulated in adult thyroids compared to adult tissues mix and not regulated in embryonic thyroids compared to embryonic tissues mix.*

#### Genes Specifically Enriched in Embryonic Thyroids

Conversely, the list of genes upregulated exclusively in ETs but not in ATs, where their expression is weaker or similar to the non-thyroid tissues contained 430 unique genes (Supplementary Table 10). Top 20 protein coding genes of this list are presented in Table 1B.

**TABLE 1B:** Genes specifically enriched in the embryonic thyroids.

Gene symbol	Gene description	Function
ATP11A	ATPase phospholipid transporting 11A – > membrane transporter	Transport
BLNK	B-cell linker	Signaling
CHRNA9	Cholinergic receptor nicotinic alpha 9 subunit	Signaling
CYP4 × 1	Cytochrome P450 family 4 subfamily X member 1	Oxidoreductase
FREM3	FRAS1 related extracellular matrix 3	Cytoskeleton/ECM
GCM2	Glial cells missing homolog 2	Gene expression
GPR160	G protein-coupled receptor 160	Signaling
KCNK5	Potassium two pore domain channel subfamily K member 5	Channel
LUZP1	Leucine zipper protein 1	
MAP4	Microtubule associated protein 4	Cytoskeleton/ECM
NPFFR2	Neuropeptide FF receptor 2	Signaling
OLFM3	Olfactomedin 3	Cytoskeleton/ECM
PCDH20	Protocadherin 20	Cytoskeleton/ECM
PERP	PERP, TP53 apoptosis effector	
PSMB8	Proteasome subunit beta 8	Transport
SAMHD1	SAM and HD domain containing deoxynucleoside triphosphate triphosphohydrolase 1	
SLC15A2	Solute carrier family 15 member 2	Channel
SLC22A3	Solute carrier family 22 member 3	Channel
TNFRSF11A	TNF receptor superfamily member 11a	Signaling

*Top 20 genes regulated in embryonic thyroids compared to embryonic tissues mix, and not regulated in adult thyroids compared to adult tissues mix.*

#### Functions of the Genes Specifically Enriched in Embryonic and Adult Thyroids

**FIGURE 7:**
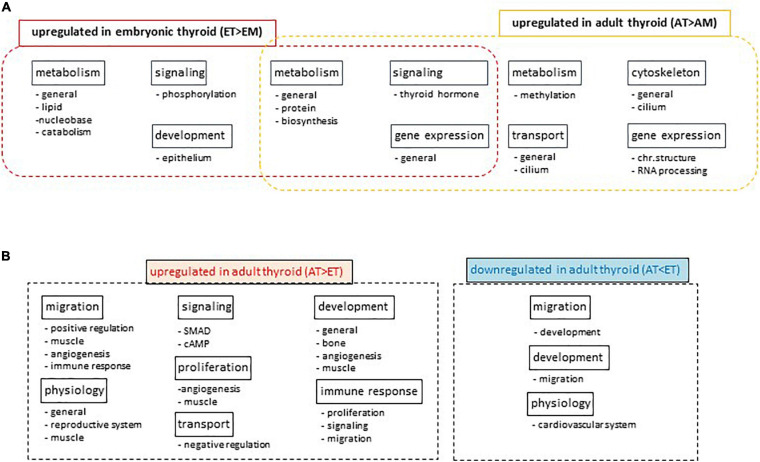
Gene Ontology analyses. **(A)** Differential Gene Ontology analysis of lists of differentially expressed genes upregulated in adult thyroid samples compared to adult tissues mix (AT > AM) or in embryonic thyroid samples compared to embryonic tissues mix (ET > EM). Names of super-and subclusters of GO terms common and specific to adult and embryonic thyroids are shown. **(B)** Differential Gene Ontology analysis of lists of differentially expressed genes developmentally up- or downregulated specifically in the thyroid. Diagrams show the names of super-and subclusters of GO terms enriched in lists of genes differentially expressed in adult thyroids compared to embryonic thyroids (AT vs. ET) in comparison to the lists of genes differentially expressed in adult tissue mix compared to embryonic tissue mix (AM vs. EM).

Differential analysis of GO term enrichment in the lists of differentially expressed genes upregulated in AT compared to AM (comparison 2) and ET compared to EM (comparison 4) showed that adult and embryonic thyroids shared superclusters of GO terms related to metabolism, gene expression and signaling. A specific enrichment for the functional category of transport was observed in ATs, and of development in ETs (Figure 7A). Details of this analysis are presented in Supplementary Table 14.

### Developmental Regulation of Gene Expression in the Thyroid

#### Comparing Adult Thyroids and Embryonic Thyroids by Removing the Overall Developmental Signal (Intersection 2 on Figure 2)

To define the specific program of thyroid development, we carried out intersection of lists of genes differentially expressed in AT compared to ET (comparison 1 on Figure 2) and the list of differentially expressed genes obtained from the comparison of adult mixed tissues (AM) to embryonic mixed tissues (EM) corresponding to weeks 7–9 of human embryonic development (comparison 3 on Figure 2). The latter comparison resulted in the identification of 4940 differentially expressed genes. Of these, 2466 were up- and 2474 were downregulated (Supplementary Table 9, top 20 protein coding genes are presented in Supplementary Table 7).

Global analysis of gene expression changes resulting from comparison 1 and 3 demonstrated that they are positively correlated with each other (Supplementary Figure 6B). Indeed comparison of top genes from the lists of genes upregulated in the thyroids and in other tissues in the course of development showed that these lists are similar. For example, immunity-related genes HLA-DQA1,2, HLA-DRA, IFI27, JCHAIN were among the most highly overexpressed genes both in thyroids and non-thyroid adult tissues. Alcohol dehydrogenases (ADH1B, ADIRF) were also known to be expressed at higher levels in adults. The developmental program of thyroid thus partially reflects the general development program of the human organism.

As in the present work we were particularly interested in genes that are specifically regulated in the course of thyroid development compared to the development of non-thyroid tissues, we excluded from consideration the genes regulated both in the thyroid and non-thyroid tissues in the course of development (Supplementary Table 11 and Supplementary Figure 8). Table 2 lists these specific thyroid developmental genes. They mainly belong to the functional categories of transport, channel and signaling.

**TABLE 2:** Top 20 genes differentially regulated in the course of thyroid development as compared to the development of non-thyroid tissues.

Gene symbol	Description	Function	logFC AT/ET	fdr min	logFC AM/EM	fdr_min
ADGRF1	Adhesion G protein-coupled receptor F1	Signaling	3.059	5,02E-07	–0.313	0.06
ANXA1	Annexin A1		4.562	5.03E-07	0.163	1
ANXA9	Annexin A9		3.042	5,81E-06	0.469	1
ART4	ADP-ribosyltransferase 4 (Dombrock blood group)	Transport	3.602	6.02E-07	–0.322	1
CCDC85A	Coiled-coil domain containing 85A		3.276	6.88E-06	–0.450	0.01
CLIC6	Chloride intracellular channel 6	Channel	3.342	4,15E-04	0.303	1
CTGF	Connective tissue growth factor	Signaling.	3.056	1,75E-05	–0.329	1
DAPL1	Death associated protein like 1		4.154	5.03E-07	0.008	1
DGK1	Diacylglycerol kinase iota	Signaling	4.057	5.03E-07	–1.409	0.02
DIO1	Iodothyronine deiodinase 1	Thyroid hormone	5.533	1.04E-06	1.270	0.7
ESR1	Estrogen receptor 1	Gene expression	3.667	1.77E-05	–0.637	0.001
GPM6A	Glycoprotein M6A		4.184	0.002	–2.452	0.05
MT1F	Metallothionein 1F	Transport	3.617	6.38E-07	–1.263	0.12
MT1G	Metallothionein 1G	Transport	3.910	1.00E-06	–1.500	0.05
MT1H	Metallothionein 1H	Transport	3.411	1.16E-06	–1.200	0.09
OMD	Osteomodulin	Cytoskeleton/ECM	3.468	5.16E-07	–0.382	1
PKHD1L1	PKHD1 like 1		4.736	5.03E-07	0.159	1
SCNN1A	Sodium channel epithelial 1 alpha subunit	Channel	3.378	0.003	–0.934	1E-05
TNFRSF11B	TNF receptor superfamily member 11b	Signaling	6.498	5.03E-07	0.318	1
TPO	Thyroid peroxidase	Thyroid hormone	5.091	5.03E-07	0.010	1

*Genes that are regulated in adult thyroids compared to embryonic thyroids, but not in adult tissues mix compared to embryonic tissues mix are listed. logFC AT/ET, log fold change when comparing adult versus embryonic thyroids. logFC AM/EM, log fold change when comparing adult versus embryonic tissues mix. Fdr, false discovery rate.*

Differential GO analysis showed that superclusters of GO terms related to transport, signaling, immune response, proliferation, development, migration, physiology were enriched in the list of genes upregulated in the ATs. Conversely, list of genes specifically upregulated in fetal thyroids and thus downregulated in ATs was enriched in superclusters of GO terms related to retinoic acid, adipocyte development and cardiovascular system physiology (Figure 7B). Details of this analysis are presented in the Supplementary Table 15.

#### Patterns of Gene Expression in Thyroid Development

Next we thought to search for genes with potential implication in various steps of thyroid development based on specific pattern of expression that they followed in the course of development in thyroid and non-thyroid tissues. To this end, we intersected lists of differentially expressed genes obtained from comparisons 1, 3, and 4 (intersection 3 on Figure 2 and Supplementary Table 12).

**FIGURE 8:**
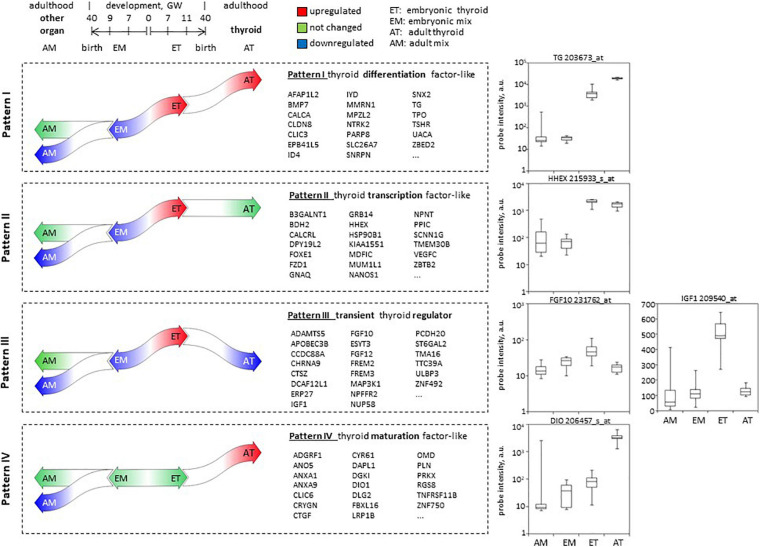
Patterns of developmental gene expression in human thyroid. Schema of gene expression level changes in the course of development of the thyroid and other organs, defining patterns I–IV of expression. In the middle: lists of top 20 protein-coding genes corresponding to each pattern. Complete lists can be found in Supplementary Table 3. To the right: boxplots illustrating hybridization intensity of microarray probes corresponding to one gene representative of each pattern of expression.

Overall, we have defined 4 patterns most relevant to thyroid development (Figure 8). Patterns I, II, and III included genes upregulated in ET compared to embryonic tissues mix (EM) and at the same time downregulated or not differentially expressed in the adult tissues mix (AM) compared to the embryonic tissues mix (EM).

(1) Pattern I included 43 protein-coding genes that in addition to these criteria were upregulated in AT compared to ET. Among the genes following this pattern are known actors of thyroid function (TG, TPO, TSHR, SLC26A7, IYD), or involved in thyroid differentiation such as BMP7 we thus called this pattern “similar to thyroid specific differentiation factors.”
(2) Pattern II that we called “similar to thyroid transcription factor” included 233 protein-coding genes that maintained the same level of expression in adult compared to embryonic thyroids (AT vs. ET) Genes following this pattern are known transcription factors such as HHEX or FOXE1.
(3) Pattern III included 261 protein-coding genes downregulated in AT compared to ET. None of well-known thyroid-related genes followed this pattern of expression which is expected to be adopted by genes playing a role of transient thyroid regulator of thyroid development. We called this pattern III “transient regulator like.” For instance, FGF10 and IGF1 follow this expression pattern, they are candidate genes that could replace TSH signaling to induce thyroid function.
(4) In opposite to patterns I–III, pattern IV assembled 166 protein-coding genes that were not changed in ET compared to embryonic tissues mix (EM) and in the course of development were upregulated in (AT, while down or not regulated in other organs. We expected genes involved in thyroid maturation followed this pattern which we thus called “maturation factor like.”

### Candidate Genes Involved in the Onset of Thyroid Function: Lessons From SNP and Mutations Involved in Congenital Hypothyroidism or Thyroid Function

We extracted lists of genes from publications reporting SNP or loci and mutations involved either in CH (Porcu et al., 2013; Medici et al., 2015, 2017; Taylor et al., 2015; Kuś et al., 2020), or in variations in TSH and/or T4 levels, and from publications describing transcriptomics of adult (Vitale et al., 2017) or embryonic (Gerrard et al., 2016) thyroids. By crossing those lists with our lists of differentially expressed genes specifically enriched in the adult or embryonic thyroids (Table 1), of genes differentially regulated in the course of thyroid development (Table 2), of pattern of genes (Figure 8), and of genes upregulated in the ETs at gestational weeks 10–11, we identified genes in common that could reasonably be interpreted as potential candidates involved in the onset of thyroid function (Supplementary Table 3).

Genes with unknown function in the HPT axis were identified that deserve to be studied, as well as genes encoding proteins involved directly or indirectly in the TSH receptor/cAMP signaling cascade, such as PDE8B, PDE10A, and PRKX. These genes are particularly interesting because they are present both in the list of genes containing SNP related to TSH and T4 levels and in the list of differentially expressed genes in our data. Some other genes that are regulated in our data such as SLC26A7 and IYD were found to be mutated in dysfunctional thyroids leading to CH. Similarly, IGSF1 (leads to central hypothyroidism), SFTA3 and GLIS3, whose mutations are linked to CH, were identified. Also, SLC5A5, the sodium iodide transporter present in our pattern IV “maturation” causes CH when mutated (Pohlenz et al., 1998).

## Discussion

Despite the important development of methods allowing transcriptome profiling, the transcriptomic profile of the developing human thyroid remains uncharacterized.

The timeline of human thyroid development can be divided into four periods (Figure 1):

(1) thyroid fate determination (prior to GW 4) (Polak et al., 2004b)
(2) thyroid progenitor cells migration (GW 4 –7)
(3) thyroid functional differentiation (GW 7 – 11)
(4) thyroid maturation (GW 12 – 1 months after birth or later).

Using transcriptomic analysis, the present study covered an interesting window of time of thyroid embryonic and fetal development in human, that is weeks 7–11 of embryonic development, which corresponds to the establishment of thyroid function. In human, this period of thyroid development remains uncharacterized from the point of view of gene expression. Our transcriptomic analyses allow to discriminate between genes that play a role in thyroid functional differentiation or maturation in the fetus, or in thyroid regulation in adults. This discrimination cannot be achieved by studies associating normal and pathological variations of the thyroid function or gross developmental abnormalities with genetic variants –polymorphisms, mutations (Porcu et al., 2013; Taylor et al., 2015; Kuś et al., 2020).

Our results may therefore provide the basis for a better understanding of various processes that take place during the course of thyroid development (i.e., cell polarization, folliculogenesis, thyroid hormone production, sensitivity to TSH). Our results might also help elucidate which are the molecular mechanisms explaining the onset of thyroid function in the absence of TSH. Indeed, this hormone is not produced, or only at very low concentration, in the embryo until 18–25 gestational weeks and maternal TSH does not cross the placenta, although the fetal thyroid starts producing thyroid hormones at 11 weeks of gestation.

Key observations of gene expression changes in our study were made using microarray-based transcriptome profiling and validated using RNA-seq and qRT-PCR. Previous studies have demonstrated a good agreement between RNA-seq and microarray results (Swindell et al., 2014; Wang et al., 2014; Zhao et al., 2014).

Currently, RNA-seq, a technologically more advanced and versatile approach, is the preferred method of transcriptome profiling as compared to microarray. However, despite its limitations, using the microarray approach as a principal method of transcriptome profiling allowed us to include in the analysis transcriptome profiles (i.e., embryonic and adult non-thyroid tissues) obtained earlier by others.

Previously, it has been argued that the absence of a standard method of Illumina RNA-seq library preparation may undermine the validity of the direct comparison of RNA-seq datasets generated with different protocols (Rao et al., 2019; Sarantopoulou et al., 2019). Conversely, sample preparation and hybridizations to Affymetrix arrays generally conducted using a standard protocol, often allows for a direct comparison of microarray-generated datasets from different laboratories (Swindell et al., 2014).

Several novel results are highly relevant: the congruence of transcriptomic results from RNAs obtained from different thyroids of individuals of various ages and the clinically independent data on fetal ages validates both types of results and their interpretation (Figure 3A). The thyroid identity is already present in the fetal tissues of the early stages addressed in this study, as demonstrated by the fact that in a PCA performed with all the transcriptomic data (embryonic and adult tissues mix and thyroids), the thyroid tissues are closer to each other than to the mixed tissues from embryonic and adult origin. In addition, the genes composing the first component of this PCA are thyroid specific (Figure 3B). It is also reflected by the fact that many genes are co-regulated in adult and embryonic thyroids compared to other tissues.

Adults and embryonic or fetal thyroids share a lot of regulated genes when compared to other tissues (“mix”): a large number of thyroid related genes were upregulated, and expected genes and transcription factors specific to the thyroid and thyroid function were found (Gao et al., 2019). Nevertheless, some key thyroid-related genes such as TPO, NKX2-1, and DIO1, identified as most highly overexpressed in the ATs, were not found among the most highly upregulated genes in the ETs. Conversely some thyroid-related genes with the strongest fold change (i.e., DUOX2) were not among the highest expressed genes in ATs. These observations indicate that while thyroid-specific profiles of adult and embryonic thyroids are similar, substantial differences between them do exist, presumably due to non-parallel functional differentiation and maturation processes during in the course of thyroid development.

In terms of functions, there is a large overlap of enriched gene ontology (GO) categories in the thyroids versus the mix of tissues, both for adult and fetuses, although some specificity was observed, for instance transport was specifically enriched in ATs. This could mean that, since thyroid function involves the transport of iodine and other ions, as well as proteins (TG), the thyroid is not fully functional in the fetal stages that we have analyzed.

Concerning the overall differences in the transcriptomic profiles of adult and embryonic/fetal tissues, thyroid and non-thyroid developmental signatures indicated that there was a large proportion of co-regulated genes. In other words, the developing thyroid follows the same general trend of human embryonic development.

When excluding from consideration the genes co-regulated in thyroid and non-thyroid tissues during the course of development, the identification of genes specifically regulated in the thyroid belonged to GO functional categories of transport, proliferation, signaling, immune response, development, migration and physiology. Among these large categories, many GO terms were related to Ca^++^ transport. Ca^++^ transport is known to be part of the thyroid signaling pathways (Dumont et al., 1992), and accordingly our data suggest that Ca^++^ transport may play an important role in thyroid development. Among other signaling-related functions, genes specifically related to chemokines, SMAD, and cAMP pathways were identified. The great importance of the cAMP signaling pathway in the function of thyroid is well known. SMAD involvement in thyroid differentiation has been demonstrated (Villacorte et al., 2016). The present results thus support the importance of this latter cascade in thyroid development.

Among functional categories specifically downregulated in the course of thyroid development, retinoic acid-related signaling is known to be an inhibitor of thyroid differentiation.

Interestingly, a very recent work of single cell sequencing has shown for Zebrafish thyrocytes that the population of thyrocytes can be heterogeneous at the transcriptomic level (Gillotay et al., 2020). Of course, our global transcriptomic study does not allow to investigate this.

The study of genes that follow different patterns of expression can also shed light on the differentiation process. Genes specifically involved in thyroid development should demonstrate a pattern of expression different from that of other tissues in the course of development. For instance, we observed that, while thyroid transcription factors (NKX2-1, PAX8, FOXE1, HHEX) remained unchanged in terms of expression in the course of development (adult compared to embryonic thyroids), differentiation markers (TG, TSHR, TPO) showed a continuous increased expression during the course of thyroid development. In pattern I “similar to thyroid specific differentiation factor” (see Figure 8), BMP7 was found, a gene recently pointed to be a key factor for folliculogenesis in mouse (Villacorte et al., 2016) and zebrafish early thyroid development (Haerlingen et al., 2019). The last two patterns (II and IV) are the most interesting, as they could contain genes or signaling effectors which could temporarily replace TSH, which is absent or present in very small quantities while the thyroid becomes functionally active. In the “transient regulator like” (pattern III), we found FGF (FGF10 and FGF 12). FGF and BMP were already identified as key signaling molecules during thyroid differentiation. FGF10 was also reported in mouse thyroid development (Nilsson and Fagman, 2017; Liang et al., 2018): derived from surrounding mesenchyme, it induces branching morphogenesis. IGF1 is also a member of this pattern and is also of great interest: indeed, other receptor activation may contribute to the effects of TSHR or ensure a basal level of thyroid function, despite the fact that the stimulation by these is considerably weaker than with/through the?? TSHR. For example, the expression of TG can be maintained by IGF-1 and other growth factors (Balucan et al., 2013; Dumont et al., 2015). Transgenic mice overexpressing IGF-I and IGF-I receptor in the thyroid gland display low TSH requirement and goiter (Clément et al., 2001). In acromegaly, thyroid pathology has been reported and is correlated to an excess of IGF-1 (Da̧browska et al., 2014; Vitale et al., 2017). A heat map (Figure 4B) illustrates clearly that adult and fetal thyroids are offering a mirror image in terms of expression level of growth factors and receptors.

The thyroid transition state between differentiation (weeks 7–8) and maturation (from weeks 11–12) is objectively demonstrated, and is independent of TSH. To induce such transition, we propose two factors which appear in our thyroid transcriptomic data: FGF and IGF.

Functional differentiation/expansion and maturation of the thyroid takes place at week 10 and later. Genes involved in late thyroid development “maturation” remain unexplored. They were found in the last pattern and we defined them as “maturation factor like*”*: BMP2 was found in this pattern, as DIO1.

The genes identified in this category represent a list of possible necessary factors for thyroid development and maturation, the mutation of which could impair thyroid development, and which could be candidate genes for the genesis of CH.

A strategy independent from prior knowledge of thyroid gene function was to look for known polymorphisms or genetic variants and mutations associated with thyroid function (i.e., variation of thyroid function manifested as alterations in TSH and TH levels in blood), or with thyroid disease in man, or with CH. When confronting our lists of genes (patterns, genes specifically regulated in fetal or AT tissues, or specific to thyroid development) to lists of genes from genome wide studies (SNP and mutations) and also to two transcriptomic datasets comparing thyroid to other tissues, either in adult (Vitale et al., 2017) or in fetus (Gerrard et al., 2016), we found several genes present both in our lists and in the SNP and/or mutation list. Firstly, among genes whose mutations are correlated with CH, IGSF1, involved in development disorders, was present in the list of “transient regulator” (pattern III), meaning that upregulation of this gene in fetal thyroid is no longer found in AT. IGSF1 codes for a member of the immunoglobulin-like domain-containing superfamily, Loss of function mutations of this gene are associated with central CH (Sun et al., 2012), and this gene was recently reported as the most frequently implicated gene in this disease (Joustra et al., 2016). Secondly, another gene whose mutation was recently found in CH is SLC26A7 (Cangul et al., 2019). This gene was found in the first pattern, its expression being strongly upregulated in fetal thyroids compared to fetal tissues mix (fold change: 309) and even more strongly in the ATs (fold change: 792). The protein encoded by this gene is an anion exchanger mediating bicarbonate, chloride, sulfate and oxalate transport. Thirdly, SFTA3, a surfactant protein which is overexpressed both in adult and fetal thyroids, was recently found to be mutated in patients presenting hypothyroidism (Chen et al., 2018). Fourthly, another coregulated gene in our fetal and AT tissues, GLIS3, displays mutations responsible for primary CH and diabetes (Rurale et al., 2018; Scoville et al., 2020). This transcription factor is connected to WNT signaling and in?? primary cilia. Finally IYD (iodotyrosine), an important actor in thyroid hormone synthesis, coregulated in adult and fetal thyroids, but also present in pattern 1, was more expected to be present in this list. A mutation in this gene was first described in 2008 in a CH context, although the relation between CH and IYD defective activity had already been described in 1950. = > ref??

A lot of SNPs have been reported to be associated with TSH and T4 levels and thyroid autoimmune diseases (Medici et al., 2017; Kuś et al., 2020). Genetic factors are known to explain 45–65% of the interindividual variation in TSH and thyroid hormones levels (Hansen et al., 2004). Among the genes that present these variations, we found phosphodiesterases such as PDE8B and PDE10B (Arnaud-Lopez et al., 2008) able to hydrolyze cAMP, and PRKX, a serine/threonine kinase regulated by cAMP and mediating its signaling. The cAMP cascade activation is essential for thyroid differentiation, and in AT, this signaling pathway is activated by TSH binding to its receptor. TSH stimulation induces differentiation in cellular rodent models (Antonica et al., 2012; Carré et al., 2020), in zebrafish (Opitz et al., 2011), pig, dog (Maenhaut et al., 1992; Corvilain et al., 2000), and human (Dumont et al., 1992). In the absence of TSH, as it is the case for our thyroid fetal stages, the participation of these phosphodiesterases could explain partially the onset of thyrocyte functional differentiation that is present. PDE8B is strongly upregulated in fetal thyroids and also in ATs, although to a lesser extent. PDE10A is strongly upregulated in ATs and in the global embryos compared to adults. SNP related with thyroid function variations are also found in B4GALT6. This protein plays a role in the ceramide metabolic pathway, which inhibits cAMP production in TSH stimulated cells (Medici et al., 2017). It is present in “pattern III” of expression, together with PRDM11 whose SNP are reported to be correlated with extreme phenotypes for thyroid function (hyperthyroidism) (Porcu et al., 2013). This gene, encoding for a “PR-domain” protein, a methyltransferase. It play a role in negative regulation of transcription, is highly expressed in the lung, and involved in human cancers. Another interesting gene present in these GWAS analyses is GNAS, upregulated during the course of thyroid development and also in ATs compared to other tissues. GNAS (guanine nucleotide binding protein, alpha stimulating activity polypeptide) causes pseudohypoparathyroidism when mutated (Weinstein et al., 2004). Mutations in GNAS were also found related in toxic thyroid adenomas (Palos-Paz et al., 2008). Signaling through GNAS involves the activation of adenylyl cyclase, resulting in increased levels of cAMP. In case of GNAS hyperfunction, intellectual disability is observed (Freson, 2003).

One limitation of our study is that analyzes have been performed on bulk samples, which does not allow to identify the cell type in which the modulation takes place (follicular cells, C-cells, endothelial cells, fibroblasts…). Future single cell experiments will help to resolve that issue, namely to interpret the function of the proteins regulated in the development phase or later in the maturation phase (e.g., structure, proliferation…).

## Conclusion

Remarkable upregulations of signaling growth factors such as IGF1 and FGF, already found to be involved in animal models of thyroid development, as well proteins involved in the cAMP pathway regulation and activity were highlighted in this transcriptomic analysis of human fetal/embryonic thyroids. This gives clues to explain how this pathway, crucial for the differentiation of thyroid cells, is activated in the absence of TSH during embryonic/fetal development. However, a role of the basal activity of the unstimulated TSHR remains a possibility that cannot be excluded.

A unique advantage of the present study is that it combines staged evolution study of human fetal/embryonic thyroid transcriptomes and its comparison with other tissues transcriptomes at matching stages of development. This allowed us to obtain thyroid specific genes expressed at most stages of development, to investigate how the transcriptomic profiles evolve in the course of thyroid development, and to define which part of this profile is specific to the thyroid. By compiling known functional data with our transcriptomic data, we attempted to increase the power of transcriptomics by adding a more advanced transcriptomic comparison, and strengthen the relevance of discovered genes to thyroid development.

## Data Availability Statement

The datasets presented in this study can be found in online repositories. The names of the repository/repositories and accession number(s) can be found below: https://www.ncbi.nlm.nih.gov/, GSE165706.

## Ethics Statement

The studies involving human participants were reviewed and approved by Erasme Hospital, Université Libre de Bruxelles, and Belgian National Fund for Scientific Research FRS/FNRS. Written informed consent to participate in this study was provided by the participants’ legal guardian/next of kin. Written informed consent was obtained from the individual(s), and minor(s)’ legal guardian/next of kin, for the publication of any potentially identifiable images or data included in this article.

## Author Contributions

CM and JD supervised this research. CM, JD, GD, and PD designed the analysis. GD and PD performed the data analysis and bioinformatic analysis. ML collected and dissected tissue, and took the various measurements of the embryos to assess gestational ages. GD and PD did writing of the original draft. CM, JD, GV, and DG did review and corrected the manuscript. FL and AL performed the RNA seq experiments and analysis. All authors contributed to the article and approved the submitted version.

## Conflict of Interest

The authors declare that the research was conducted in the absence of any commercial or financial relationships that could be construed as a potential conflict of interest.

## Footnotes

1 https://primer3.ut.ee/

2 www.brightcore.be

3 https://degust.erc.monash.edu/

4 http://cbl-gorilla.cs.technion.ac.il/

5 http://bioinformatics.sdstate.edu/idep/

## Supplementary Material

Supplementary Figure 1 Descriptive statistics of intensities of hybridization control probes and quality control metrics used to examine the compatibility of the arrays for the common analysis. **(A–D)** Descriptive statistics of intensities of hybridization control probes “AFFX-r2-Ec-bioB-3_at”; “AFFX-r2-Ec-bioC-3_at”; “AFFX-r2-Ec-bioD-3_at”; “AFFX-r2-P1-cre-3_at” recognizing biotin-labeled cRNA transcripts of bacterial genes bioB, bioC, bioD, and bacteriophage gene cre. As these RNA are spiked into the hybridization cocktail, the intensity of corresponding probes is independent of sample RNA preparation quality. BioB, bioC, bioD, and cre are added in increasing concentrations, BioB quantity corresponding to the technical detection limit of the array scanning system [Affymetrix. GeneChip Expression Analysis. Data Analysis Fundamentals booklet P/N 701190 rev.4]. Each plot corresponds to a separate group of microarrays: **(A)** AT (adult thyroid); **(B)** ET (embryonic thyroid); **(C)** EM (embryonic tissues mix, Yi et al., 2010); **(D)** AM (adult tissue mix, Tateno et al., 2013). **(E–I)** Descriptive statistics of quality control metrics used to evaluate all microarrays used in the study; microarrays were grouped according to the type of sample (AT, ET, EM, or AM). **(E)** % of probes called present. This metric corresponds to the number of probes with intensity that can be reliably detected by the Affymetrix detection algorithm. **(F)** Scaling factors. This metric may be viewed as an estimate of the microarray “brightness” and represents value to which the intensity of every probe on the array has to be multiplied in order to obtain a given, arbitrarily fixed value of the median intensity of the microarray. **(G)** Maximal background intensity. This value expressed in arbitrary units (a.u.) is a measurement of signal intensity caused by autofluorescence of array surface and non-specific binding of Streptavidin-phycoerythrin dye. **(H)** Actin 3′/5′ ratio; **(I)** GAPDH 3′/5′ ratio. These two metrics represent the relative intensity of probes hybridizing to 5′ and 3′ of long transcripts (i.e., GAPDH and beta-actin used as internal control genes). **(J)** Median NUSE; **(K)** Median RLE. These two metrics are explained below. **(L,M)** RLE and NUSE Quality control metrics used to examine the compatibility of the arrays for the common analysis. **(L)** Relative Log Expression boxplots (RLE) show distribution of RLE value for all probes on the array. RLE is calculated for each probe in the array as a difference between its log2 expression on a given microarray and its median log2 expression across all arrays in the set. If the expression of the majority of probes remain unchanged across microarrays in the set, RLE is centered around 0. Significant changes in RLE distribution between arrays in the set may indicate technical bias or a biologically meaningful global changes in gene expression. **(M)** Normalized Unscaled Standard Error (NUSE) boxplots (affyPLM package, Brettschneider et al., 2007) show distribution of NUSE values for each array in the set. Standard error estimates are obtained for each gene on each array using fitting a linear model to probe intensities (PLM fit). As variability differs considerably between genes, the standard error estimates are then normalized so that the median standard error across arrays is 1 for each gene. NUSE plot thus allows to evaluate heterogeneity of probe hybridization on the microarray. Cutoff values are in accordance with Yue et al. (2017).

Supplementary Figure 2 Description of the principles and cutoffs adopted in the analysis of microarrays to obtain the differentially expressed genes. Genes with adjusted *p*-value < 0.05 and at least twofold change were classified as “differentially expressed” (DE, **e,g**). Not differentially expressed (nDE): genes with a local FDR value > 0.95. Genes not fitting into any of these three categories were classified as genes with the uncertain status of differential expression or unmodulated genes (gray area).

Supplementary Figure 3 MA plots and Volcano plots of microarrays data for adults and embryonic thyroids comparison. **(A**) MA plot illustrating comparison 1 (AT vs. ET adult thyroid compared to a mix of embryonic thyroid samples (contrast Adult-SA9-13), all microarray probes are shown. Right: A subset of microarray probes with –1 < log2FC < 1 is shown. **(B)** Volcano plot. Differential expression significance [–log10(*p*-value)] as a function of the log2-transformed fold change of microarray probes. The gradient of the blue color indicates the density of the data points of the scatter plot, dots correspond to outliers. AT, adult thyroids; ET, Embryonic thyroids.

Supplementary Figure 4 Heat maps and clustering. Heatmaps of normalized, background corrected, log2-transformed probe intensities centered by rows. Two-way hierarchical clustering was performed using complete agglomerative clustering method with euclidean distance calculation; each row corresponds to a single gene; each column corresponds to a single sample. For the genes corresponding to multiple probes, only one probe with the lowest adjusted *p*-value is shown. If a probe corresponds to multiple genes, it is shown for each of these genes. **(A)** Hierarchical clustering and heat map with all microarray probes and all samples (ET, weeks 7–9). Heatmap comprising 125 most significant genes differentially expressed in at least one of the four comparisons of biological groups of samples analyzed in the study (1: ET-EM, 2: AT-AM, 3: AT-ET, 4: AM-EM). Non-supervised clustering of genes demonstrate the existence of several groups of which the following are further explored in our study: Gr.A – upregulated in embryos; Gr.B – upregulated in embryonic non-thyroid tissues; Gr.C – specifically upregulated in adult thyroid; Gr.D – upregulated in both in adult and embryonic thyroids; Gr.E – upregulated in adult tissues including thyroid; Gr.F – downregulated both in adult and embryonic thyroids. **(B–E)** Heatmaps comprising 20 most significant differentially expressed genes up- and downregulated in each of the following pairwise comparison of biological groups of samples. **B:** AT-AM, **C:** AM-EM, **D:** ET-EM, and **E:** ET-AT.

Supplementary Figure 5 **(A)** Validation by qRT-PCR of the microarray data. Ratios of gene expression in qRT-PCR and microarray experiments for different embryonic thyroid samples compared to a pool of adult thyroids (Clontech). Samples are labeled as in Supplementary Table 1: GW 7.5: ET2, GW 8(1): ET4, GW 8(2): ET6, GW 9: ET8, GW 10: ET9, GW 11: ET11 adult thyroid samples: BO148N BO147N and CLONT (GW, gestational week). **(B)** Validation by RNAseq of the microarray data for IGSF1, FGF 12, TSHR, DUOX, and SLCA5A. Expression of genes in counts (counts per 20 million of reads) from RNAseq experiments performed on four fetal thyroid samples and on the pool of adult thyroids (Clontech), labeled as in Supplementary Table 1: GW 7.5: ET3, GW 8: ET5, GW 10: ET 10, GW 11: ET11 (GW, gestational week).

Supplementary Figure 6 Graphs showing the correlation between gene expressions in different groups of samples: adult thyroids (AT), embryonic thyroids (ET), embryonic mix (EM), adult mix (AM). **(A)** Genes differentially expressed between ET and EM are positively correlated with genes differentially expressed between AT and AM. **(B)** Genes differentially expressed between AT and ET are positively correlated with genes differentially expressed between AM and EM. **(C)** Genes differentially expressed between AT and ET are not correlated with genes differentially expressed between ET and EM.

Supplementary Figure 7 Strategy used to define genes commonly regulated in the embryonic and adult thyroids or specifically upregulated in the adult or in the embryonic thyroids. **(A)** Subsets of genes differentially expressed in adult thyroids compared to adult tissues mix (comparison 2: AT vs. AM). **(B)** Subsets of genes differentially expressed in embryonic thyroids compared to embryonic tissues mix (comparison 4: ET vs. EM). **(C)** Genes upregulated in adult thyroids compared to adult tissues mix (AT-AM) but not in embryonic thyroids compared to embryonic tissues mix (ET-EM). **(D)** Genes co-upregulated in adult thyroids (AT) and embryonic thyroids (ET) compared to adult tissues mix (AM) and embryonic tissues mix (EM) respectively. **(E)** Genes upregulated in embryonic thyroids compared to embryonic tissues mix (ET-EM) but not in adult thyroids compared to adult tissues mix (AT-AM). Subsets and intersections – **a,a′**: total genes analyzed on the microarray; **b,b′**: total upregulated (adj.*p*-value: any log2FC > 0); **c,c′**: total downregulated (adj.*p*-value: any) (log2FC < 0); **d,d′**: significant and upregulated (adj.*p*-value < 0.05) (log2FC > 0); **e,e′**: differentially expressed (DE) and upregulated (adj.*p*-value < 0.05) log2FC > 1; **f,f′**: not differentially expressed (loc.FDR > 0.95); **g,g′**: differentially expressed (DE) and downregulated: (adj.*p*-value < 0.95) log2FC > 1; **h,h′**: significant and downregulated (adj.*p*-value < 0.05) (log2FC < 0).

Supplementary Figure 8 Strategy used to define genes developmentally regulated in the thyroid and in other tissues in general. **(A)** Subsets of genes differentially expressed in adult thyroids compared to embryonic thyroids (comparison 1: AT vs. ET). **(B)** Subsets of genes differentially expressed in adult tissues mix compared to embryonic tissues mix (comparison 3: AM vs. EM). **(C)** Genes upregulated in adult thyroids (AT) compared to embryonic thyroids (ET) but not in adult tissues mix (AM) compared to embryonic tissues mix (EM). **(D)** Genes downregulated in adult thyroids (AT) compared to embryonic thyroids (ET) but not in adult tissues mix (AM) compared to embryonic tissues mix (EM). Subsets and intersections description – **a,a′:** number of genes analyzed on the microarray; **b,b′:** number of upregulated genes (adj.*p*-value: any log2FC > 0); **c,c′:** number of downregulated genes (adj.*p*-value: any) (log2FC < 0); **d,d′:** significantly upregulated genes (adj.*p*-value < 0.05) (log2FC > 0); e, e′: differentially expressed (DE) and upregulated genes (adj.*p*-value < 0.05) log2FC > 1; **f,f′:** not differentially expressed genes; **g,g′:** differentially expressed (DE) and downregulated: (adj.*p*-value < 0.95) log2FC > 1; **h,h′:** significant and downregulated (adj.*p*-value < 0.05) (log2FC < 0); **i:** genes that are both upregulated in AT compared to ET and down regulated in AM compared to EM; **j:** genes that are both upregulated in AT compared to ET and not regulated in AM compared to EM. **i′**: genes that are both downregulated in AT compared to ET and upregulated in AM compared to EM; **j′:** genes that are both downregulated in AT compared to ET and not regulated in AM compared to EM.
