# Supplementary material for: Transcriptomic Signature of Human Embryonic Thyroid Reveals Transition From Differentiation to Functional Maturation
Source: Front Cell Dev Biol. 2021 Jun 11;9:669354. doi: 10.3389/fcell.2021.669354 (PMC8270686; doi:10.3389/fcell.2021.669354)
Supplement: Supplementary Figure 1 — Descriptive statistics of intensities of hybridization control probes and quality control metrics used to examine the compatibility of the arrays for the common analysis. (A–D) Descriptive statistics of intensities of hybridization control probes “AFFX-r2-Ec-bioB-3_at”; “AFFX-r2-Ec-bioC-3_at”; “AFFX-r2-Ec-bioD-3_at”; “AFFX-r2-P1-cre-3_at” recognizing biotin-labeled cRNA transcripts of bacterial genes bioB, bioC, bioD, and bacteriophage gene cre. As these RNA are spiked into the hybridization cocktail, the intensity of corresponding probes is independent of sample RNA preparation quality. BioB, bioC, bioD, and cre are added in increasing concentrations, BioB quantity corresponding to the technical detection limit of the array scanning system [Affymetrix. GeneChip Expression Analysis. Data Analysis Fundamentals booklet P/N 701190 rev.4]. Each plot corresponds to a separate group of microarrays: (A) AT (adult thyroid); (B) ET (embryonic thyroid); (C) EM (embryonic tissues mix, Yi et al., 2010); (D) AM (adult tissue mix, Tateno et al., 2013). (E–I) Descriptive statistics of quality control metrics used to evaluate all microarrays used in the study; microarrays were grouped according to the type of sample (AT, ET, EM, or AM). (E) % of probes called present. This metric corresponds to the number of probes with intensity that can be reliably detected by the Affymetrix detection algorithm. (F) Scaling factors. This metric may be viewed as an estimate of the microarray “brightness” and represents value to which the intensity of every probe on the array has to be multiplied in order to obtain a given, arbitrarily fixed value of the median intensity of the microarray. (G) Maximal background intensity. This value expressed in arbitrary units (a.u.) is a measurement of signal intensity caused by autofluorescence of array surface and non-specific binding of Streptavidin-phycoerythrin dye. (H) Actin 3′/5′ ratio; (I) GAPDH 3′/5′ ratio. These two metrics represent the relative inten [file Presentation_1.PPTX]

## Slide 1
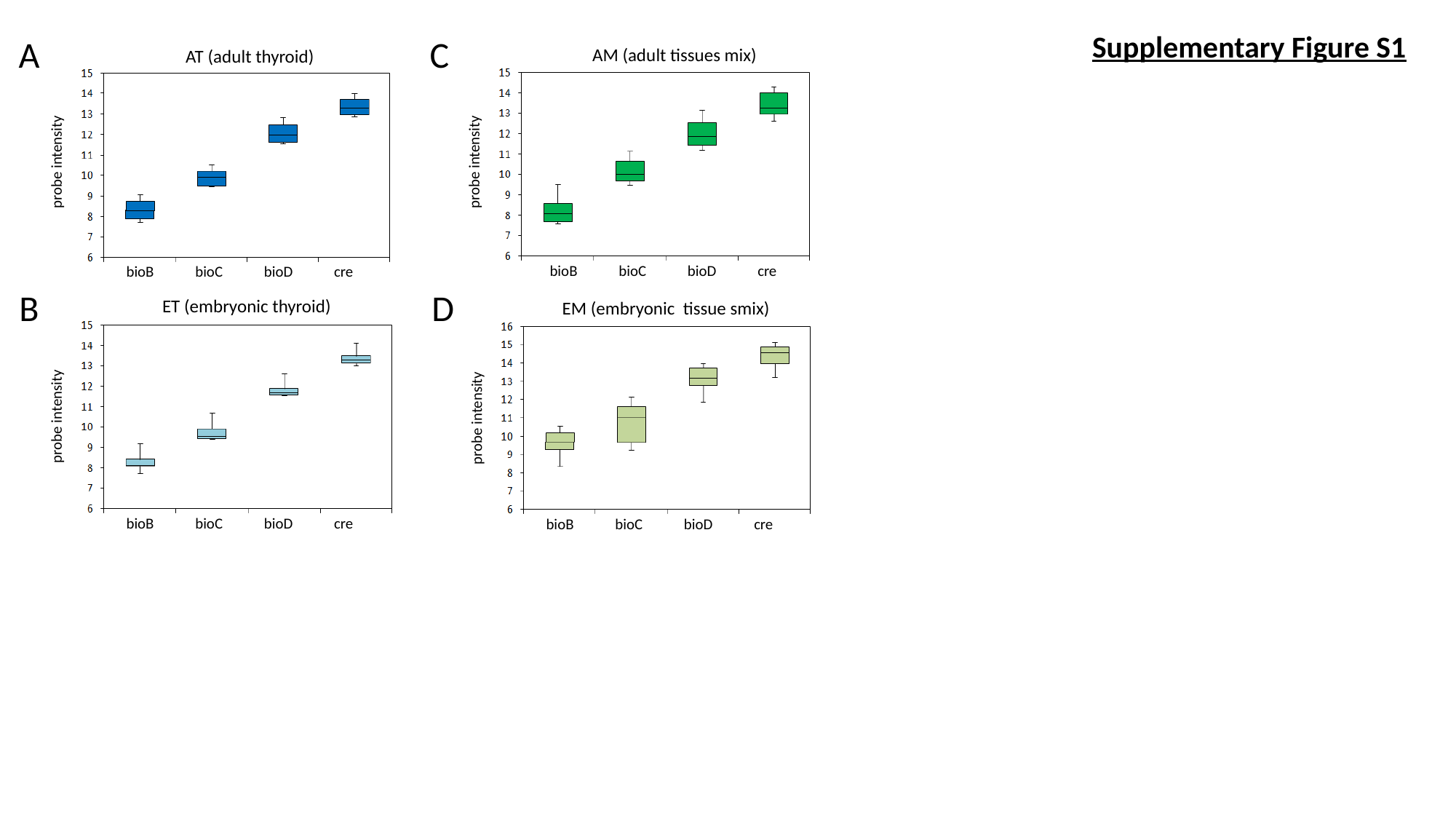

Supplementary Figure S1
A
C
AM (adult tissues mix)
AT (adult thyroid)
probe intensity
probe intensity
bioB bioC bioD cre
bioB bioC bioD cre
B
D
ET (embryonic thyroid)
EM (embryonic tissue smix)
probe intensity
probe intensity
bioB bioC bioD cre
bioB bioC bioD cre

## Slide 2
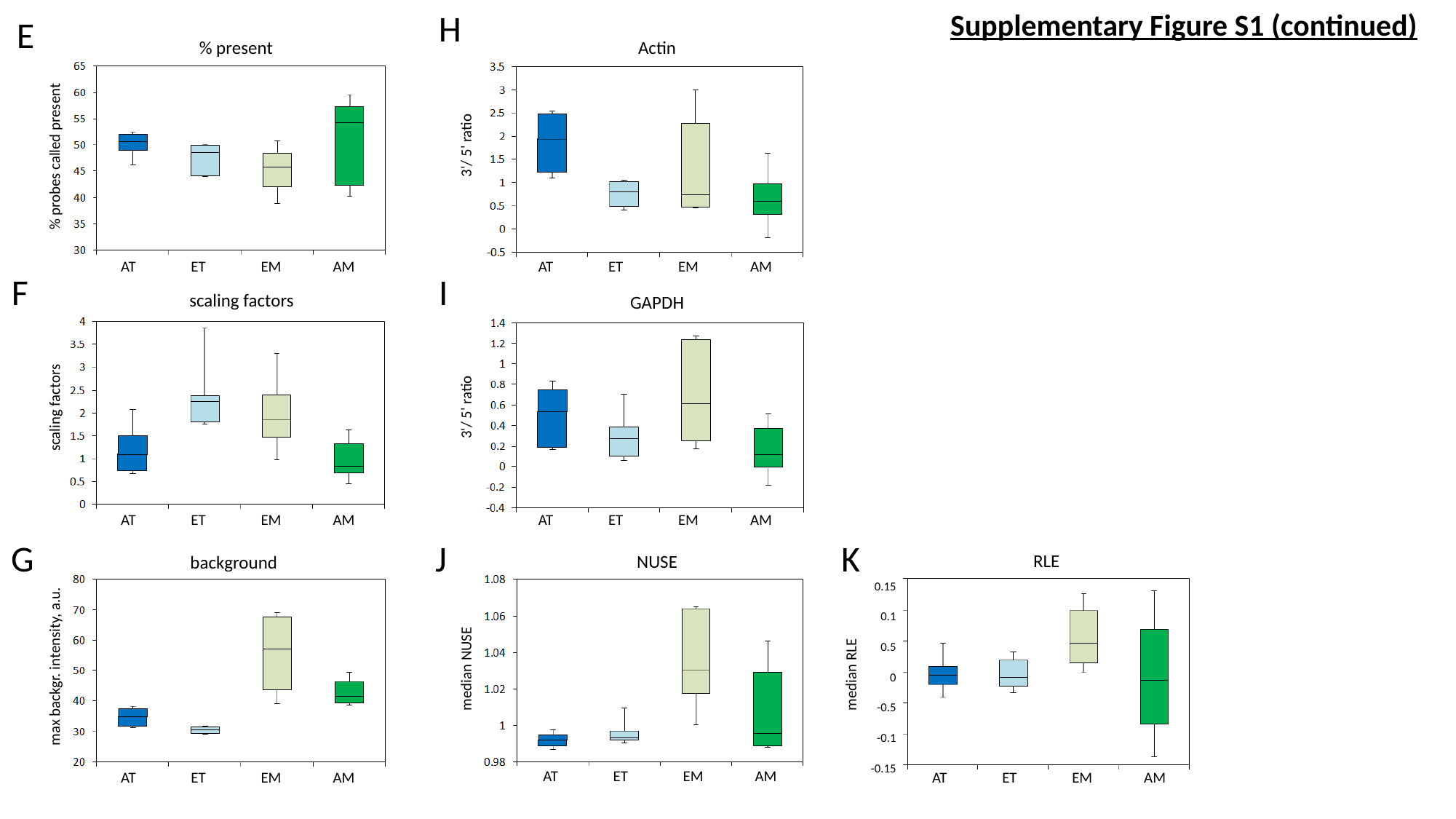

H
Supplementary Figure S1 (continued)
E
% present
Actin
3'/ 5' ratio
% probes called present
AT ET EM AM
AT ET EM AM
F
I
scaling factors
GAPDH
3'/ 5' ratio
scaling factors
AT ET EM AM
AT ET EM AM
G
J
K
RLE
NUSE
background
0.15
0.1
0.5
0
-0.5
-0.1
-0.15
max backgr. intensity, a.u.
median NUSE
median RLE
AT ET EM AM
AT ET EM AM
AT ET EM AM

## Slide 3
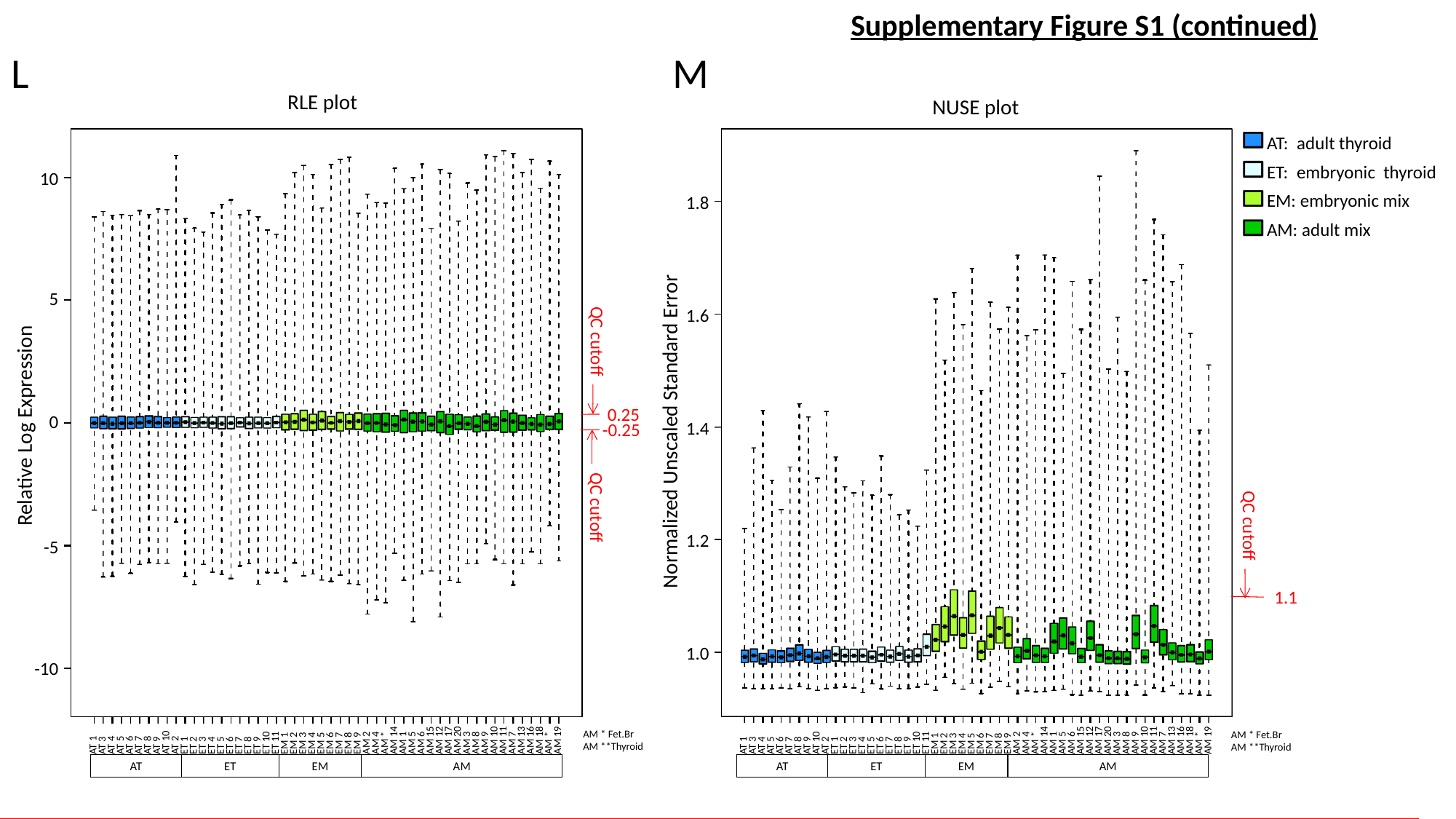

Supplementary Figure S1 (continued)
L
M
RLE plot
NUSE plot
AT: adult thyroid
ET: embryonic thyroid
10
5
0
-5
-10
1.8
1.6
1.4
1.2
1.0
EM: embryonic mix
-
AM: adult mix
-
-
-
-
QC cutoff
0.25
Relative Log Expression
-0.25
Normalized Unscaled Standard Error
QC cutoff
AT 1
AT 3
AT 4
AT 5
AT 6
AT 7
AT 8
AT 9
AT 10
AT 2
ET 1
ET 2
ET 3
ET 4
ET 5
ET 6
ET 7
ET 8
ET 9
ET 10
ET 11
EM 1
EM 2
EM 3
EM 4
EM 5
EM 6
EM 7
EM 8
EM 9
AM 2
AM 4
AM *
AM 14
AM 1
AM 5
AM 6
AM 15
AM 12
AM 17
AM 20
AM 3
AM 8
AM 9
AM 10
AM 11
AM 7
AM 13
AM 16
AM 18
AM *
AM 19
AT 1
AT 3
AT 4
AT 5
AT 6
AT 7
AT 8
AT 9
AT 10
AT 2
ET 1
ET 2
ET 3
ET 4
ET 5
ET 6
ET 7
ET 8
ET 9
ET 10
ET 11
EM 1
EM 2
EM 3
EM 4
EM 5
EM 6
EM 7
EM 8
EM 9
AM 2
AM 4
AM *
AM 14
AM 1
AM 5
AM 6
AM 15
AM 12
AM 17
AM 20
AM 3
AM 8
AM 9
AM 10
AM 11
AM 7
AM 13
AM 16
AM 18
AM *
AM 19
QC cutoff
1.1
AM * Fet.Br
AM **Thyroid
AM * Fet.Br
AM **Thyroid
AT
ET
EM
AM
AT
ET
EM
AM

## Slide 4
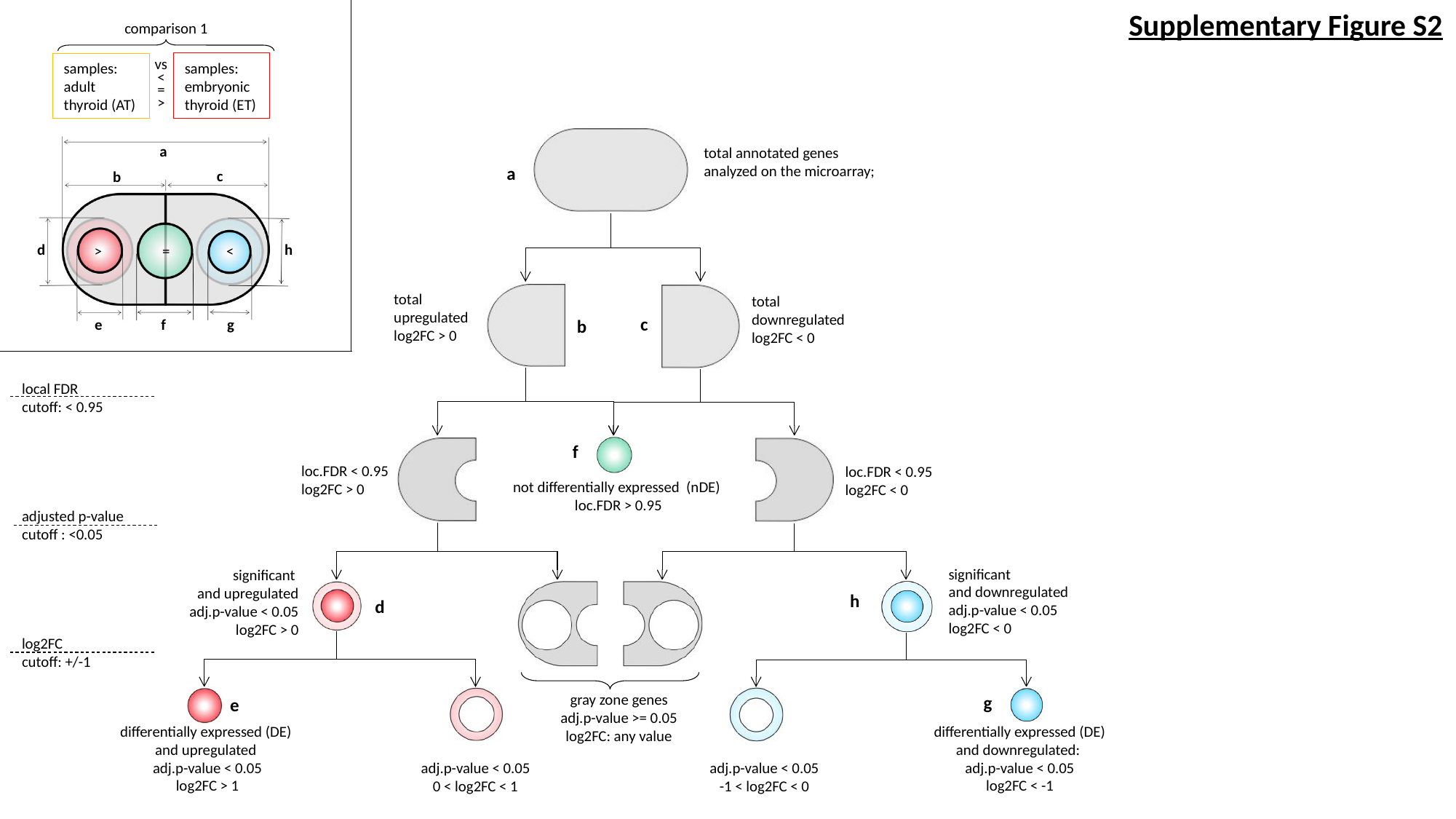

Supplementary Figure S2
comparison 1
samples:
embryonic
thyroid (ET)
samples:
adult
thyroid (AT)
vs
<
=
>
a
total annotated genes analyzed on the microarray;
a
c
b
b
d
h
>
=
<
total
upregulated
log2FC > 0
total
downregulated
log2FC < 0
c
b
e
f
g
local FDR
cutoff: < 0.95
f
loc.FDR < 0.95
log2FC > 0
loc.FDR < 0.95
log2FC < 0
not differentially expressed (nDE)
loc.FDR > 0.95
adjusted p-value
cutoff : <0.05
significant
and downregulated
adj.p-value < 0.05
log2FC < 0
significant
and upregulated
adj.p-value < 0.05
log2FC > 0
h
d
log2FC
cutoff: +/-1
gray zone genes
adj.p-value >= 0.05
log2FC: any value
g
e
differentially expressed (DE)
and upregulated
adj.p-value < 0.05
log2FC > 1
differentially expressed (DE)
and downregulated:
adj.p-value < 0.05
log2FC < -1
adj.p-value < 0.05
0 < log2FC < 1
adj.p-value < 0.05
-1 < log2FC < 0

## Slide 5
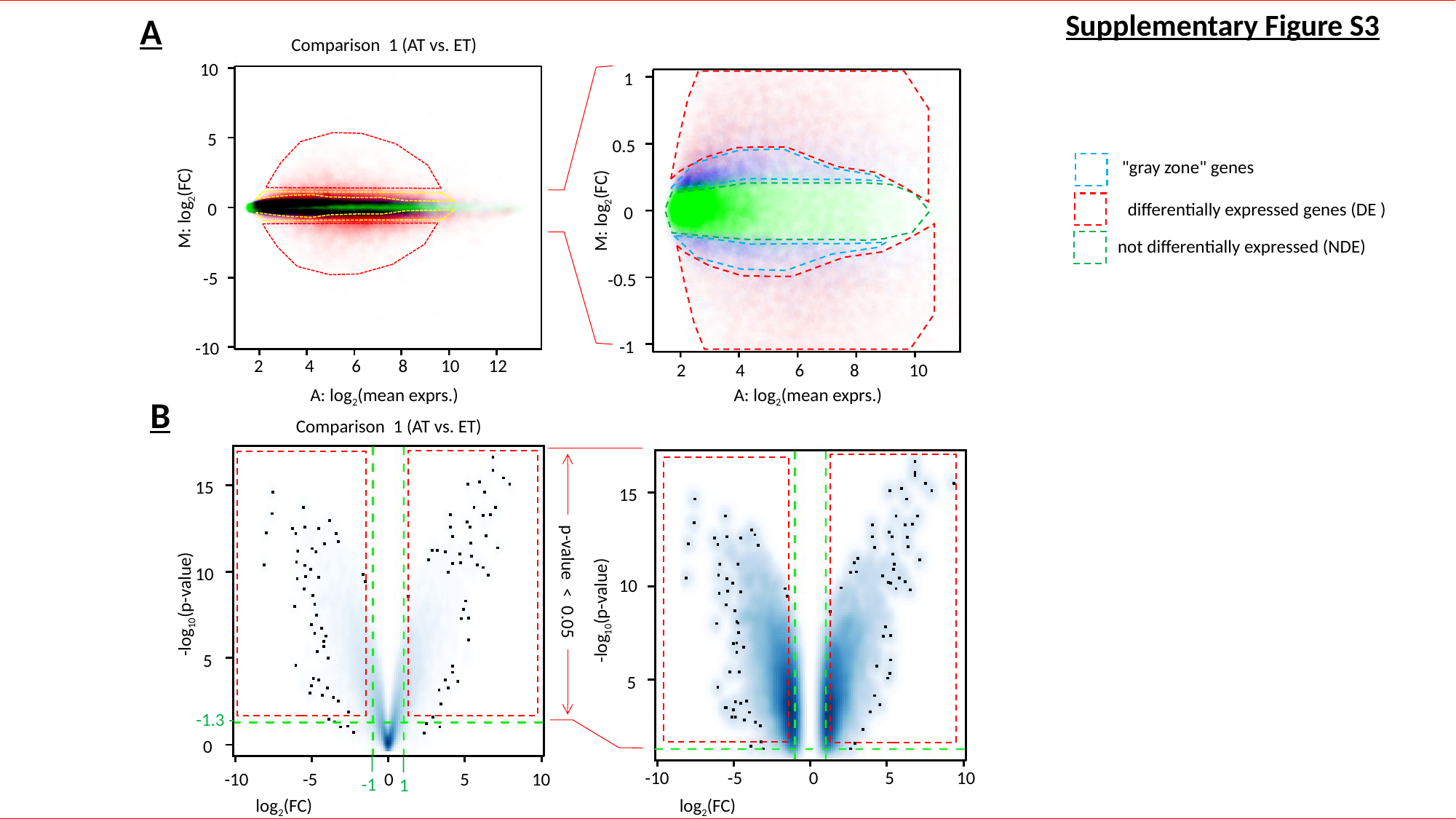

C
Supplementary Figure S3
A
Comparison 1 (AT vs. ET)
10
5
0
-5
-10
1
0.5
0
-0.5
-1
"gray zone" genes
M: log2(FC)
differentially expressed genes (DE )
M: log2(FC)
not differentially expressed (NDE)
2 4 6 8 10 12
2 4 6 8 10
A: log2(mean exprs.)
A: log2(mean exprs.)
B
Comparison 1 (AT vs. ET)
15
10
5
0
15
10
5
p-value < 0.05
-log10(p-value)
-log10(p-value)
-1.3 -
|
-1
|
1
-10 -5 0 5 10
-10 -5 0 5 10
log2(FC)
log2(FC)

## Slide 6
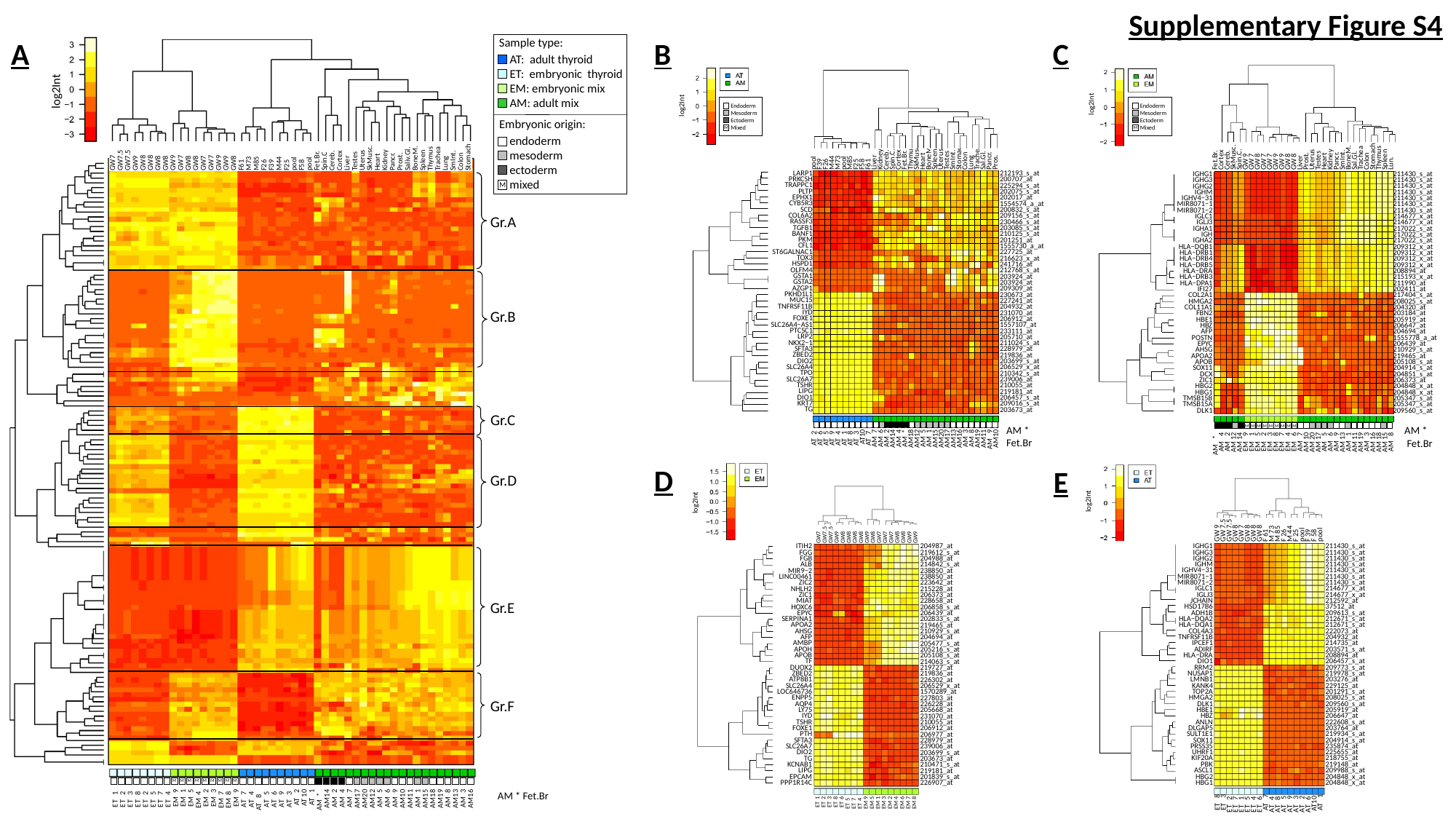

Supplementary Figure S4
A
C
Sample type:
B
GW7
GW7.5
GW7.5
GW9
GW8
GW8
GW8
GW8
GW9
GW7
GW8
GW8
GW7
GW7
GW9
GW9
GW8
F61
M73
M85
F26
F39
M44
F25
pool
F58
pool
Fet.Br.
Spin.C
Cereb.
Cortex
Liver
Testes
Uterus
SkMusc.
Heart
Kidney
Pancr.
Prost.
Saliv.Gl.
BoneM.
Spleen
Thymus
Trachea
Lung
SmInt.
Colon
Stomach
log2Int
Gr.A
Gr.B
Gr.C
Gr.D
Gr.E
ET 1
ET 2
ET 3
ET 8
ET 2
ET 5
ET 7
ET 4
EM 9
EM 1
EM 5
EM 4
EM 2
EM 3
EM 7
EM 8
EM 9
AT 7
AT 4
AT 8
AT 5
AT 6
AT 9
AT 3
AT 2
AT 10
AT 1
AM *
AM14
AM 2
AM 4
AM 7
AM17
AM20
AM12
AM 5
AM 6
AM 9
AM10
AM11
AM 1
AM15
AM18
AM19
AM 8
AM13
AM 3
AM16
Gr.F
M
M
M
M
M
M
M
M
M
AM * Fet.Br
AT: adult thyroid
ET: embryonic thyroid
EM: embryonic mix
AM: adult mix
pool
F39
F26
M44
M73
pool
M85
F25
F58
F61
Liver
Kidney
Cereb.
Spin.C.
Cortex
Fet.Br.
Thymus
SkMusc.
Heart
BoneM.
Spleen
Uterus
Testes
SmInt.
Stomach
Colon
Lung
Trachea
Sal.Gl.
Pancr.
Pros.
Fet.Br.
Cortex
Cereb.
SkMusc.
Spin.C.
GW 9
GW 7
GW 8
GW 7
GW 7
GW 9
GW 9
GW 8
GW 8
Liver
Prost.
Uterus
Testes
Heart
Kidney
Pancr.
SmInt.
BoneM.
Sal.Gl.
Trachea
Colon
Stomach
Thymus
Spleen
Lun.
log2Int
log2Int
Endoderm
Mesoderm
Ectoderm
Mixed
Endoderm
Mesoderm
Ectoderm
Mixed
Embryonic origin:
M
M
endoderm
mesoderm
ectoderm
mixed
LARP1
PRKCSH
TRAPPC1
PLTP
EPHX1
CYB5R3
SCD
COL6A2
RASSF3
TGFB1
BANF1
PKM
CFL1
ST6GALNAC1
TOX3
HSPD1
OLFM4
GSTA1
GSTA2
AZGP1
PKHD1L1
MUC15
TNFRSF11B
IYD
FOXE1
SLC26A4−AS1
PTCSC1
LRP2
NKX2−1
SFTA3
ZBED2
DIO2
SLC26A4
TPO
SLC26A7
TSHR
LIPG
DIO1
KRT7
TG
212193_s_at
200707_at
225294_s_at
202075_s_at
202017_at
1554574_a_at
200832_s_at
209156_s_at
230466_s_at
203085_s_at
210125_s_at
201251_at
1555730_a_at
227725_at
216623_x_at
241716_at
212768_s_at
203924_at
203924_at
209309_at
230673_at
227241_at
204932_at
231070_at
206912_at
1557107_at
233111_at
205710_at
211024_s_at
228979_at
219836_at
203699_s_at
206529_x_at
210342_s_at
239006_at
210055_at
219181_at
206457_s_at
209016_s_at
203673_at
211430_s_at
211430_s_at
211430_s_at
211430_s_at
211430_s_at
211430_s_at
211430_s_at
214677_x_at
214677_x_at
217022_s_at
217022_s_at
217022_s_at
209312_x_at
209312_x_at
209312_x_at
209312_x_at
208894_at
215193_x_at
211990_at
202411_at
217404_s_at
208025_s_at
204320_at
203184_at
205919_at
206647_at
204694_at
1555778_a_at
206439_at
210929_s_at
219465_at
205108_s_at
204914_s_at
204851_s_at
206373_at
204848_x_at
204848_x_at
205347_s_at
205347_s_at
209560_s_at
IGHG1
IGHG3
IGHG2
IGHM
IGHV4−31
MIR8071−1
MIR8071−2
IGLC1
IGLJ3
IGHA1
IGH
IGHA2
HLA−DQB1
HLA−DRB1
HLA−DRB4
HLA−DRB5
HLA−DRA
HLA−DRB3
HLA−DPA1
IFI27
COL2A1
HMGA2
COL11A1
FBN2
HBE1
HBZ
AFP
POSTN
EPYC
AHSG
APOA2
APOB
SOX11
DCX
ZIC1
HBG2
HBG1
TMSB15B
TMSB15A
DLK1
M
AT 2
AT 6
AT 5
AT 9
AT 4
AT 1
AT 8
AT 3
AT10
AT 7
AM 7
AM 6
AM 2
AM14
AM 4
AM *
AM18
AM12
AM 5
AM 1
AM15
AM20
AM17
AM13
AM16
AM 3
AM 8
AM19
AM11
AM 9
AM10
AM *
AM 4
AM 2
AM 12
AM 14
EM 9
EM 1
EM 5
EM 2
EM 3
EM 8
EM 7
EM 4
EM 6
AM 7
AM 10
AM 20
AM 17
AM 5
AM 6
AM 9
AM 13
AM 1
AM 11
AM 19
AM 3
AM 16
AM 18
AM 15
AM 8
AM *
Fet.Br
AM *
 Fet.Br
M
M
M
M
M
M
M
M
M
D
E
GW 9
GW 7.5
GW 7.5
GW 8
GW 7
GW 8
GW 8
GW 8
F 61
M 73
M 85
F 26
M 44
F 25
pool
F 39
F 58
pool
GW7
GW7.5
GW7.5
GW9
GW8
GW8
GW8
GW8
GW9
GW8
GW7
GW7
GW7
GW8
GW8
GW9
GW9
log2Int
log2Int
211430_s_at
211430_s_at
211430_s_at
211430_s_at
211430_s_at
211430_s_at
211430_s_at
214677_x_at
214677_x_at
212592_at
37512_at
209613_s_at
212671_s_at
212671_s_at
222073_at
204932_at
214735_at
203571_s_at
208894_at
206457_s_at
209773_s_at
219978_s_at
203276_at
229125_at
201291_s_at
208025_s_at
209560_s_at
205919_at
206647_at
222608_s_at
203764_at
219934_s_at
204914_s_at
235874_at
225655_at
218755_at
219148_at
209988_s_at
204848_x_at
204848_x_at
IGHG1
IGHG3
IGHG2
IGHM
IGHV4−31
MIR8071−1
MIR8071−2
IGLC1
IGLJ3
JCHAIN
HSD17B6
ADH1B
HLA−DQA2
HLA−DQA1
COL4A3
TNFRSF11B
IPCEF1
ADIRF
HLA−DRA
DIO1
RRM2
NUSAP1
LMNB1
KANK4
TOP2A
HMGA2
DLK1
HBE1
HBZ
ANLN
DLGAP5
SULT1E1
SOX11
PRSS35
UHRF1
KIF20A
PBK
ASCL1
HBG2
HBG1
ITIH2
FGG
FGB
ALB
MIR9−2
LINC00461
ZIC2
NHLH2
ZIC1
MIAT
HOXC6
EPYC
SERPINA1
APOA2
AHSG
AFP
AMBP
APOH
APOB
TF
DUOX2
ZBED2
ATP8B1
SLC26A4
LOC646736
ENPP5
AQP4
LY75
IYD
TSHR
FOXE1
PTH
SFTA3
SLC26A7
DIO2
TG
KCNAB1
LIPG
EPCAM
PPP1R14C
204987_at
219612_s_at
204988_at
214842_s_at
238850_at
238850_at
223642_at
215228_at
206373_at
228658_at
206858_s_at
206439_at
202833_s_at
219465_at
210929_s_at
204694_at
205477_s_at
205216_s_at
205108_s_at
214063_s_at
219727_at
219836_at
226302_at
206529_x_at
1570289_at
227803_at
226228_at
205668_at
231070_at
210055_at
206912_at
206977_at
228979_at
239006_at
203699_s_at
203673_at
210471_s_at
219181_at
201839_s_at
226907_at
ET 8
ET 3
ET 2
ET 7
ET 1
ET 5
ET 4
ET 6
AT 7
AT 4
AT 8
AT 5
AT 9
AT 3
AT 2
AT 6
AT10
AT 1
ET 1
ET 2
ET 3
ET 8
ET 6
ET 5
ET 7
ET 4
EM 9
EM 5
EM 1
EM 3
EM 2
EM 4
EM 6
EM 7
EM 8

## Slide 7
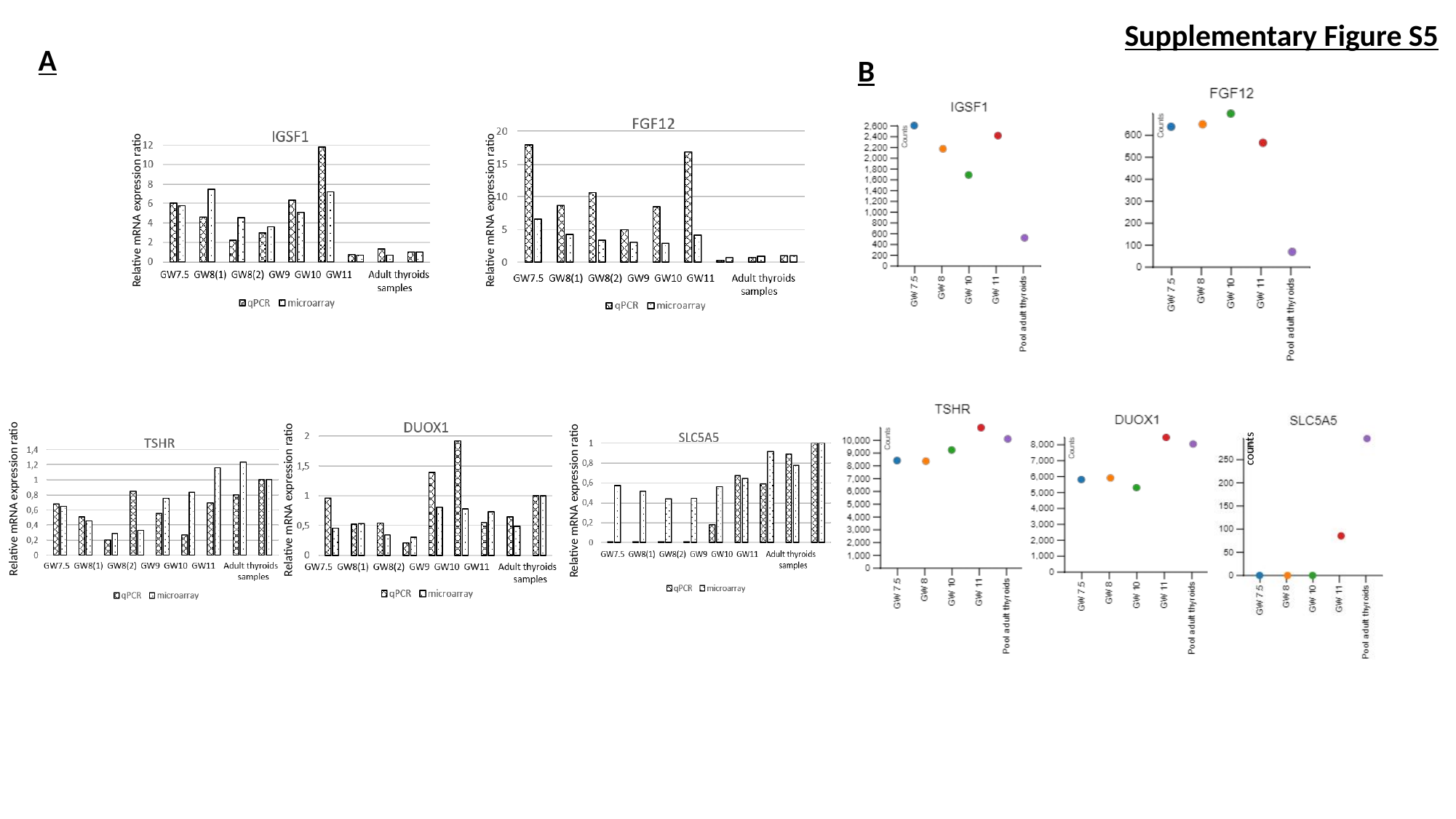

Supplementary Figure S5
A
B
Relative mRNA expression ratio
Relative mRNA expression ratio
counts
Relative mRNA expression ratio
Relative mRNA expression ratio
Relative mRNA expression ratio

## Slide 8
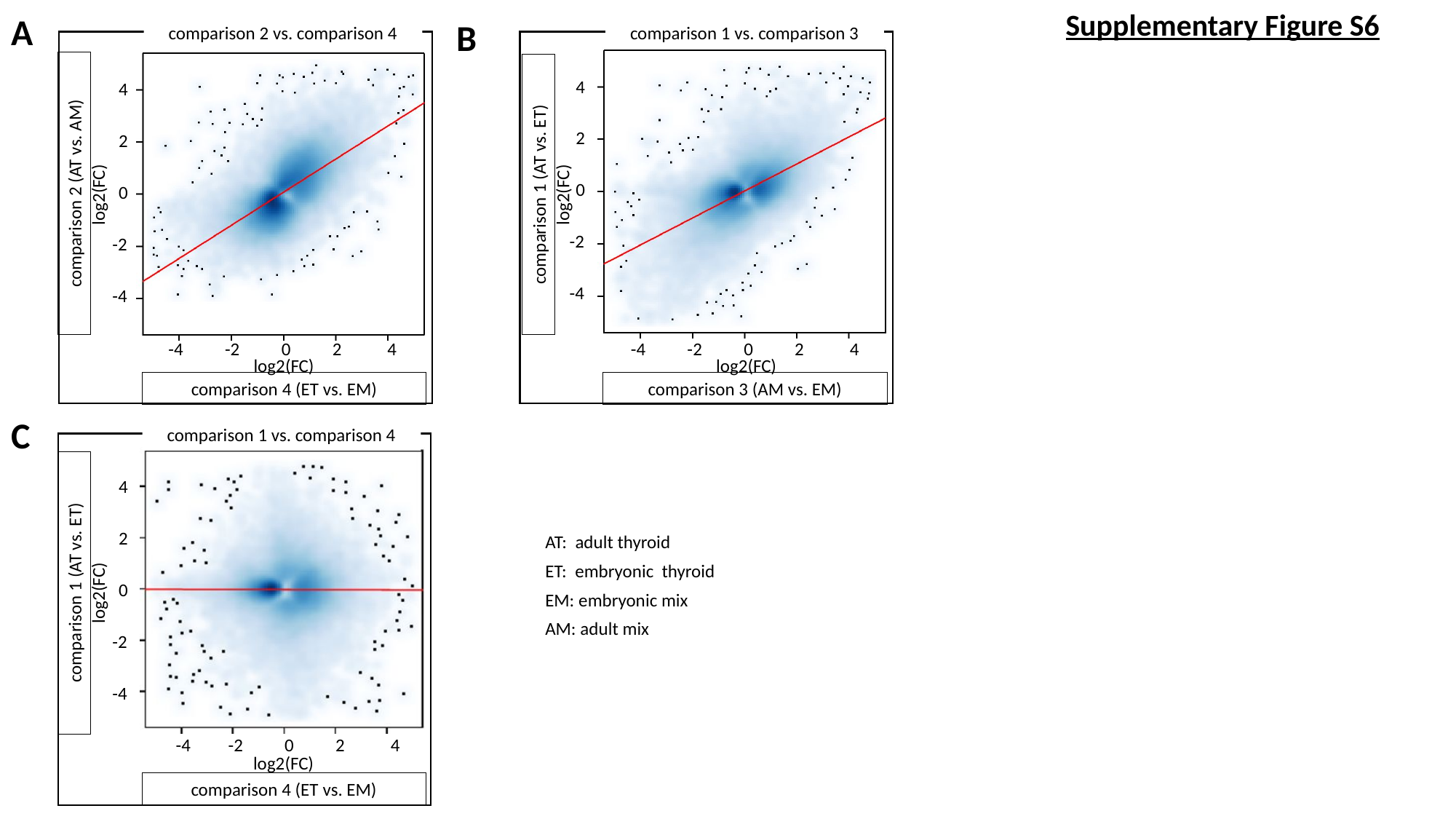

Supplementary Figure S6
A
B
comparison 2 vs. comparison 4
comparison 1 vs. comparison 3
4
2
0
-2
-4
4
2
0
-2
-4
log2(FC)
log2(FC)
comparison 2 (AT vs. AM)
comparison 1 (AT vs. ET)
-4 -2 0 2 4
-4 -2 0 2 4
log2(FC)
log2(FC)
comparison 4 (ET vs. EM)
comparison 3 (AM vs. EM)
C
comparison 1 vs. comparison 4
4
2
0
-2
-4
AT: adult thyroid
ET: embryonic thyroid
log2(FC)
comparison 1 (AT vs. ET)
EM: embryonic mix
AM: adult mix
-4 -2 0 2 4
log2(FC)
comparison 4 (ET vs. EM)

## Slide 9
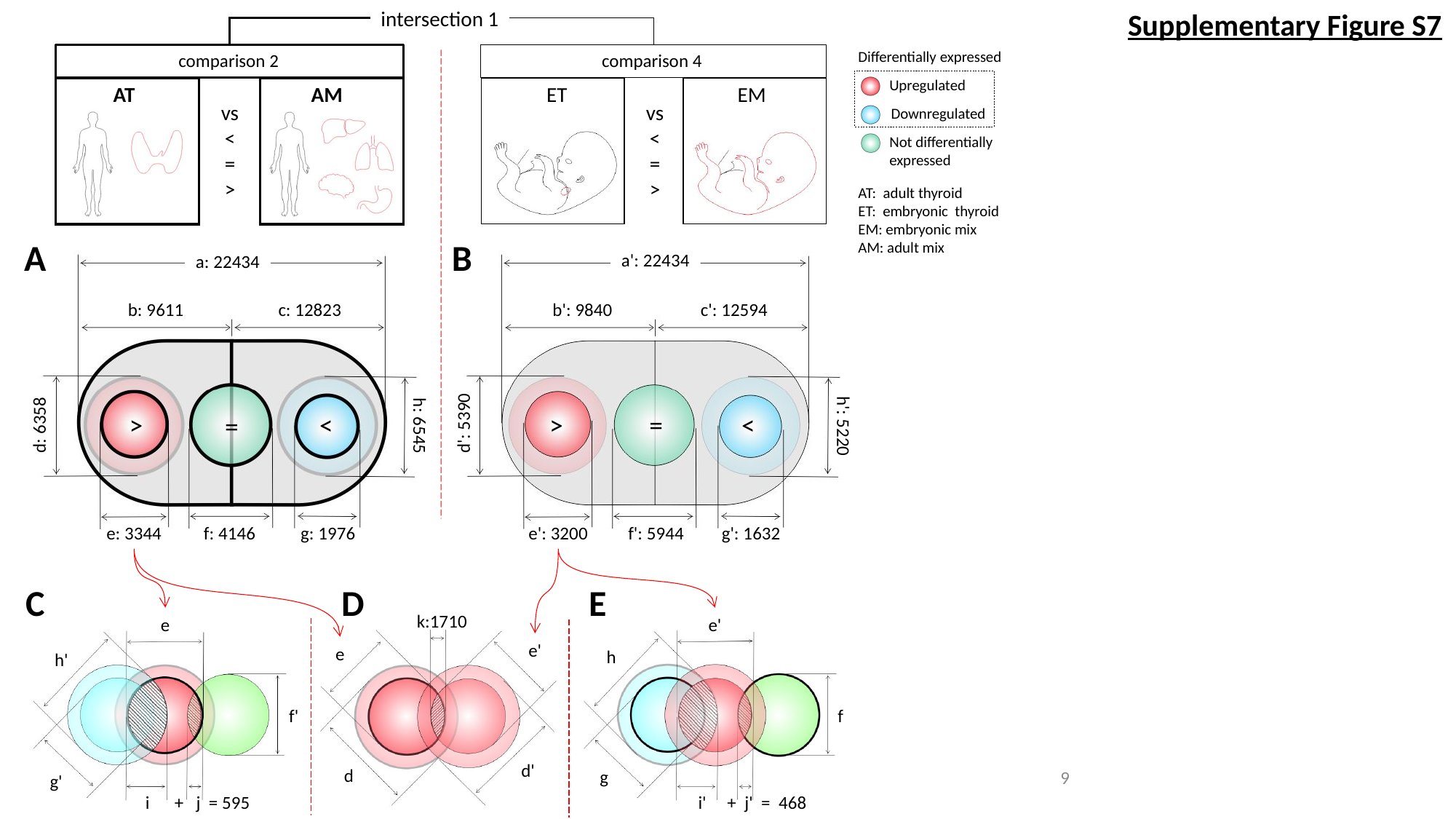

intersection 1
Supplementary Figure S7
Differentially expressed
comparison 2
comparison 4
Upregulated
AT
AM
ET
EM
vs
<
=
>
vs
<
=
>
Downregulated
Not differentially
expressed
AT: adult thyroid
ET: embryonic thyroid
EM: embryonic mix
AM: adult mix
A
B
a': 22434
a: 22434
b: 9611
c: 12823
b': 9840
c': 12594
>
<
>
=
<
=
d': 5390
d: 6358
h: 6545
h': 5220
e: 3344
f: 4146
g: 1976
e': 3200
f': 5944
g': 1632
C
D
E
k:1710
e
e'
e'
e
h
h'
f
f'
d'
9
d
g
g'
i + j = 595
i' + j' = 468

## Slide 10
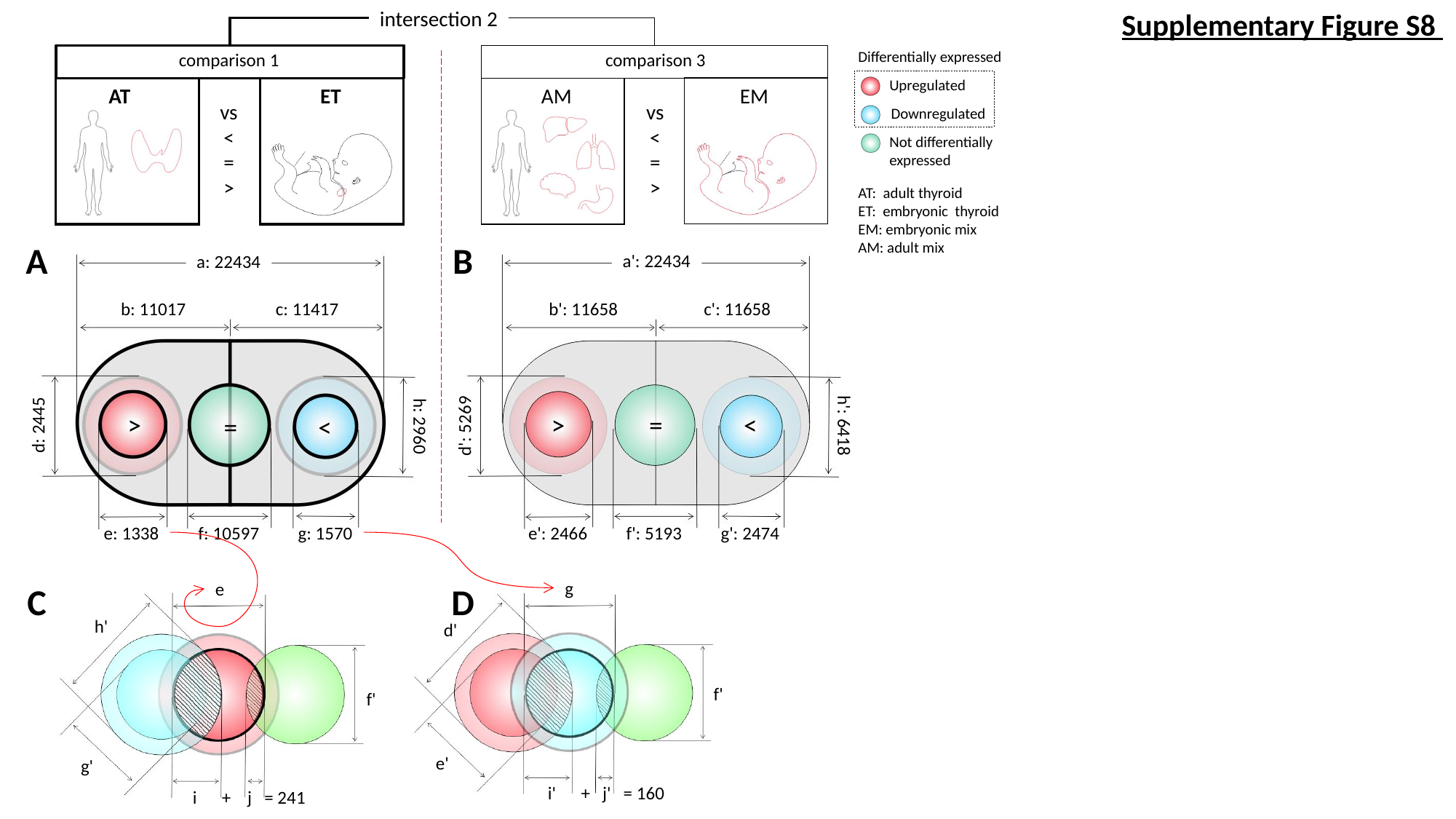

intersection 2
Supplementary Figure S8
Differentially expressed
comparison 1
comparison 3
Upregulated
AT
ET
AM
EM
vs
<
=
>
vs
<
=
>
Downregulated
Not differentially
expressed
AT: adult thyroid
ET: embryonic thyroid
EM: embryonic mix
AM: adult mix
A
B
a': 22434
a: 22434
b: 11017
c: 11417
b': 11658
c': 11658
=
>
<
>
=
<
d: 2445
d': 5269
h': 6418
h: 2960
e: 1338
f: 10597
g: 1570
e': 2466
f': 5193
g': 2474
g
e
C
D
h'
d'
f'
f'
e'
g'
i' + j' = 160
i + j = 241
