# Supplementary material for: Transcriptomic Signature of Human Embryonic Thyroid Reveals Transition From Differentiation to Functional Maturation
Source: Front Cell Dev Biol. 2021 Jun 11;9:669354. doi: 10.3389/fcell.2021.669354 (PMC8270686; doi:10.3389/fcell.2021.669354)
Supplement: Supplementary file 6 [file Table_5.docx]

# Supplementary table 5:

# Comparisons 1-4

## Description

Supplementary file 1

tables of top 20 genes differentially expressed in various comparisons

protein -coding,

comparison 1. Adult thyroid (AT) vs. embryonic thyroid (ET)

comparison 2. Adult thyroid (AT) vs. adult tissue mix (AM)

comparison 3. Adult tissue mix (AM) vs. embryonic tissue mix (EM)

comparison 4. Embryonic thyroid (ET) vs. embryonic tissue mix (EM)

supplementary file top20 diffentially expressed protein coding genes

Contents

[Supplementary file 1 Contrasts 1](#_Toc62801369)

[Description 1](#_Toc62801370)

[1.1. "AT vs. ET" 2](#_Toc62801371)

[1.2. "AT vs. AM" 3](#_Toc62801372)

[1.3. "AM vs. EM" 4](#_Toc62801373)

[1.4. "ET vs. EM" 5](#_Toc62801374)

### 1.1. "AT vs. ET"

Table 1.1.A. "AT vs. ET" (AT>ET).

Top 20 protein-coding genes differentially expressed in the adult thyroid (AT) as compared to the embryonic thyroid (ET) (comparison 1) and demonstrating a higher expression in the adult thyroid (AT>ET).

| **gene symbol** |  | **logFC** | **adj.P.Val** | **best probe** | **gene description** |
| --- | --- | --- | --- | --- | --- |
| TNFRSF11B | protein coding | 6.497558 | 1.17E-19 | 204932_at | TNF receptor superfamily member 11b |
| ADH1B | protein coding | 6.052636 | 4.63E-06 | 209613_s_at | alcohol dehydrogenase 1B (class I), beta polypeptide |
| ADIRF | protein coding | 6.028724 | 2.94E-12 | 203571_s_at | adipogenesis regulatory factor |
| HSD17B6 | protein coding | 5.86443 | 9.96E-07 | 37512_at | hydroxysteroid 17-beta dehydrogenase 6 |
| HLA-DRA | protein coding | 5.598742 | 3.85E-10 | 208894_at | major histocompatibility complex, class II, DR alpha |
| DIO1 | protein coding | 5.532955 | 1.81E-06 | 206457_s_at | iodothyronine deiodinase 1 |
| JCHAIN | protein coding | 5.440888 | 0.000528 | 212592_at | joining chain of multimeric IgA and IgM |
| IPCEF1 | protein coding | 5.326228 | 9.41E-11 | 214735_at | interaction protein for cytohesin exchange factors 1 |
| HLA-DQA1,2 | protein coding | 5.216224 | 5.14E-07 | 212671_s_at | major histocompatibility complex, class II, DQ alpha 1,2 |
| COL4A3 | protein coding | 5.166449 | 8.95E-09 | 222073_at | collagen type IV alpha 3 chain |
| MT1M | protein coding | 5.13314 | 5.88E-07 | 217546_at | metallothionein 1M |
| TPO | protein coding | 5.091257 | 2.91E-16 | 210342_s_at | thyroid peroxidase |
| IFI27 | protein coding | 5.000299 | 8.47E-13 | 202411_at | interferon alpha inducible protein 27 |
| EGR2 | protein coding | 4.775596 | 4.78E-11 | 205249_at | early growth response 2 |
| PKHD1L1 | protein coding | 4.73642 | 2.25E-12 | 230673_at | PKHD1 like 1 |
| SUCNR1 | protein coding | 4.69881 | 1.10E-09 | 223939_at | succinate receptor 1 |
| TCERG1L | protein coding | 4.6634 | 8.43E-12 | 231257_at | transcription elongation regulator 1 like |
| RGS1 | protein coding | 4.633268 | 2.48E-06 | 216834_at | regulator of G protein signaling 1 |
| ANXA1 | protein coding | 4.561993 | 5.39E-13 | 233011_at | annexin A1 |
| CLIC2 | protein coding | 4.542345 | 1.99E-09 | 213415_at | chloride intracellular channel 2 |

Table 1.1.B. "AT vs. ET (ET>AT)"

Top 20 protein-coding genes differentially expressed in the adult thyroid (AT) as compared to the embryonic thyroid (ET) (comparison 1) and demonstrating a higher expression in the embryonic thyroid (ET>AT).

| **gene symbol** |  | **logFC** | **adj.P.Val** | **best probe** | **gene description** |
| --- | --- | --- | --- | --- | --- |
| CDCA7 | protein coding | -5.21324 | 4.70E-08 | 224428_s_at | cell division cycle associated 7 |
| KANK4 | protein coding | -5.22921 | 6.32E-12 | 229125_at | KN motif and ankyrin repeat domains 4 |
| NUSAP1 | protein coding | -5.31162 | 1.36E-10 | 219978_s_at | nucleolar and spindle associated protein 1 |
| RRM2 | protein coding | -5.36686 | 5.16E-07 | 209773_s_at | ribonucleotide reductase regulatory subunit M2 |
| ASCL1 | protein coding | -5.4748 | 1.20E-06 | 209988_s_at | achaete-scute family bHLH transcription factor 1 |
| SULT1E1 | protein coding | -5.61713 | 4.01E-15 | 219934_s_at | sulfotransferase family 1E member 1 |
| PBK | protein coding | -5.69302 | 2.52E-07 | 219148_at | PDZ binding kinase |
| HBZ | protein coding | -5.75376 | 1.66E-12 | 206647_at | hemoglobin subunit zeta |
| KIF20A | protein coding | -5.75984 | 9.28E-12 | 218755_at | kinesin family member 20A |
| UHRF1 | protein coding | -5.80299 | 9.21E-11 | 225655_at | ubiquitin like with PHD and ring finger domains 1 |
| LMNB1 | protein coding | -5.82216 | 4.90E-11 | 203276_at | lamin B1 |
| PRSS35 | protein coding | -5.83041 | 9.20E-19 | 235874_at | protease, serine 35 |
| DLGAP5 | protein coding | -5.88112 | 1.11E-11 | 203764_at | DLG associated protein 5 |
| SOX11 | protein coding | -5.91121 | 3.92E-09 | 204914_s_at | SRY-box 11 |
| ANLN | protein coding | -5.97125 | 7.88E-09 | 222608_s_at | anillin actin binding protein |
| HBE1 | protein coding | -6.56733 | 2.24E-19 | 205919_at | hemoglobin subunit epsilon 1 |
| TOP2A | protein coding | -7.14192 | 1.93E-11 | 201291_s_at | topoisomerase (DNA) II alpha |
| HBG1,2 | protein coding | -7.52391 | 1.53E-13 | 204848_x_at | hemoglobin subunit gamma 1,2 |
| DLK1 | protein coding | -7.90473 | 1.91E-10 | 209560_s_at | delta like non-canonical Notch ligand 1 |
| HMGA2 | protein coding | -8.16152 | 1.30E-26 | 208025_s_at | high mobility group AT-hook 2 |

### 1.2. "AT vs. AM"

Table 1.2.A. "AT vs. AM (AT>AM)"

Top 20 protein-coding genes differentially expressed in the adult thyroid (AT) as compared to the adult tissue mix (AM) (comparison 2) and demonstrating a higher expression in the adult thyroid (AT>AM).

| COL_NAME |  | logFC | adj.P.Val | function | gene description |
| --- | --- | --- | --- | --- | --- |
| SLC26A7 | protein coding | 9.631068 | 1.30E-29 | channel | solute carrier family 26 member 7 |
| TG | protein coding | 9.218656 | 5.84E-29 | thyr. horm. | thyroglobulin |
| SLC26A4 | protein coding | 8.473188 | 7.23E-31 | channel | solute carrier family 26 member 4 |
| TSHR | protein coding | 8.462362 | 1.01E-28 | signaling | thyroid stimulating hormone receptor |
| SFTA3 | protein coding | 7.959454 | 1.46E-19 | cytosk/ECM | surfactant associated 3 |
| TPO | protein coding | 7.92671 | 8.98E-28 | thyr. horm | thyroid peroxidase |
| DIO1 | protein coding | 7.55708 | 1.14E-12 | thyr.horm | iodothyronine deiodinase 1 |
| IYD | protein coding | 7.545285 | 2.83E-24 | thyr.horm | iodotyrosine deiodinase |
| FOXE1 | protein coding | 7.372855 | 4.82E-30 | gene exprs. | forkhead box E1 |
| DIO2 | protein coding | 7.272349 | 8.74E-28 | thyr.horm | iodothyronine deiodinase 2 |
| ZBED2 | protein coding | 6.49737 | 8.69E-18 | gene exprs. | zinc finger BED-type containing 2 |
| LIPG | protein coding | 6.371983 | 1.87E-16 | signaling | lipase G, endothelial type |
| NKX2-1 | protein coding | 6.098206 | 3.63E-19 | gene exprs. | NK2 homeobox 1 |
| PKHD1L1 | protein coding | 6.047923 | 1.35E-19 | transport | PKHD1 like 1 |
| MUC15 | protein coding | 5.809554 | 1.11E-19 | cytosk/ECM | mucin 15, cell surface associated |
| TNFRSF11B | protein coding | 5.639884 | 1.27E-21 | signaling | TNF receptor superfamily member 11b |
| KRT7 | protein coding | 5.419558 | 1.42E-10 | cytosk./ECM | keratin 7 |
| LRP2 | protein coding | 5.195709 | 2.06E-15 | transport | LDL receptor related protein 2 |
| CLIC3 | protein coding | 5.18208 | 1.03E-13 | channel (metab) | chloride intracellular channel 3 |
| KLHL14 | protein coding | 5.153441 | 4.92E-14 | transport | kelch like family member 14 |

Table 1.2.B. "AT vs. AM (AT>AM)"

Top 20 protein-coding genes differentially expressed in the adult thyroid (AT) as compared to the adult tissue mix (AM) (comparison 2) and demonstrating a higher expression in the adult tissue mix (AM>AT).

| COL_NAME |  | logFC | adj.P.Val | best probe | gene description |
| --- | --- | --- | --- | --- | --- |
| PLEC | protein coding | -2.96082 | 3.16E-16 | 216971_s_at | plectin |
| TGFB1 | protein coding | -2.96774 | 2.82E-10 | 203085_s_at | transforming growth factor beta 1 |
| LARP1 | protein coding | -2.96947 | 4.97E-19 | 212193_s_at | La ribonucleoprotein domain family member 1 |
| PRKCSH | protein coding | -3.00392 | 3.28E-21 | 200707_at | protein kinase C substrate 80K-H |
| CYB5R3 | protein coding | -3.00709 | 3.39E-17 | 1554574_a_at | cytochrome b5 reductase 3 |
| BANF1 | protein coding | -3.03748 | 5.65E-23 | 210125_s_at | barrier to autointegration factor 1 |
| RASSF3 | protein coding | -3.08918 | 9.86E-09 | 230466_s_at | Ras association domain family member 3 |
| GSTA2 | protein coding | -3.13319 | 0.003971 | 203924_at | glutathione S-transferase alpha 2 |
| OLFM4 | protein coding | -3.1436 | 0.005109 | 212768_s_at | olfactomedin 4 |
| COL6A2 | protein coding | -3.17492 | 4.50E-08 | 209156_s_at | collagen type VI alpha 2 chain |
| HSPD1 | protein coding | -3.22207 | 9.21E-07 | 241716_at | heat shock protein family D (Hsp60) member 1 |
| ST6GALNAC1 | protein coding | -3.24029 | 2.99E-05 | 227725_at | ST6 N-acetylgalactosaminide alpha-2,6-sialyltransferase 1 |
| PLTP | protein coding | -3.27957 | 4.73E-14 | 202075_s_at | phospholipid transfer protein |
| TOX3 | protein coding | -3.45961 | 1.09E-05 | 216623_x_at | TOX high mobility group box family member 3 |
| EPHX1 | protein coding | -3.50893 | 2.48E-13 | 202017_at | epoxide hydrolase 1 |
| PKM | protein coding | -3.5713 | 2.36E-16 | 201251_at | pyruvate kinase, muscle |
| CFL1 | protein coding | -3.71048 | 5.24E-19 | 1555730_a_at | cofilin 1 |
| SCD | protein coding | -3.83517 | 1.24E-08 | 200832_s_at | stearoyl-CoA desaturase |
| AZGP1 | protein coding | -3.84381 | 0.000604 | 209309_at | alpha-2-glycoprotein 1, zinc-binding |
| TRAPPC1 | protein coding | -3.9 | 4.74E-26 | 225294_s_at | trafficking protein particle complex 1 |

### 1.3. "AM vs. EM"

Table 1.3.A. "AM vs. EM (AM>EM)"

Top 20 protein-coding genes differentially expressed in the adult tissue mix (AM) as compared to the embryonic tissue mix (EM) (comparison 3) and demonstrating a higher expression in the adult tissue mix (AM>AT).

| COL_NAME |  | logFC | adj.P.Val | best probe |  |
| --- | --- | --- | --- | --- | --- |
| IGHG3,G1,G2,M,V4-31 | IG_C_gene, IG_V_gene | 6.253945 | 3.20E-07 | 211430_s_at | #N/A |
| IGHA1,2 | IG_C_gene | 6.098275 | 3.43E-06 | 217022_s_at | #N/A |
| HLA-DPA1 | protein coding | 5.987627 | 2.19E-13 | 211990_at | major histocompatibility complex, class II, DP alpha 1 |
| IFI27 | protein coding | 5.56371 | 4.43E-17 | 202411_at | interferon alpha inducible protein 27 |
| HLA-DRA | protein coding | 5.497661 | 2.11E-12 | 208894_at | major histocompatibility complex, class II, DR alpha |
| IGLC1,J3 | IG_C_gene, IG_J_gene | 5.407932 | 7.49E-06 | 214677_x_at | #N/A |
| HLA-DRB3 | protein coding | 5.401997 | 4.53E-13 | 215193_x_at | major histocompatibility complex, class II, DR beta 3 |
| HLA-DRB1,4,5,DQB1 | protein coding | 5.132803 | 2.03E-13 | 209312_x_at | major histocompatibility complex, class II, DR beta 1,4,5,DQ beta 2 |
| HLA-DQA1,2 | protein coding | 5.051029 | 8.13E-09 | 212671_s_at | major histocompatibility complex, class II, DQ alpha 1,2 |
| JCHAIN | protein coding | 4.975144 | 6.42E-05 | 212592_at | joining chain of multimeric IgA and IgM |
| ADH1B | protein coding | 4.890433 | 3.56E-06 | 209613_s_at | alcohol dehydrogenase 1B (class I), beta polypeptide |
| IGKC | IG_C_gene | 4.74637 | 1.66E-06 | 224795_x_at | 0 |
| HLA-DMA | protein coding | 4.72932 | 2.05E-11 | 217478_s_at | major histocompatibility complex, class II, DM alpha |
| TRIM22 | protein coding | 4.447107 | 3.25E-13 | 213293_s_at | tripartite motif containing 22 |
| PSMB9 | protein coding | 4.331682 | 2.01E-10 | 204279_at | proteasome subunit beta 9 |
| HLA-B | protein coding | 4.331322 | 6.95E-15 | 209140_x_at | major histocompatibility complex, class I, B |
| PDK4 | protein coding | 4.239651 | 5.56E-10 | 225207_at | pyruvate dehydrogenase kinase 4 |
| IGLV1-44 | IG_V_gene | 4.182397 | 3.91E-05 | 215379_x_at | 0 |
| ADIRF | protein coding | 4.163214 | 2.94E-10 | 203571_s_at | adipogenesis regulatory factor |
| PIGR | protein coding | 4.13606 | 0.003605 | 226147_s_at | polymeric immunoglobulin receptor |

Table 1.3.B. "AM vs. EM (EM>AM)"

Top 20 protein-coding genes differentially expressed in the adult tissue mix (AM) as compared to the embryonic tissue mix (EM) (comparison 3) and demonstrating a higher expression in the embryonic tissue mix (EM>AM).

| COL_NAME |  | logFC | adj.P.Val | best probe |  |
| --- | --- | --- | --- | --- | --- |
| STMN2 | protein coding | -5.57504 | 4.83E-07 | 203000_at | stathmin 2 |
| MYH3 | protein coding | -5.57717 | 1.04E-10 | 205940_at | myosin heavy chain 3 |
| TMSB15A,B | protein coding | -5.85625 | 2.88E-11 | 205347_s_at | thymosin beta 15a, 15B |
| POSTN | protein coding | -5.96238 | 9.49E-12 | 1555778_a_at | periostin |
| ZIC1 | protein coding | -5.97253 | 2.04E-08 | 206373_at | Zic family member 1 |
| APOB | protein coding | -6.16233 | 3.05E-08 | 205108_s_at | apolipoprotein B |
| DLK1 | protein coding | -6.34142 | 2.76E-10 | 209560_s_at | delta like non-canonical Notch ligand 1 |
| COL11A1 | protein coding | -6.36135 | 1.38E-19 | 204320_at | collagen type XI alpha 1 chain |
| FBN2 | protein coding | -6.43852 | 1.59E-15 | 203184_at | fibrillin 2 |
| HBG1,2 | protein coding | -6.91117 | 4.89E-15 | 204848_x_at | hemoglobin subunit gamma 1, 2 |
| COL2A1 | protein coding | -7.07999 | 1.25E-18 | 217404_s_at | collagen type II alpha 1 chain |
| EPYC | protein coding | -7.1039 | 2.22E-18 | 206439_at | epiphycan |
| DCX | protein coding | -7.25707 | 1.59E-12 | 204851_s_at | doublecortin |
| HMGA2 | protein coding | -7.53404 | 1.97E-28 | 208025_s_at | high mobility group AT-hook 2 |
| SOX11 | protein coding | -7.56468 | 1.03E-14 | 204914_s_at | SRY-box 11 |
| APOA2 | protein coding | -7.84903 | 6.53E-13 | 219465_at | apolipoprotein A2 |
| AHSG | protein coding | -8.0258 | 3.57E-13 | 210929_s_at | alpha 2-HS glycoprotein |
| HBZ | protein coding | -8.31825 | 1.51E-20 | 206647_at | hemoglobin subunit zeta |
| HBE1 | protein coding | -8.37737 | 1.34E-26 | 205919_at | hemoglobin subunit epsilon 1 |
| AFP | protein coding | -8.60913 | 2.15E-26 | 204694_at | alpha fetoprotein |

### 1.4. "ET vs. EM"

Table 1.4.A. "ET vs. EM (ET>EM)"

Top 20 protein-coding genes differentially expressed in the embryonic thyroid (ET) as compared to the embryonic tissue mix (EM) (comparison 4) and demonstrating a higher expression in the embryonic thyroid (ET>EM).

| COL_NAME |  | logFC | adj.P.Val | function | gene description |
| --- | --- | --- | --- | --- | --- |
| SLC26A7 | protein coding | 8.272029 | 1.85E-22 | channel | solute carrier family 26 member 7 |
| SFTA3 | protein coding | 7.878599 | 2.13E-15 | cytosk/ECM | surfactant associated 3 |
| DIO2 | protein coding | 7.592935 | 1.02E-23 | thyr.horm | iodothyronine deiodinase 2 |
| FOXE1 | protein coding | 7.322695 | 3.21E-25 | gene exprs. | forkhead box E1 |
| TSHR | protein coding | 7.10212 | 3.23E-21 | signaling | thyroid stimulating hormone receptor |
| TG | protein coding | 6.484453 | 1.09E-18 | thyr.horm | thyroglobulin |
| IYD | protein coding | 5.788052 | 8.47E-16 | thyr.horm | iodotyrosine deiodinase |
| SLC26A4 | protein coding | 5.707313 | 4.49E-20 | channel | solute carrier family 26 member 4 |
| PTH | protein coding | 5.402622 | 0.000819 | signaling | parathyroid hormone |
| AQP4 | protein coding | 5.315306 | 0.000541 | channel | aquaporin 4 |
| LIPG | protein coding | 5.258327 | 2.48E-10 | signaling | lipase G, endothelial type |
| ENPP5 | protein coding | 5.061652 | 5.73E-08 | signaling | ectonucleotide pyrophosphatase/phosphodiesterase 5 (putative) |
| LY75 | protein coding | 5.012143 | 2.86E-07 | transport | lymphocyte antigen 75 |
| EPCAM | protein coding | 4.986879 | 4.49E-05 | cytosk/ECM | epithelial cell adhesion molecule |
| PPP1R14C | protein coding | 4.968916 | 2.66E-08 | signaling | protein phosphatase 1 regulatory inhibitor subunit 14C |
| ZBED2 | protein coding | 4.818957 | 4.21E-10 | gene exprs. | zinc finger BED-type containing 2 |
| DUOX2 | protein coding | 4.815586 | 2.79E-07 | thyr.horm | dual oxidase 2 |
| ATP8B1 | protein coding | 4.719274 | 1.34E-05 | transport | ATPase phospholipid transporting 8B1 |
| KCNAB1 | protein coding | 4.693542 | 6.83E-14 | channel | potassium voltage-gated channel subfamily A member regulatory beta subunit 1 |
| RBM47 | protein coding | 4.687711 | 3.86E-06 | ene exprs. | RNA binding motif protein 47 |

Table 1.4.B. "ET vs. EM (EM>ET)"

Top 20 protein-coding genes differentially expressed in the embryonic thyroid (ET) as compared to the embryonic tissue mix (EM) (comparison 4) and demonstrating a higher expression in the embryonic tissue mix (EM>ET).

| COL_NAME |  | logFC | adj.P.Val | best probe | gene description |
| --- | --- | --- | --- | --- | --- |
| FABP7 | protein coding | -5.18397 | 1.35E-05 | 205029_s_at | fatty acid binding protein 7 |
| CXCL14 | protein coding | -5.25799 | 2.86E-07 | 222484_s_at | C-X-C motif chemokine ligand 14 |
| POU3F3 | protein coding | -5.39757 | 1.51E-05 | 228780_at | POU class 3 homeobox 3 |
| AMBP | protein coding | -5.46722 | 4.35E-06 | 205477_s_at | alpha-1-microglobulin/bikunin precursor |
| EPYC | protein coding | -5.62903 | 3.77E-12 | 206439_at | epiphycan |
| NHLH2 | protein coding | -5.68506 | 8.66E-14 | 215228_at | nescient helix-loop-helix 2 |
| ITIH2 | protein coding | -5.73345 | 3.75E-07 | 204987_at | inter-alpha-trypsin inhibitor heavy chain 2 |
| HOXC6 | protein coding | -5.73884 | 1.57E-07 | 206858_s_at | homeobox C6 |
| TF | protein coding | -5.7839 | 1.74E-05 | 214063_s_at | transferrin |
| FGG | protein coding | -5.78808 | 2.34E-05 | 219612_s_at | fibrinogen gamma chain |
| APOH | protein coding | -5.96962 | 2.18E-06 | 205216_s_at | apolipoprotein H |
| ALB | protein coding | -6.11627 | 2.39E-08 | 214842_s_at | albumin |
| FGB | protein coding | -6.38446 | 1.11E-07 | 204988_at | fibrinogen beta chain |
| ZIC2 | protein coding | -6.62083 | 5.15E-09 | 223642_at | Zic family member 2 |
| ZIC1 | protein coding | -6.9245 | 1.04E-07 | 206373_at | Zic family member 1 |
| APOB | protein coding | -7.01039 | 2.26E-07 | 205108_s_at | apolipoprotein B |
| SERPINA1 | protein coding | -8.22444 | 1.69E-08 | 202833_s_at | serpin family A member 1 |
| AHSG | protein coding | -8.38632 | 4.89E-11 | 210929_s_at | alpha 2-HS glycoprotein |
| AFP | protein coding | -8.40866 | 1.85E-22 | 204694_at | alpha fetoprotein |
| APOA2 | protein coding | -8.65092 | 2.00E-11 | 219465_at | apolipoprotein A2 |
