# Supplementary material for: Transcriptomic Signature of Human Embryonic Thyroid Reveals Transition From Differentiation to Functional Maturation
Source: Front Cell Dev Biol. 2021 Jun 11;9:669354. doi: 10.3389/fcell.2021.669354 (PMC8270686; doi:10.3389/fcell.2021.669354)
Supplement: Supplementary file 7 [file Table_6.docx]

**Supplementary table 6**:

# Top 20 adult and embryonic thyroid-specific genes

Contents

[Supplementary file DOCX-2. Top 20 adult and embryonic thyroid-specific genes 1](#_Toc62800621)

[File description 1](#_Toc62800622)

[Group 1. Top 20 genes co-regulated in the adult and embryonic thyroid. 2](#_Toc62800623)

[Group 2. Genes specific to adult thyroid 3](#_Toc62800624)

[Group 3. Genes specific to embryonic thyroid 4](#_Toc62800625)

## File description

The file contains lists of 20 genes and non-annotated probes with the strongest fold change co-regulated in the adult and embryonic thyroid (group 1) or specifically up- or downregulated in adult or embryonic thyroid compared to corresponding tissue mix (groups 2 and 3 respectively) .

These lists originate from the **Intersection 1** (comparison of gene expression in adult and embryonic thyroid, for details see **Supplementary file 2. Supplementary results and discussion.docx**)

A separate top 20 list are provided for each of the following types of genes 1.prot coding, 2.snoRNA, 3.miRNA, 4.pseudogene, 5.#NA (non-classified loci), 6.ncRNAeach.

## Group 1. Top 20 genes co-regulated in the adult and embryonic thyroid.

| intersection 1 | | | | | | |
| --- | --- | --- | --- | --- | --- | --- |
| comparison 2 (AT vs. AM) | | |  | comparison 4 (ET vs. EM) | | |
| Adult thyroid (AT)  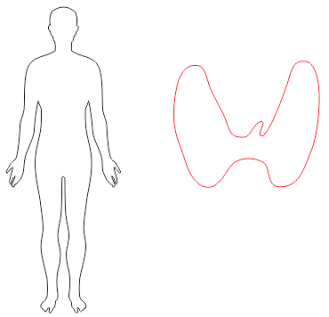 | > | Adult tissue mix (AM)  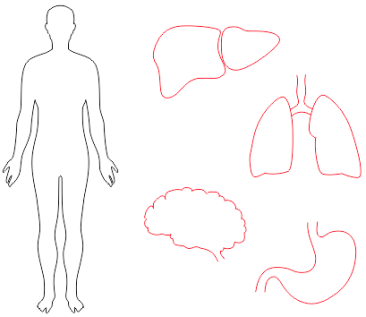 | AND | Embryonic thyroid (ET)  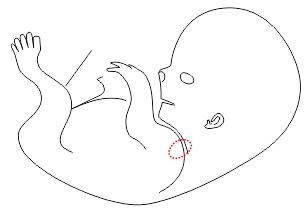 | > | Embryonic tissue mix (EM)  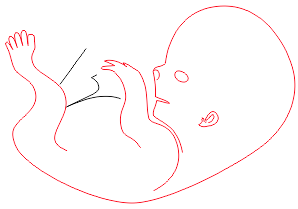 |
|  | | | | | | |
| Adult thyroid (AT)  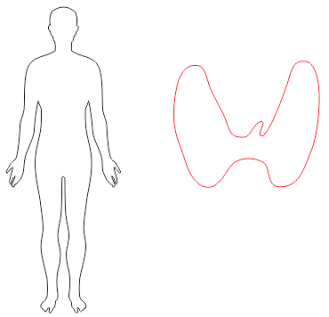 | < | Adult tissue mix (AM)  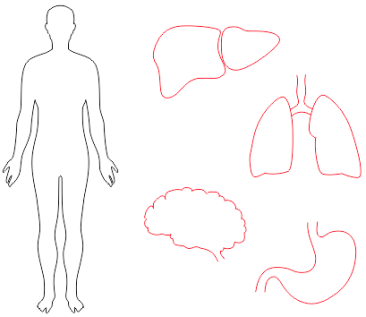 | AND | Embryonic thyroid (ET)  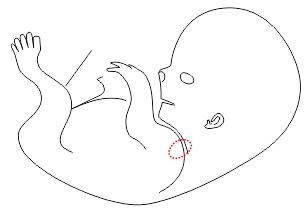 | < | Embryonic tissue mix (EM)  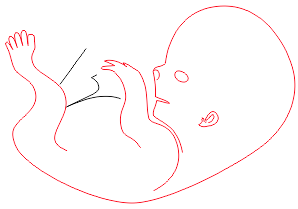 |
| Group 1 contains top 20 genes differentially expressed and upregulated (adj.p-value < 0.05; log2FC > 1) or downregulated (adj.p-value < 0.05; log2FC < -1) in both adult thyroid (AT) and embryonic thyroid (ET) compared to adult tissue mix (AM) and embryonic tissue mix (EM) respectively. | | | | | | |

Table 1.1.A protein-coding, co-upregulated in the adult thyroid and embryonic thyroid as compared to adult and embryonic tissue mixes

|  | ### | ATAM | ATAM | ETEM | ETEM |  | ### |
| --- | --- | --- | --- | --- | --- | --- | --- |
|  | gene type | logFC | adj.P.Val | logFC | adj.P.Val | function | gene description |
| AQP4 | protein coding | 3.298846 | 0.004244 | 5.315306 | 0.000541 | channel | aquaporin 4 |
| C16orf89 | protein coding | 4.595242 | 3.28E-15 | 4.231758 | 1.22E-10 |  | chromosome 16 open reading frame 89 |
| CLIC3 | protein coding | 5.18208 | 1.03E-13 | 3.818752 | 3.13E-07 | channel (metabolism?) | chloride intracellular channel 3 |
| DIO2 | protein coding | 7.272349 | 8.74E-28 | 7.592935 | 1.02E-23 | thyr.horm | iodothyronine deiodinase 2 |
| DUOX2 | protein coding | 4.501804 | 2.66E-09 | 4.815586 | 2.79E-07 | thyr.horm | dual oxidase 2 |
| EPCAM | protein coding | 4.096718 | 1.30E-05 | 4.986879 | 4.49E-05 | cytosk/ECM | epithelial cell adhesion molecule |
| FOXE1 | protein coding | 7.372855 | 4.82E-30 | 7.322695 | 3.21E-25 | gene exprs. | forkhead box E1 |
| IYD | protein coding | 7.545285 | 2.83E-24 | 5.788052 | 8.47E-16 | thyr.horm | iodotyrosine deiodinase |
| KLHL14 | protein coding | 5.153441 | 4.92E-14 | 4.172048 | 2.46E-08 |  | kelch like family member 14 |
| LIPG | protein coding | 6.371983 | 1.87E-16 | 5.258327 | 2.48E-10 | signaling | lipase G, endothelial type |
| MUC15 | protein coding | 5.809554 | 1.11E-19 | 2.863763 | 3.14E-07 | cytosk/ECM | mucin 15, cell surface associated |
| NKX2-1 | protein coding | 6.098206 | 3.63E-19 | 2.577706 | 4.59E-14 | gene exprs. | NK2 homeobox 1 |
| PPP1R14C | protein coding | 3.819874 | 2.07E-08 | 4.968916 | 2.66E-08 | signaling | protein phosphatase 1 regulatory inhibitor subunit 14C |
| SFTA3 | protein coding | 7.959454 | 1.46E-19 | 7.878599 | 2.13E-15 | cytosk/ECM | surfactant associated 3 |
| SLC26A4 | protein coding | 8.473188 | 7.23E-31 | 5.707313 | 4.49E-20 | channel | solute carrier family 26 member 4 |
| SLC26A7 | protein coding | 9.631068 | 1.30E-29 | 8.272029 | 1.85E-22 | channel | solute carrier family 26 member 7 |
| TG | protein coding | 9.218656 | 5.84E-29 | 6.484453 | 1.09E-18 | thyr.horm | thyroglobulin |
| TPO | protein coding | 7.92671 | 8.98E-28 | 2.845269 | 9.47E-09 | thyr.horm | thyroid peroxidase |
| TSHR | protein coding | 8.462362 | 1.01E-28 | 7.10212 | 3.23E-21 | signaling | thyroid stimulating hormone receptor |
| ZBED2 | protein coding | 6.49737 | 8.69E-18 | 4.818957 | 4.21E-10 | gene exprs. | zinc finger BED-type containing 2 |

Table 1.1.A protein-coding genes co-downregulated in the adult thyroid and embryonic thyroid

|  | ### | ATAM | ATAM | ETEM | ETEM | ### |
| --- | --- | --- | --- | --- | --- | --- |
|  | gene type | logFC | adj.P.Val | logFC | adj.P.Val | gene description |
| SPTAN1 | protein coding | -2.87824 | 6.75E-12 | -3.12635 | 1.05E-09 | spectrin alpha, non-erythrocytic 1 |
| EPHX1 | protein coding | -3.50893 | 2.48E-13 | -2.55685 | 6.85E-07 | epoxide hydrolase 1 |
| NEFL | protein coding | -2.55306 | 0.014417 | -3.51791 | 0.003071 | neurofilament light |
| HMGCS2 | protein coding | -2.75127 | 0.004226 | -3.33518 | 0.008165 | 3-hydroxy-3-methylglutaryl-CoA synthase 2 |
| RBM6 | protein coding | -2.54675 | 2.14E-10 | -3.65356 | 1.82E-11 | RNA binding motif protein 6 |
| CFL1 | protein coding | -3.71048 | 5.24E-19 | -2.51775 | 5.11E-10 | cofilin 1 |
| PKM | protein coding | -3.5713 | 2.36E-16 | -2.72238 | 2.24E-09 | pyruvate kinase, muscle |
| TOX3 | protein coding | -3.45961 | 1.09E-05 | -2.84135 | 0.003874 | TOX high mobility group box family member 3 |
| HOXA9, 10 | protein coding | -2.20031 | 0.000161 | -4.34232 | 1.6E-07 | homeobox A9 |
| ARHGEF7 | protein coding | -2.49268 | 7.38E-08 | -4.08883 | 3.74E-10 | Rho guanine nucleotide exchange factor 7 |
| TRAPPC1 | protein coding | -3.9 | 4.74E-26 | -2.71596 | 7.48E-16 | trafficking protein particle complex 1 |
| SPINK1 | protein coding | -2.87516 | 0.019766 | -3.85083 | 0.017793 | serine peptidase inhibitor, Kazal type 1 |
| GSTA2 | protein coding | -3.13319 | 0.003971 | -3.89488 | 0.006307 | glutathione S-transferase alpha 2 |
| SCD | protein coding | -3.83517 | 1.24E-08 | -3.2157 | 1.25E-07 | stearoyl-CoA desaturase |
| STMN2 | protein coding | -2.7293 | 0.006931 | -4.42039 | 0.000571 | stathmin 2 |
| ALDOB | protein coding | -2.36553 | 0.036029 | -4.84073 | 0.001228 | aldolase, fructose-bisphosphate B |
| RBP4 | protein coding | -2.3143 | 0.006662 | -4.9897 | 2.13E-05 | retinol binding protein 4 |
| TF | protein coding | -2.74716 | 0.004955 | -5.7839 | 1.74E-05 | transferrin |
| ZIC1 | protein coding | -1.78023 | 0.045042 | -6.9245 | 1.04E-07 | Zic family member 1 |
| SERPINA1 | protein coding | -2.28816 | 0.018538 | -8.22444 | 1.69E-08 | serpin family A member 1 |

## Group 2. Genes specific to adult thyroid

| intersection 1 | | | | | | |
| --- | --- | --- | --- | --- | --- | --- |
| comparison 2 (AT vs. AM) | | |  | comparison 4 (ET vs. EM) | | |
| Adult thyroid (AT)  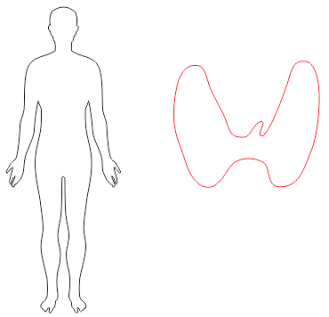 | > | Adult tissue mix (AM)  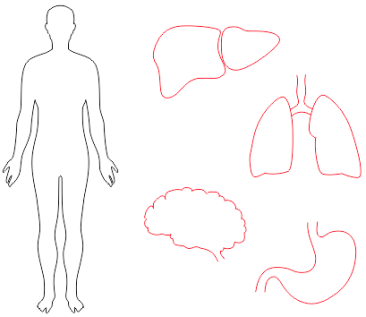 | AND | Embryonic thyroid (ET)  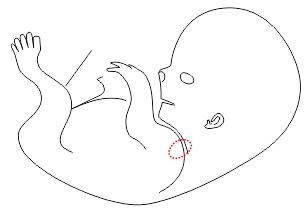 | =  OR  < | Embryonic tissue mix (EM)  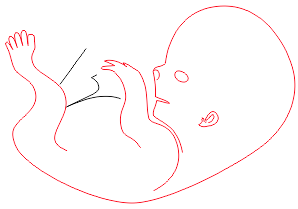 |
|  | | | | | | |
| Adult thyroid (AT)  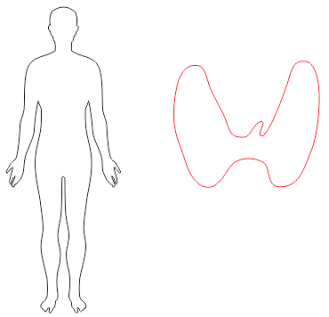 | < | Adult tissue mix (AM)  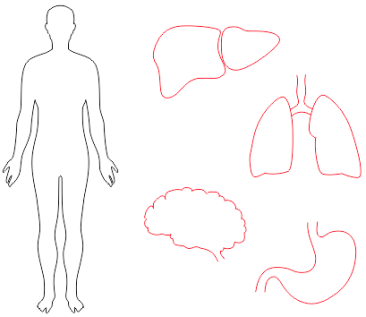 | AND | Embryonic thyroid (ET)  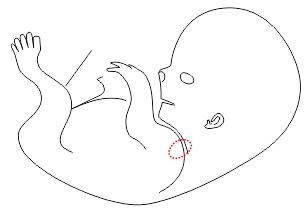 | =  OR  > | Embryonic tissue mix (EM)  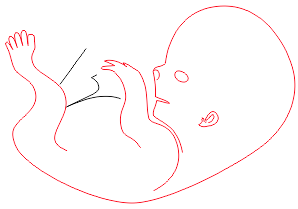 |
| Group 2 contains top 20 genes differentially expressed and upregulated (adj.p-value < 0.05; log2FC > 1) or downregulated (adj.p-value < 0.05; log2FC < -1) in adult thyroid (AT) but not embryonic thyroid (ET) compared to adult tissue mix (AM) and embryonic tissue mix (EM) respectively. | | | | | | |

**Table 2.1.A** Top 20 protein-coding genes upregulated in the adult thyroid but not embryonic thyroid as compared to adult and embryonic tissue mixes

|  | ### | ATAM | ATAM | ETEM | ETEM |  | ### |
| --- | --- | --- | --- | --- | --- | --- | --- |
|  | gene type | logFC | adj.P.Val | logFC | adj.P.Val | function | gene description |
| ART4 | protein coding | 3.033284 | 5.40E-08 | -1.36524 | 0.018306 | transport | ADP-ribosyltransferase 4 (Dombrock blood group) |
| BHLHE41 | protein coding | 3.559718 | 4.36E-13 | -0.8529 | 0.001363 | gene exprs. | basic helix-loop-helix family member e41 |
| CLIC2 | protein coding | 3.331843 | 5.91E-09 | 0.001232 | 0.998797 | channel (metabolism? | chloride intracellular channel 2 |
| CRABP1 | protein coding | 3.168692 | 4.50E-10 | -2.8007 | 2.90E-06 | signaling | cellular retinoic acid binding protein 1 |
| CRYBG3 | protein coding | 3.082925 | 1.12E-10 | 0.416732 | 0.465342 | cytosk/ECM | crystallin beta-gamma domain containing 3 |
| DGKI | protein coding | 5.060806 | 1.27E-17 | 0.301948 | 0.607274 | signaling | diacylglycerol kinase iota |
| DMRT3 | protein coding | 3.408965 | 1.74E-10 | -0.3192 | 0.04467 | gene exprs. | doublesex and mab-3 related transcription factor 3 |
| EPHA3 | protein coding | 3.949323 | 7.00E-12 | -0.08579 | 0.409434 | signaling | EPH receptor A3 |
| IGFBPL1 | protein coding | 3.24493 | 1.59E-11 | -2.26046 | 1.98E-05 | signaling | insulin like growth factor binding protein like 1 |
| LRP8 | protein coding | 3.734844 | 2.94E-08 | -0.64155 | 0.404902 | transport | LDL receptor related protein 8 |
| MPPED2 | protein coding | 3.592686 | 2.03E-09 | -1.55516 | 0.003349 | signaling | metallophosphoesterase domain containing 2 |
| MT1F | protein coding | 3.455279 | 2.59E-08 | -1.39627 | 0.035196 | transport (thyr horm?) | metallothionein 1F |
| NEBL | protein coding | 3.890097 | 3.53E-06 | -0.94516 | 0.010725 | cytosk/ECM | nebulette |
| OMD | protein coding | 3.537622 | 2.14E-11 | -0.44249 | 0.493031 | cytosk/ECM | osteomodulin |
| PDE10A | protein coding | 3.536237 | 7.02E-11 | -0.43908 | 0.048794 | signaling | phosphodiesterase 10A |
| RBMS3 | protein coding | 3.094621 | 2.96E-14 | -1.66336 | 4.58E-05 | gene exprs. | RNA binding motif single stranded interacting protein 3 |
| RNF128 | protein coding | 3.496623 | 8.64E-05 | 0.649554 | 0.621389 | transport | ring finger protein 128, E3 ubiquitin protein ligase |
| SCUBE3 | protein coding | 4.619186 | 1.35E-19 | -1.00745 | 0.00012 |  | signal peptide, CUB domain and EGF like domain containing 3 |
| SERTM1 | protein coding | 3.070198 | 5.12E-07 | 0.281074 | 0.752858 |  | serine rich and transmembrane domain containing 1 |
| SUCNR1 | protein coding | 3.684156 | 6.38E-10 | -0.42434 | 0.572185 |  | succinate receptor 1 |

**Table 2.1.B** Top 20 protein-coding, genes downregulated in the adult thyroid but not embryonic thyroid as compared to adult and embryonic tissue mixes

|  | ### | ATAM | ATAM | ETEM | ETEM | ### |
| --- | --- | --- | --- | --- | --- | --- |
|  | gene type | logFC | adj.P.Val | logFC | adj.P.Val | gene description |
| MBP | protein coding | -2.44112 | 0.010633 | 0.980128 | 0.005109 | myelin basic protein |
| GDA | protein coding | -2.49827 | 0.000882 | 0.206054 | 0.869497 | guanine deaminase |
| DEFA1, 1B, 3 | protein coding | -2.50633 | 0.011564 | 0.702343 | 0.645399 | defensin alpha 1, 1B, 3 |
| IDH2 | protein coding | -2.54556 | 3.2E-08 | 1.047184 | 0.023111 | isocitrate dehydrogenase (NADP(+)) 2, mitochondrial |
| UHRF1 | protein coding | -2.54709 | 1.7E-05 | 0.236781 | 0.796263 | ubiquitin like with PHD and ring finger domains 1 |
| G0S2 | protein coding | -2.56404 | 1.09E-05 | -0.16555 | 0.858669 | G0/G1 switch 2 |
| DNAJC12 | protein coding | -2.56462 | 3.79E-06 | -0.30644 | 0.404412 | DnaJ heat shock protein family (Hsp40) member C12 |
| FAM83D | protein coding | -2.58112 | 0.000121 | 0.330012 | 0.750538 | family with sequence similarity 83 member D |
| PPP1R14A | protein coding | -2.58135 | 1.71E-06 | 0.348996 | 0.65155 | protein phosphatase 1 regulatory inhibitor subunit 14A |
| PIGR | protein coding | -2.61795 | 0.002994 | -0.60076 | 0.657138 | polymeric immunoglobulin receptor |
| GPX2 | protein coding | -2.63236 | 0.001417 | -0.22404 | 0.630709 | glutathione peroxidase 2 |
| LCN2 | protein coding | -2.65764 | 0.001434 | -0.50886 | 0.693342 | lipocalin 2 |
| IL32 | protein coding | -2.67414 | 3.52E-05 | -0.75446 | 0.396113 | interleukin 32 |
| PLA2G2A | protein coding | -2.69981 | 0.001924 | -0.20568 | 0.889309 | phospholipase A2 group IIA |
| PPP1R1B | protein coding | -2.84248 | 6.14E-06 | -0.50028 | 0.574068 | protein phosphatase 1 regulatory inhibitor subunit 1B |
| TPSB2 | protein coding | -2.86549 | 1.29E-05 | -0.57625 | 0.38787 | tryptase beta 2 (gene/pseudogene) |
| RASSF3 | protein coding | -3.08918 | 9.86E-09 | 0.822633 | 0.002551 | Ras association domain family member 3 |
| OLFM4 | protein coding | -3.1436 | 0.005109 | -0.72153 | 0.67867 | olfactomedin 4 |
| ST6GALNAC1 | protein coding | -3.24029 | 2.99E-05 | -0.79651 | 0.462066 | ST6 N-acetylgalactosaminide alpha-2,6-sialyltransferase 1 |
| AZGP1 | protein coding | -3.84381 | 0.000604 | -0.43424 | 0.809724 | alpha-2-glycoprotein 1, zinc-binding |

## Group 3. Genes specific to embryonic thyroid

| intersection 1 | | | | | | |
| --- | --- | --- | --- | --- | --- | --- |
| comparison 2 (AT vs. AM) | | |  | comparison 4 (ET vs. EM) | | |
| Adult thyroid (AT)  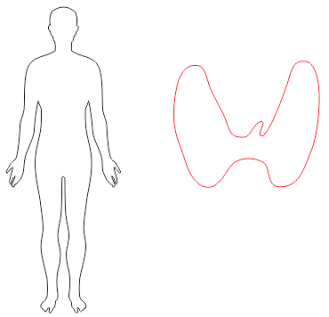 | =  OR  < | Adult tissue mix (AM)  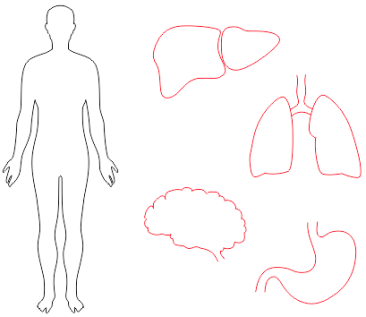 | AND | Embryonic thyroid (ET)  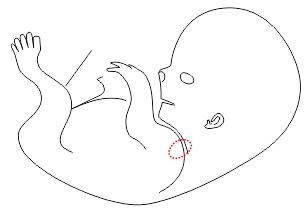 | > | Embryonic tissue mix (EM)  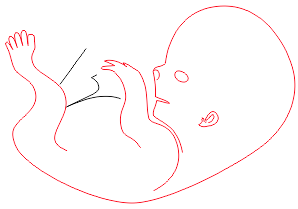 |
|  | | | | | | |
| Adult thyroid (AT)  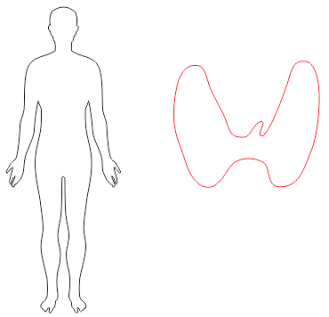 | =  OR  < | Adult tissue mix (AM)  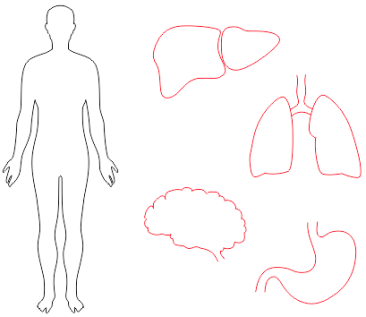 | AND | Embryonic thyroid (ET)  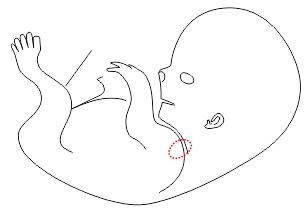 | < | Embryonic tissue mix (EM)  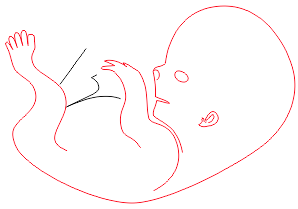 |
| Group 3 contains top 20 genes differentially expressed and upregulated (adj.p-value < 0.05; log2FC > 1) or downregulated (adj.p-value < 0.05; log2FC < -1) in embryonic thyroid (ET) but not adult thyroid (AT) compared to embryonic tissue mix (EM) and adult tissue mix (AM) respectively. | | | | | | |

**Table 3.1.A** Top 20 protein-coding genes upregulated in the embryonic thyroid but not adult thyroid as compared to embryonic and adult tissue mixes respectively.

|  | ### | ATAM | ATAM | ETEM | ETEM |  | ### |
| --- | --- | --- | --- | --- | --- | --- | --- |
|  | gene type | logFC | adj.P.Val | logFC | adj.P.Val | function | gene description |
| ATP11A | protein coding | -0.52964 | 0.002928 | 3.215099 | 1.10E-11 | transport | ATPase phospholipid transporting 11A |
| BLNK | protein coding | 0.531528 | 0.388644 | 3.113306 | 0.000107 | signaling | B-cell linker |
| CEACAM6 | protein coding | -2.37722 | 0.007404 | 2.979284 | 0.01708 |  | carcinoembryonic antigen related cell adhesion molecule 6 |
| CHRNA9 | protein coding | 0.029191 | 0.874475 | 3.424887 | 4.82E-21 | signaling | cholinergic receptor nicotinic alpha 9 subunit |
| CYP4X1 | protein coding | 0.236778 | 0.679254 | 2.776293 | 9.79E-05 | thyr.horm | cytochrome P450 family 4 subfamily X member 1 |
| FREM3 | protein coding | -0.02585 | 0.936978 | 3.670677 | 7.99E-14 | cytosk/ECM | FRAS1 related extracellular matrix 3 |
| GCM2 | protein coding | 0.439484 | 0.365995 | 2.594654 | 5.07E-05 | gene exprs. | glial cells missing homolog 2 |
| GPR160 | protein coding | 0.184974 | 0.80477 | 2.844858 | 0.00131 | signaling | G protein-coupled receptor 160 |
| KCNK5 | protein coding | 0.325788 | 0.487917 | 2.586907 | 2.41E-05 | channel | potassium two pore domain channel subfamily K member 5 |
| LUZP1 | protein coding | -0.65191 | 3.75E-05 | 3.585298 | 2.47E-12 |  | leucine zipper protein 1 |
| MAP4 | protein coding | -1.04965 | 1.63E-05 | 3.185623 | 1.51E-10 | cytosk/ECM | microtubule associated protein 4 |
| NPFFR2 | protein coding | 0.160329 | 0.488923 | 2.955492 | 4.43E-14 | signaling | neuropeptide FF receptor 2 |
| OLFM3 | protein coding | -0.22152 | 0.545568 | 3.716482 | 3.07E-11 | cytosk/ECM | olfactomedin 3 |
| PCDH20 | protein coding | -1.04898 | 0.013744 | 3.603053 | 1.69E-08 | cytosk/ECM | protocadherin 20 |
| PERP | protein coding | -0.59645 | 9.11E-05 | 2.795434 | 0.002299 |  | PERP, TP53 apoptosis effector |
| PSMB8 | protein coding | 0.026024 | 0.964025 | 2.740394 | 4.45E-05 | transport | proteasome subunit beta 8 |
| SAMHD1 | protein coding | -0.74492 | 0.034238 | 3.246975 | 2.22E-07 |  | SAM and HD domain containing deoxynucleoside triphosphate triphosphohydrolase 1 |
| SLC15A2 | protein coding | -1.31991 | 0.000876 | 3.787592 | 9.28E-07 | channel | solute carrier family 15 member 2 |
| SLC22A3 | protein coding | -1.34521 | 0.031774 | 3.64487 | 2.40E-05 | channel | solute carrier family 22 member 3 |
| TNFRSF11A | protein coding | 0.489417 | 0.469523 | 2.92787 | 0.000637 | signaling | TNF receptor superfamily member 11a |

**Table 3.1.B** Top 20 protein-coding, genes downregulated in the embryonic thyroid but not adult thyroid as compared to embryonic and adult tissue mixes respectively.

|  | ### | ATAM | ATAM | ETEM | ETEM | ### |
| --- | --- | --- | --- | --- | --- | --- |
|  | gene type | logFC | adj.P.Val | logFC | adj.P.Val | gene description |
| ADH1A | protein coding | -0.42522 | 0.557621 | -3.74373 | 5.36E-05 | alcohol dehydrogenase 1A (class I), alpha polypeptide |
| CPS1 | protein coding | -0.19966 | 0.832842 | -3.797 | 0.000249 | carbamoyl-phosphate synthase 1 |
| SHOX2 | protein coding | -0.21135 | 0.407053 | -3.84073 | 2.34E-16 | short stature homeobox 2 |
| GSTA1 | protein coding | 0.517478 | 0.000419 | -3.89488 | 0.006307 | glutathione S-transferase alpha 1 |
| CYP3A7 | protein coding | -0.07285 | 0.431591 | -3.91141 | 3.57E-06 | cytochrome P450 family 3 subfamily A member 7 |
| CRYBA1 | protein coding | -0.148 | 0.656626 | -3.9815 | 1.25E-13 | crystallin beta A1 |
| RPL27A | protein coding | 0.364588 | 0.000841 | -4.08601 | 4.1E-18 | ribosomal protein L27a |
| H3F3A | protein coding | 0.502249 | 0.001378 | -4.14647 | 1.67E-17 | H3 histone family member 3A |
| SERPIND1 | protein coding | -0.72822 | 0.356933 | -4.25475 | 4.54E-05 | serpin family D member 1 |
| LHX2 | protein coding | -0.38343 | 0.486962 | -4.40255 | 7.45E-05 | LIM homeobox 2 |
| KNG1 | protein coding | -0.8266 | 0.411722 | -4.42578 | 0.000593 | kininogen 1 |
| CPB2 | protein coding | -0.50705 | 0.564256 | -4.44317 | 7.25E-05 | carboxypeptidase B2 |
| RPS11 | protein coding | 0.842525 | 0.00011 | -5.01902 | 9.04E-23 | ribosomal protein S11 |
| AMBP | protein coding | -0.69076 | 0.436701 | -5.46722 | 4.35E-06 | alpha-1-microglobulin/bikunin precursor |
| EPYC | protein coding | 0.219542 | 0.677045 | -5.62903 | 3.77E-12 | epiphycan |
| HOXC6 | protein coding | -0.64141 | 0.412548 | -5.73884 | 1.57E-07 | homeobox C6 |
| APOH | protein coding | -0.76922 | 0.408381 | -5.96962 | 2.18E-06 | apolipoprotein H |
| APOB | protein coding | -0.61539 | 0.534599 | -7.01039 | 2.26E-07 | apolipoprotein B |
| AHSG | protein coding | -0.6234 | 0.407763 | -8.38632 | 4.89E-11 | alpha 2-HS glycoprotein |
| AFP | protein coding | -0.0559 | 0.893362 | -8.40866 | 0.893362 | alpha fetoprotein |
