# Supplementary material for: Transcriptomic Signature of Human Embryonic Thyroid Reveals Transition From Differentiation to Functional Maturation
Source: Front Cell Dev Biol. 2021 Jun 11;9:669354. doi: 10.3389/fcell.2021.669354 (PMC8270686; doi:10.3389/fcell.2021.669354)
Supplement: Supplementary file 8 [file Table_7.docx]

# Supplementary table 7.

# Top20 thyroid-related differentially-regulated genes

## File description

The file contains lists of 20 genes and non-annotated probes with strongest fold change specifically up-or downregulated in the course of human thyroid development.

These lists originate from the **Intersection 2** (comparison of developmentally-regulated genes in the thyroid and non-thyroid tissues, for details see **Supplementary file 2. Supplementary results and discussion.docx**)

Contents

[File 8. Top20 thyroid-related differentially-regulated genes (.docx) 1](#_Toc62800313)

[File description 1](#_Toc62800314)

[Group 1. Genes co-regulated in the course of thyroid and non-thyroid development 2](#_Toc62800315)

[Group 2. Genes up- or downregulated in the course of development of the thyroid but not other organs. 3](#_Toc62800316)

## Group 1. Genes co-regulated in the course of thyroid and non-thyroid development

|  | Intersection 2 | | | | | | |
| --- | --- | --- | --- | --- | --- | --- | --- |
|  | comparison 1 (AT vs. ET) | | |  | comparison 3 (AM vs. EM) | | |
| co-upregulated | Adult thyroid (AT)  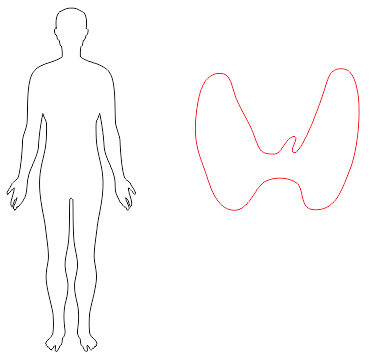 | > | Embryonic thyroid (ET)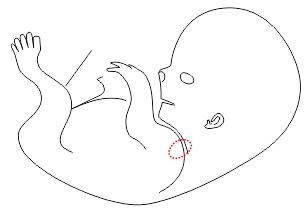 | AND | Adult tissue mix (AM)  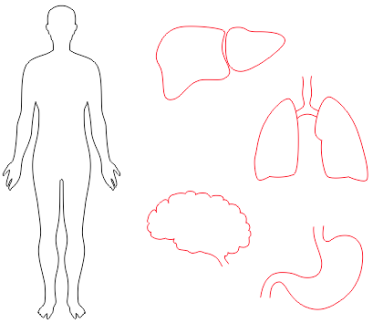 | > | Embryonic tissue mix (EM)  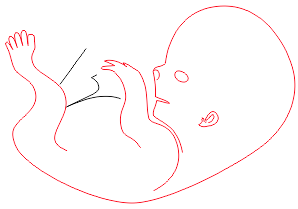 |
|  |  | | | | | | |
| co-dowreg. | Adult thyroid (AT)  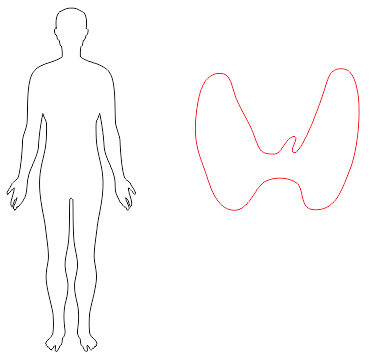 | < | Embryonic thyroid (ET)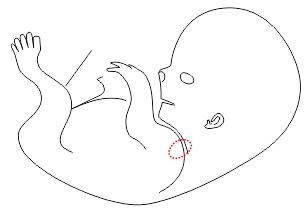 | AND | Adult tissue mix (AM)  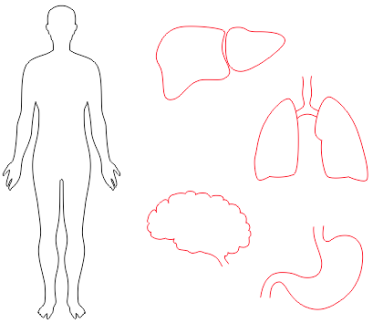 | < | Embryonic tissue mix (EM)  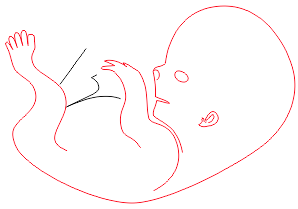 |
|  | Group 1 contains top 20 genes differentially expressed and upregulated (adj.p-value < 0.05; log2FC > 1) or downregulated (adj.p-value < 0.05; log2FC < -1) in both adult thyroid (AT) and adult tissue mix (AM) compared to embryonic thyroid (ET) and embryonic tissue mix (EM) respectively. | | | | | | |

**Table 1.1.A** Top 20 protein-coding genes co-upregulated in the course of development of thyroid and other organs.

|  | ### | ATET | ATET | AMEM | AMEM | ### |
| --- | --- | --- | --- | --- | --- | --- |
|  | gene type | logFC | adj.P.Val | logFC | adj.P.Val | gene description |
| IGHG1,IGHG2, IGHG3,IGHM, IGHV4-31 | IG_C_gene, IG_V_gene | 6.458315 | 1.24E-05 | 6.253945 | 3.2E-07 | 0 |
| HLA-DRA | protein coding | 5.598742 | 3.85E-10 | 5.497661 | 2.11E-12 | major histocompatibility complex, class II, DR alpha |
| ADH1B | protein coding | 6.052636 | 4.63E-06 | 4.890433 | 3.56E-06 | alcohol dehydrogenase 1B (class I), beta polypeptide |
| IFI27 | protein coding | 5.000299 | 8.47E-13 | 5.56371 | 4.43E-17 | interferon alpha inducible protein 27 |
| IGLC1,IGLJ3 | IG_C_gene, IG_J_gene | 5.140607 | 0.000552 | 5.407932 | 7.49E-06 | 0 |
| JCHAIN | protein coding | 5.440888 | 0.000528 | 4.975144 | 6.42E-05 | joining chain of multimeric IgA and IgM |
| HLA-DPA1 | protein coding | 4.332157 | 4.07E-08 | 5.987627 | 2.19E-13 | major histocompatibility complex, class II, DP alpha 1 |
| HLA-DQA1,HLA-DQA2 | protein coding | 5.216224 | 5.14E-07 | 5.051029 | 8.13E-09 | major histocompatibility complex, class II, DQ alpha 1,2 |
| ADIRF | protein coding | 6.028724 | 2.94E-12 | 4.163214 | 2.94E-10 | adipogenesis regulatory factor |
| IGHA1,2 | IG_C_gene | 3.823173 | 0.020299 | 6.098275 | 3.43E-06 | 0 |
| HLA-DRB3 | protein coding | 3.993732 | 2.63E-07 | 5.401997 | 4.53E-13 | major histocompatibility complex, class II, DR beta 3 |
| IGKC | IG_C_gene | 4.41421 | 0.000245 | 4.74637 | 1.66E-06 | 0 |
| HLA-DQB1, HLA-DRB1,HLA-DRB4,HLA-DRB5 | protein coding | 4.008525 | 4.38E-08 | 5.132803 | 2.03E-13 | major histocompatibility complex, class II, DQ beta 1, DR beta 1,4,5 |
| HSD17B6 | protein coding | 5.86443 | 9.96E-07 | 3.231673 | 0.000337 | hydroxysteroid 17-beta dehydrogenase 6 |
| FGL2 | protein coding | 4.516517 | 8.07E-10 | 3.788008 | 5.72E-08 | fibrinogen like 2 |
| HLA-DMA | protein coding | 3.555459 | 2.75E-06 | 4.72932 | 2.05E-11 | major histocompatibility complex, class II, DM alpha |
| TRIM22 | protein coding | 3.808394 | 7.03E-09 | 4.447107 | 3.25E-13 | tripartite motif containing 22 |
| IGLV1-44 | IG_V_gene | 4.038805 | 0.001339 | 4.182397 | 3.91E-05 | 0 |
| STEAP4 | protein coding | 4.338733 | 1.24E-06 | 3.653199 | 4.43E-07 | STEAP4 metalloreductase |
| BHLHE41 | protein coding | 4.517146 | 6.9E-13 | 3.452755 | 3.67E-08 | basic helix-loop-helix family member e41 |

**Table 1.1.B** Top 20 protein-coding, genes co-downregulated in the course of development of thyroid and other organs.

|  | ### | ATET | ATET | AMEM | AMEM | ### |
| --- | --- | --- | --- | --- | --- | --- |
|  | gene type | logFC | adj.P.Val | logFC | adj.P.Val | gene description |
| ASPM | protein coding | -4.30229 | 8.69E-20 | -5.05639 | 1.39E-12 | abnormal spindle microtubule assembly |
| TUBB2B | protein coding | -4.87312 | 5.63E-05 | -4.54647 | 3.66E-06 | tubulin beta 2B class IIb |
| STMN2 | protein coding | -3.92199 | 0.005166 | -5.57504 | 4.83E-07 | stathmin 2 |
| KIF20A | protein coding | -5.75984 | 9.28E-12 | -3.74808 | 3.65E-09 | kinesin family member 20A |
| PBK | protein coding | -5.69302 | 2.52E-07 | -3.98481 | 3.51E-06 | PDZ binding kinase |
| COL11A1 | protein coding | -3.46467 | 2.85E-06 | -6.36135 | 1.38E-19 | collagen type XI alpha 1 chain |
| PRSS35 | protein coding | -5.83041 | 9.2E-19 | -4.11748 | 1.93E-16 | protease, serine 35 |
| SULT1E1 | protein coding | -5.61713 | 4.01E-15 | -4.75024 | 1.59E-15 | sulfotransferase family 1E member 1 |
| MALSU1 | protein coding | -5.0554 | 4.39E-15 | -5.40071 | 6.88E-19 | mitochondrial assembly of ribosomal large subunit 1 |
| DLGAP5 | protein coding | -5.88112 | 1.11E-11 | -4.8911 | 6.82E-12 | DLG associated protein 5 |
| FBN2 | protein coding | -4.68475 | 5.71E-09 | -6.43852 | 1.59E-15 | fibrillin 2 |
| DCX | protein coding | -4.03788 | 0.000101 | -7.25707 | 1.59E-12 | doublecortin |
| COL2A1 | protein coding | -4.46675 | 5.68E-08 | -7.07999 | 1.25E-18 | collagen type II alpha 1 chain |
| TOP2A | protein coding | -7.14192 | 1.93E-11 | -5.54073 | 7.92E-11 | topoisomerase (DNA) II alpha |
| SOX11 | protein coding | -5.91121 | 3.92E-09 | -7.56468 | 1.03E-14 | SRY-box 11 |
| HBZ | protein coding | -5.75376 | 1.66E-12 | -8.31825 | 1.51E-20 | hemoglobin subunit zeta |
| DLK1 | protein coding | -7.90473 | 1.91E-10 | -6.34142 | 2.76E-10 | delta like non-canonical Notch ligand 1 |
| HBG1,2 | protein coding | -7.52391 | 1.53E-13 | -6.91117 | 4.89E-15 | hemoglobin subunit gamma 1,2 |
| HBE1 | protein coding | -6.56733 | 2.24E-19 | -8.37737 | 1.34E-26 | hemoglobin subunit epsilon 1 |
| HMGA2 | protein coding | -8.16152 | 1.3E-26 | -7.53404 | 1.97E-28 | high mobility group AT-hook 2 |

## Group 2. Genes up- or downregulated in the course of development of the thyroid but not other organs.

| Intersection 2 | | | | | | |
| --- | --- | --- | --- | --- | --- | --- |
| comparison 1 (AT vs. ET) | | |  | comparison 3 (AM vs. EM) | | |
| Adult thyroid (AT)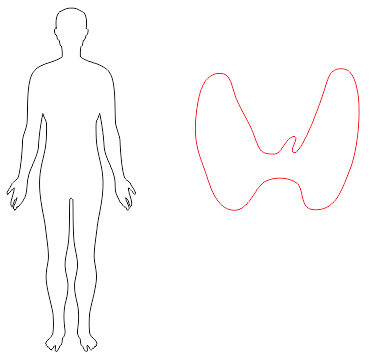 | > | Embryonic thyroid (ET)  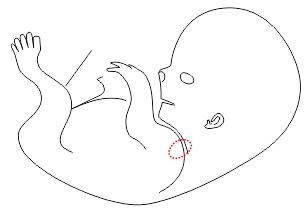 | AND | Adult tissue mix (AM)  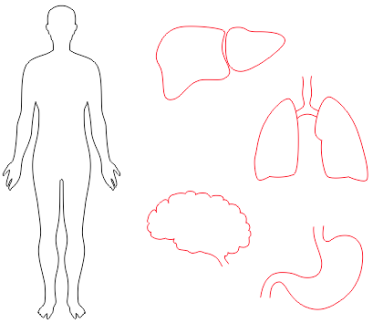 | =  < | Embryonic tissue mix (EM)  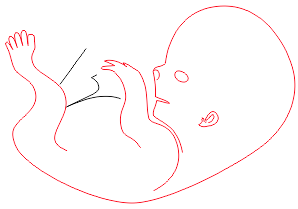 |
|  | | | | | | |
| Adult thyroid (AT)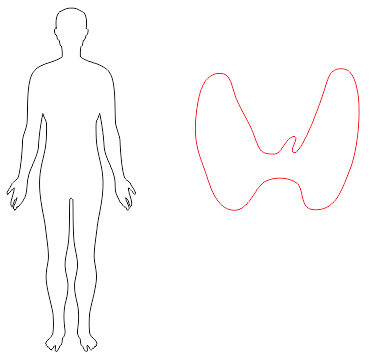 | < | Embryonic thyroid (ET)  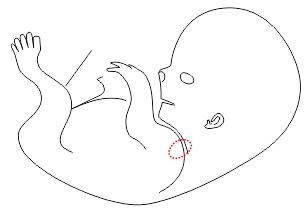 | AND | Adult tissue mix (AM)  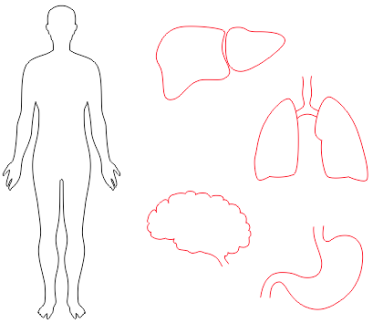 | =  > | Embryonic tissue mix (EM)  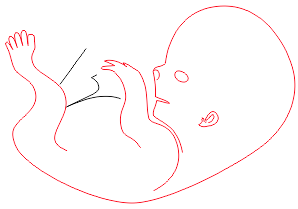 |
| Group 2 contains top 20 genes differentially expressed and upregulated (adj.p-value < 0.05; log2FC > 1) or downregulated (adj.p-value < 0.05; log2FC < -1) in adult thyroid (AT) but not adult tissue mix (AM) compared to embryonic thyroid (ET) and embryonic tissue mix (EM) respectively. | | | | | | |

**Table 2.1.A** Top 20 protein-coding genes upregulated in the course of development of the thyroid but not other other organs.

|  | ### | ATET | ATET | AMEM | AMEM |  | ### |
| --- | --- | --- | --- | --- | --- | --- | --- |
|  | gene type | logFC | adj.P.Val | logFC | adj.P.Val | function | gene description |
| ADGRF1 | protein coding | 3.059095 | 8.47E-13 | -0.313 | 0.016672 | signaling | adhesion G protein-coupled receptor F1 |
| ANXA1 | protein coding | 4.561993 | 5.39E-13 | 0.163469 | 0.835394 |  | annexin A1 |
| ANXA9 | protein coding | 3.041881 | 1.23E-05 | 0.468792 | 0.406431 |  | annexin A9 |
| ART4 | protein coding | 3.601732 | 4.98E-07 | -0.3219 | 0.531494 | transport | ADP-ribosyltransferase 4 (Dombrock blood group) |
| CCDC85A | protein coding | 3.276161 | 1.45E-05 | -0.45029 | 0.004411 |  | coiled-coil domain containing 85A |
| CLIC6 | protein coding | 3.342391 | 0.000473 | 0.302816 | 0.424508 | channel (metabolism?) | chloride intracellular channel 6 |
| CTGF | protein coding | 3.055785 | 3.33E-05 | -0.32877 | 0.602542 | signaling.L | connective tissue growth factor |
| DAPL1 | protein coding | 4.1539 | 5.49E-12 | 0.007554 | 0.988119 |  | death associated protein like 1 |
| DGKI | protein coding | 4.05744 | 5.11E-11 | -1.40857 | 0.001045 | signaling | diacylglycerol kinase iota |
| DIO1 | protein coding | 5.532955 | 1.81E-06 | -0.75414 | 0.415828 | thyr.horm | iodothyronine deiodinase 1 |
| ESR1 | protein coding | 3.667176 | 3.35E-05 | -0.63711 | 0.000605 | gene exprs. | estrogen receptor 1 |
| GPM6A | protein coding | 4.184184 | 0.001675 | -2.45222 | 0.014743 |  | glycoprotein M6A |
| MT1F | protein coding | 3.616628 | 5.56E-07 | -1.26273 | 0.029219 | transport (thyr. horm) | metallothionein 1F |
| MT1G | protein coding | 3.909604 | 1.69E-06 | -1.49988 | 0.013234 | transport (thyr. horm) | metallothionein 1G |
| MT1H | protein coding | 3.411303 | 2.15E-06 | -1.19966 | 0.02497 | transport (thyr. horm) | metallothionein 1H |
| OMD | protein coding | 3.4676 | 7.82E-08 | -0.38175 | 0.429208 | cytosk/ECM | osteomodulin |
| PKHD1L1 | protein coding | 4.73642 | 2.25E-12 | 0.159027 | 0.753041 |  | PKHD1 like 1 |
| SCNN1A | protein coding | 3.378412 | 0.002323 | -0.93427 | 1.29E-05 | channel | sodium channel epithelial 1 alpha subunit |
| TNFRSF11B | protein coding | 6.497558 | 1.17E-19 | 0.318496 | 0.400785 | signaling | TNF receptor superfamily member 11b |
| TPO | protein coding | 5.091257 | 2.91E-16 | 0.009816 | 0.98294 | thyr.horm | thyroid peroxidase |

**Table 2.1.B** Top 20 protein-coding, genes downregulated in the course of development of the thyroid but not other other organs.

|  | ### | ATET | ATET | AMEM | AMEM | ### |
| --- | --- | --- | --- | --- | --- | --- |
|  | gene type | logFC | adj.P.Val | logFC | adj.P.Val | gene description |
| SHC4 | protein coding | -2.39938 | 2.33E-07 | -0.31551 | 0.419383 | SHC adaptor protein 4 |
| TFPI2 | protein coding | -2.41431 | 0.003412 | 0.165867 | 0.653467 | tissue factor pathway inhibitor 2 |
| CHRNA3 | protein coding | -2.43968 | 0.000724 | -0.50269 | 0.397646 | cholinergic receptor nicotinic alpha 3 subunit |
| TCF24 | protein coding | -2.61089 | 2.93E-19 | -0.10272 | 0.524231 | transcription factor 24 |
| NFE2L3 | protein coding | -2.62899 | 3.18E-08 | 0.773503 | 0.022223 | nuclear factor, erythroid 2 like 3 |
| ZNF367 | protein coding | -2.67073 | 2.75E-05 | -0.43185 | 0.405529 | zinc finger protein 367 |
| SPON1 | protein coding | -2.68486 | 0.001152 | -0.49851 | 0.377684 | spondin 1 |
| SMTNL2 | protein coding | -2.71438 | 0.000388 | -0.47051 | 0.460917 | smoothelin like 2 |
| FGF12 | protein coding | -2.7913 | 4.59E-06 | 0.184753 | 0.767917 | fibroblast growth factor 12 |
| GREB1 | protein coding | -2.80093 | 0.002414 | -0.36092 | 0.655237 | growth regulation by estrogen in breast cancer 1 |
| CEACAM6 | protein coding | -2.86184 | 0.022933 | 2.392032 | 0.016979 | carcinoembryonic antigen related cell adhesion molecule 6 |
| DCAF12L1 | protein coding | -2.93773 | 1.04E-07 | -0.01204 | 0.981925 | DDB1 and CUL4 associated factor 12 like 1 |
| SLC22A3 | protein coding | -2.95295 | 0.000625 | 2.037129 | 0.002219 | solute carrier family 22 member 3 |
| NRXN3 | protein coding | -3.08023 | 4.01E-05 | 0.294528 | 0.528916 | neurexin 3 |
| GLRB | protein coding | -3.08646 | 6.9E-05 | 0.871756 | 0.037354 | glycine receptor beta |
| CHRNA9 | protein coding | -3.48772 | 2.98E-21 | -0.09203 | 0.635968 | cholinergic receptor nicotinic alpha 9 subunit |
| GPR37 | protein coding | -3.60168 | 0.001283 | 0.402791 | 0.586457 | G protein-coupled receptor 37 |
| FREM3 | protein coding | -3.76307 | 2.21E-14 | -0.06654 | 0.853304 | FRAS1 related extracellular matrix 3 |
| SCGB3A2 | protein coding | -3.98295 | 0.007907 | -0.45232 | 0.734705 | secretoglobin family 3A member 2 |
| PCDH20 | protein coding | -4.77647 | 7.64E-12 | -0.12444 | 0.818915 | protocadherin 20 |
